# Supplementary figures and images for: SCRINSHOT enables spatial mapping of cell states in tissue sections with single-cell resolution
Source: PLoS Biol. 2020 Nov 20;18(11):e3000675. doi: 10.1371/journal.pbio.3000675 (PMC7717588; doi:10.1371/journal.pbio.3000675)

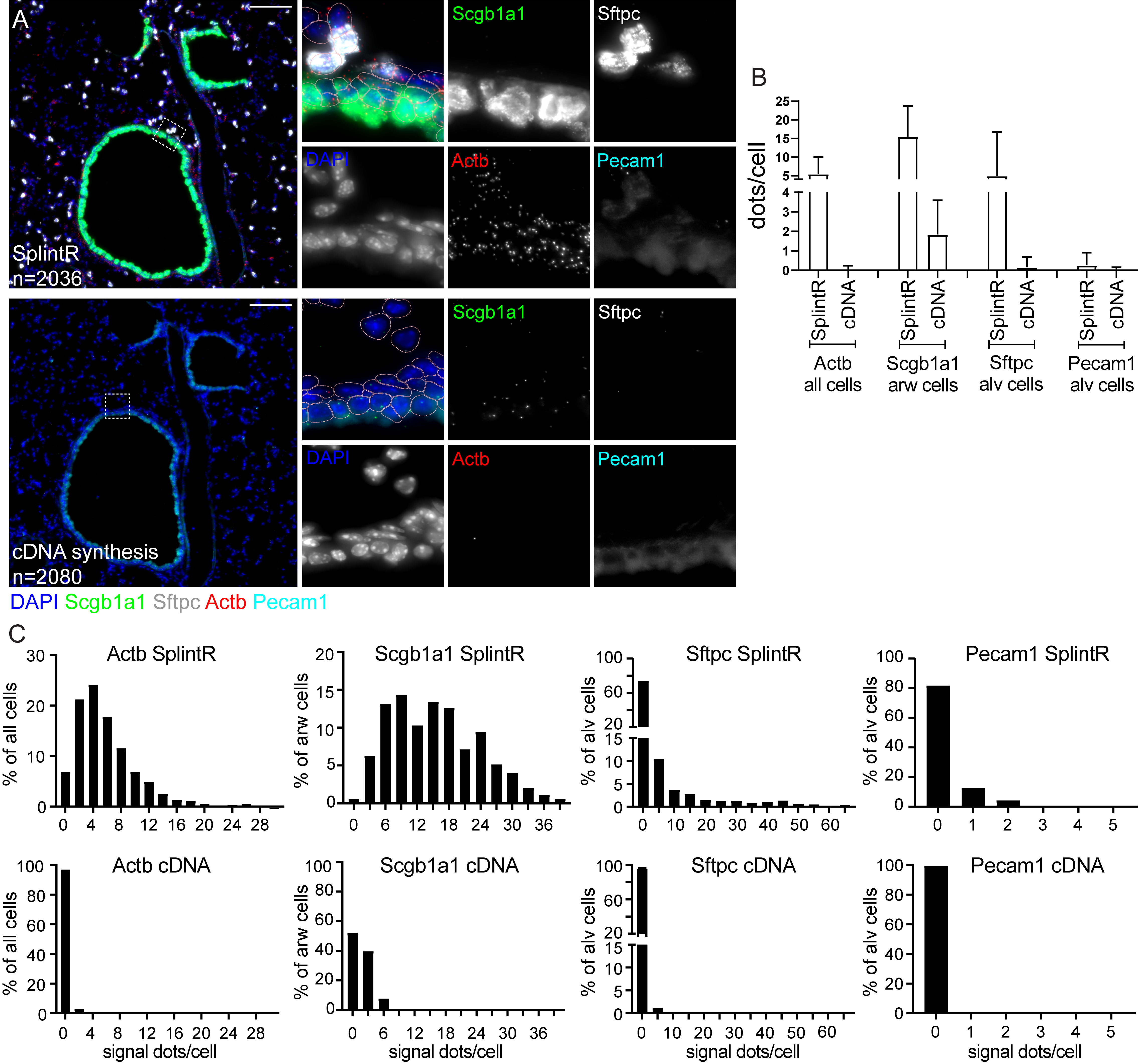

Supplement: S1 Fig — (A) Images of SplintR-based (SCRINSHOT) and cDNA-based in situ hybridization assays. DAPI: blue, Scgb1a1: green, Sftpc: gray, Actb: red, and Pecam1: cyan. Pink outlines show the 2-μm expanded airway nuclear ROIs, which are considered as cells. The square brackets indicate the magnified areas on the right. The “n” corresponds to the number of counted cells in large images. Scale bar: 100 μm. (B) Bar-plots of the analyzed gene signals, in the indicated tissue compartments, for SCRINSHOT and cDNA-based approaches. The differences between the 2 conditions are significant (P < 0.0001) for all analyzed genes. (C) Histograms of the analyzed genes. The y-axes indicate the percentage of the cell ROIs and the x-axes, the binned signal dots in each cell. In SplintR-condition, 350 cells localized in arw and 1,624 in alv compartment. In cDNA-condition, there are 295 airway and 1,706 alveolar cells. Analysis was done using raw images, with the same acquisition conditions and thresholds. Only for visualization purposes, signal intensity of Scgb1a1 and Sftpc in cDNA-condition was set 5-times higher than SplintR. The data underlying this figure can be found in 10.5281/zenodo.3634561. alv, alveolar; arw, airway; cDNA, complementary DNA; PFA, paraformaldehyde-fixed; ROI, region of interest. (TIF) [file pbio.3000675.s001.tif]

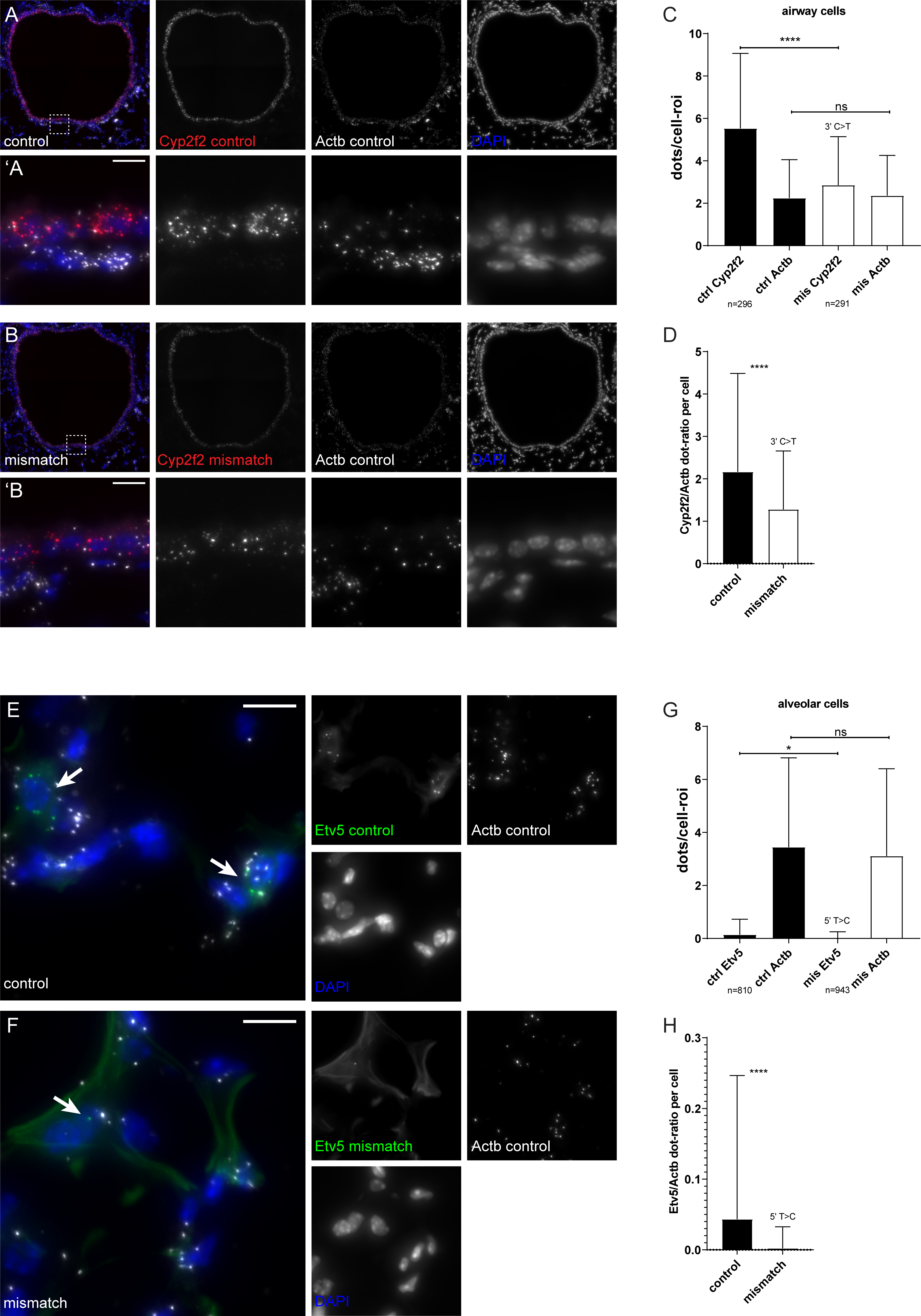

Supplement: S2 Fig — (A) SCRINSHOT signal dots of the control Cyp2f2 padlock probe. (‘A) indicates the area of square bracket in “A”. (B) SCRINSHOT signal dots of the mutated (C>T at the 3′-arm) Cyp2f2 padlock probe. (‘B) indicates the area of square bracket in “B”. The same Actb padlock probe was used in both conditions, as internal control, showing no statistically significant difference. DAPI: blue, Cyp2f2: red, Actb: gray. Scalebar: 10 μm. (C) Bar-plot of the SCRINSHOT dots per cell in all airway cells of the analyzed tissue sections (A and B). (D) Bar-plot of the Cyp2f2/Actb dot ratio in the control and mismatch conditions. (E) SCRINSHOT signal dots of the control Etv5 padlock probe. (F) SCRINSHOT signal dots of the mutated (T>C at the 5′-arm) Etv5 padlock probes. The same Actb padlock probe was used in both conditions, showing no statistically significant difference. DAPI: blue, Etv5: green, Actb: gray. Scalebar: 10 μm. (G) Bar-plot of the SCRINSHOT dots per cell, in all alveolar cells (E and F). (H) Bar-plot of the Etv5/Actb dot ratio in the control and mismatch conditions. Arrows indicate Etv5pos cells. The “n” values indicate the number of analyzed cells in each tissue section. The data underlying this figure can be found in 10.5281/zenodo.3978632. ns, not significant. (TIF) [file pbio.3000675.s002.tif]

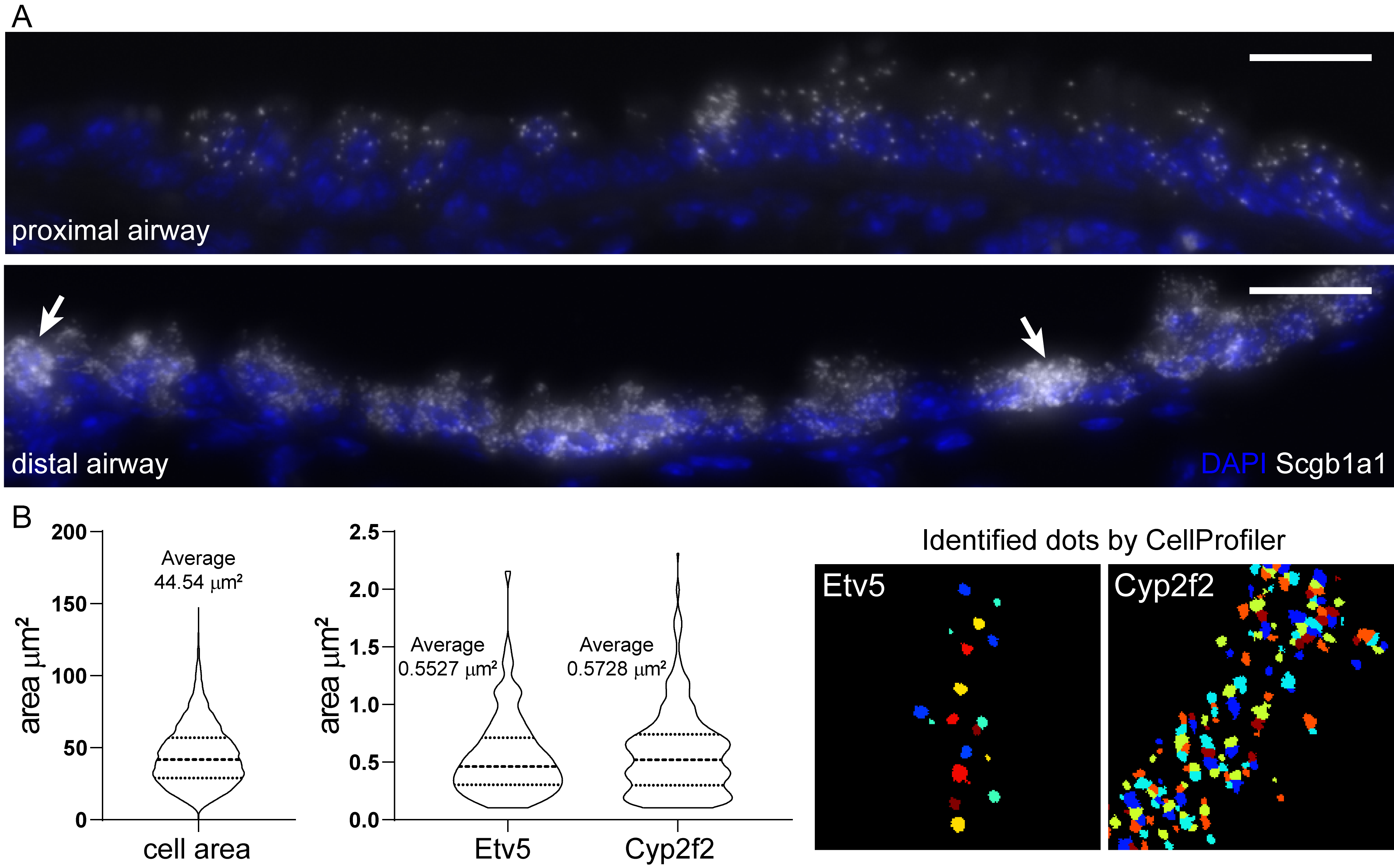

Supplement: S3 Fig — (A) Raw images of proximal (top) and distal (bottom) airway epithelium, showing the Scgb1a1 SCRINSHOT dots. The arrows indicate areas with signal saturation, which are present only in distal airways. DAPI: blue, Scgb1a1: gray. Scalebar: 20 μm. (B) Violin plots of the average cell surface, as indicated by the 2-μm expanded nuclei and SCRINSHOT dot surfaces of Etv5 (alveolar) and Cyp2f2 (airway). Representative images of the recognized SCRINSHOT dots by CellProfiler custom script, that labels the identified dots according to the CellProfiler default colormap. The data underlying this figure can be found in 10.5281/zenodo.3978632. (TIF) [file pbio.3000675.s003.tif]

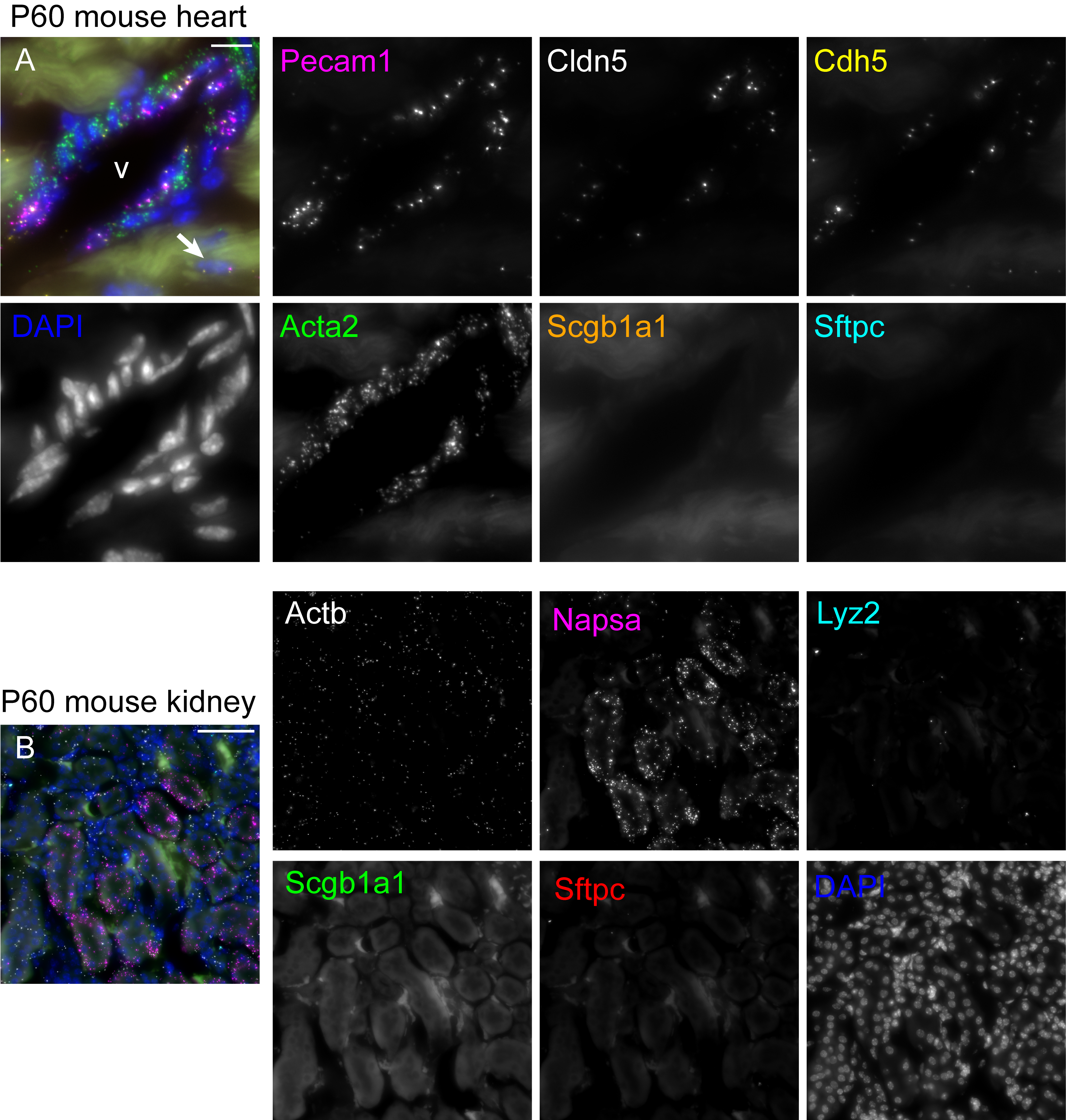

Supplement: S4 Fig — (A) Application of SCRINSHOT in adult mouse heart section, containing a vessel (v). The endothelial cell markers Pecam1 (magenta), Cldn5 (gray), and Cdh5 (yellow) were detected close to the lumen of the vessel, where this cell type is normally located. Acta2 (green) was detected at the inner thick part of the vessel wall, being consistent with the expression of the marker by vascular smooth muscle cells. Scalebar: 10 μm. Epithelial markers Sftpc (cyan) and Scgb1a1 (orange) were not detected. The data underlying this figure section can be found in 10.5281/zenodo.3978632. (B) Representative image from adult mouse kidney sections shows signal for Actb (gray), Napsa (magenta), and Lyz2 (cyan) but not Scgb1a1 (green) and Sftpc (red). Scalebar: 50 μm. DAPI (blue) was used for nuclear staining in both images. The data underlying this figure section can be found in 10.5281/zenodo.3634561. (TIF) [file pbio.3000675.s004.tif]

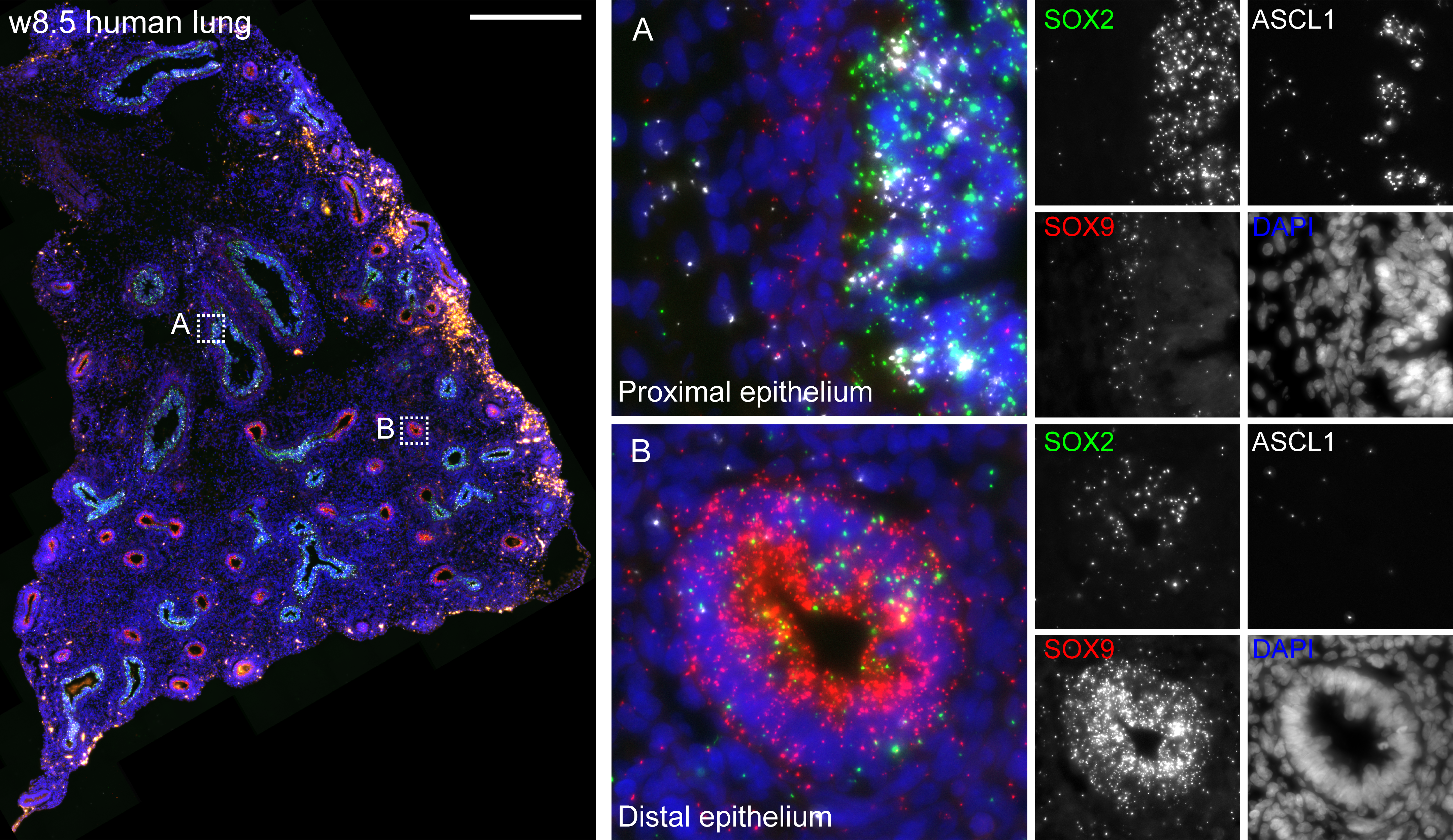

Supplement: S5 Fig — On the left, overview of a w8.5 whole left lung tissue section, showing SCRINSHOT signal for SOX2 (green), SOX9 (red), and ASCL1 (gray). The square brackets correspond to the images on the right. DAPI (blue) was used as nuclear staining. Scale bar: 500 μm. (A) Representative image of proximal epithelium, which is highly positive of SOX2 and ASCL1 but not SOX9. (B) Representative image of highly SOX9 positive distal epithelium. The data underlying this figure can be found in 10.5281/zenodo.3634561. (TIF) [file pbio.3000675.s005.tif]

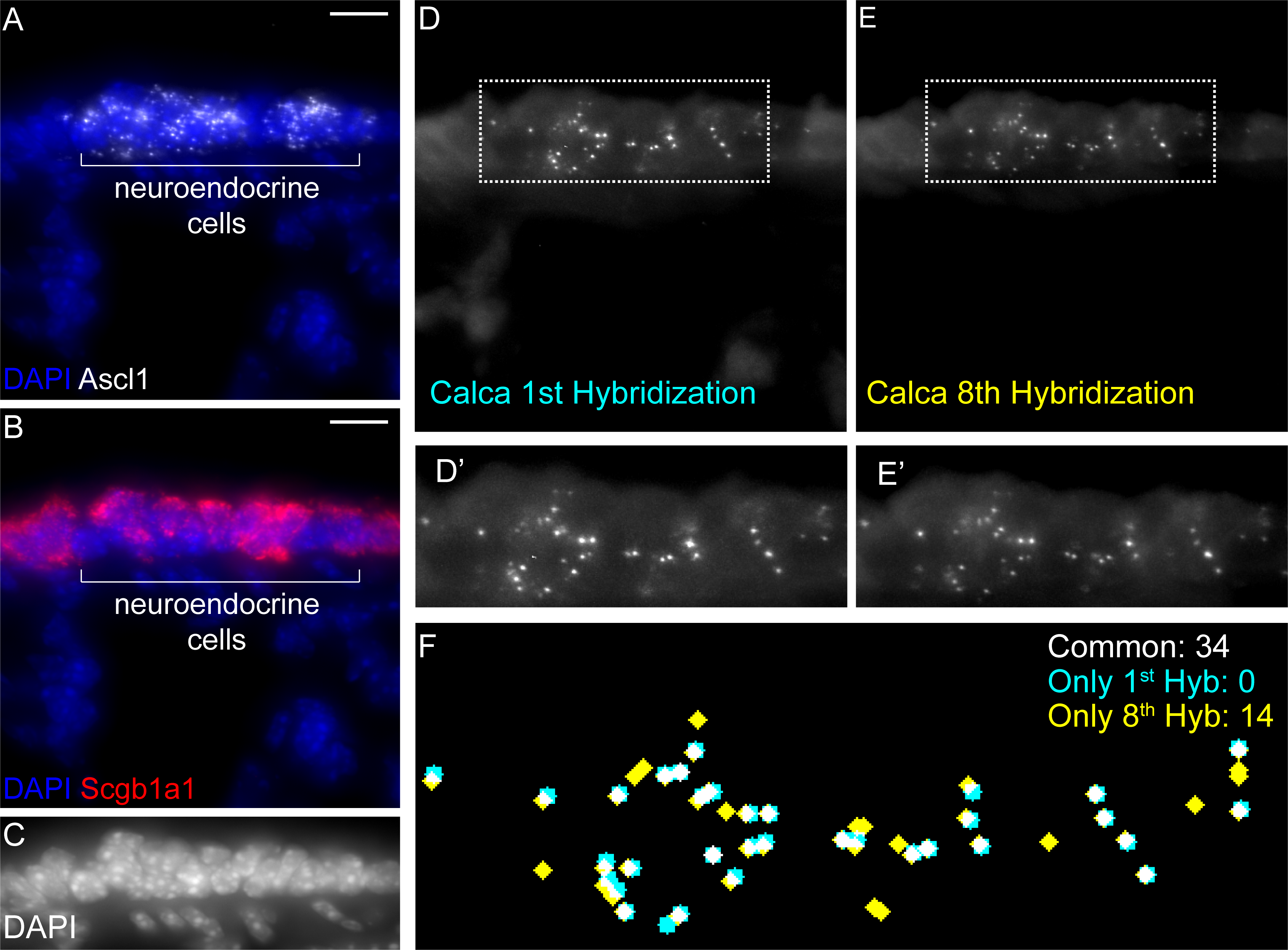

Supplement: S6 Fig — (A, B) Images on the left show the Ascl1pos (gray) neuroendocrine cells of an airway neuroepithelial body, in relation to Scgb1a1pos (red) club cells. DAPI: blue, scale bar: 10μm. (C) Note that neuroepithelial bodies are tightly packed cellular structures, as indicated by DAPI nuclear staining. The images on the right show the Calca RCA-products, detected in the first (D) and the eighth (E) detection cycles. (D’-F’) Magnified areas of the indicated positions (brackets) of images D–F, respectively. (F) Overlay of Calca identified signal dots in first (cyan) and eighth (yellow) detection cycles, using the same threshold in CellProfiler. The data underlying this Figure can be found in 10.5281/zenodo.3634561. RCA, rolling circle amplification. (TIF) [file pbio.3000675.s006.tif]

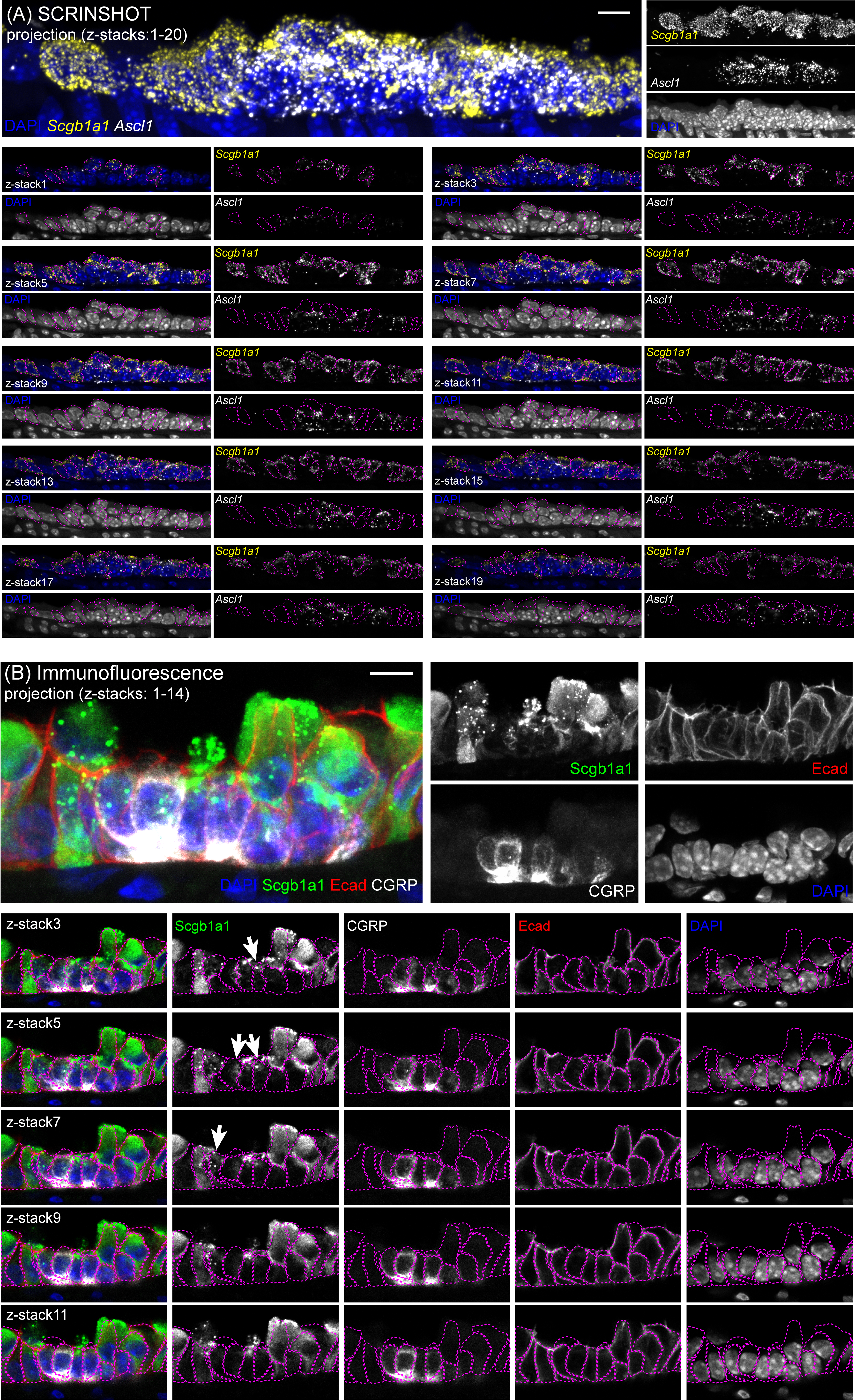

Supplement: S7 Fig — Image of the same NEB in S6 Fig, showing SCRINSHOT signal dots of Scgb1a1 and Ascl1. (Top) Maximal orthogonal projection of 20 z-stacks. (Bottom) Individual z-stacks. Cell outlines (magenta) were based on Scgb1a1 SCRINSHOT signal. DAPI: blue, Scgb1a1: yellow, Ascl1: gray. Scalebar: 5 μm. (B) Immunofluorescence of a representative NEB from an adult mouse lung. Club cells were stained with an anti-Scgb1a1 antibody and NE cells with an anti-CGRP antibody. Epithelial cell membrane was stained with an anti-E-cadherin antibody. (Top) Maximal orthogonal projection of 13 z-stacks. (Bottom) Individual z-stacks. Cell outlines, based on E-cadherin signal, are shown with magenta. Both images were acquired with optimal confocal microscopy settings, using a 63× oil-immersion lens. DAPI: blue, Scgb1a1: green, E-cadherin: red, CGRP: gray. Scalebar: 5 μm. The data underlying this Figure can be found in 10.5281/zenodo.3978632. CGRP, calcitonin gene-related peptide; NE, neuroendocrine; NEB, neuroepithelial body. (TIF) [file pbio.3000675.s007.tif]

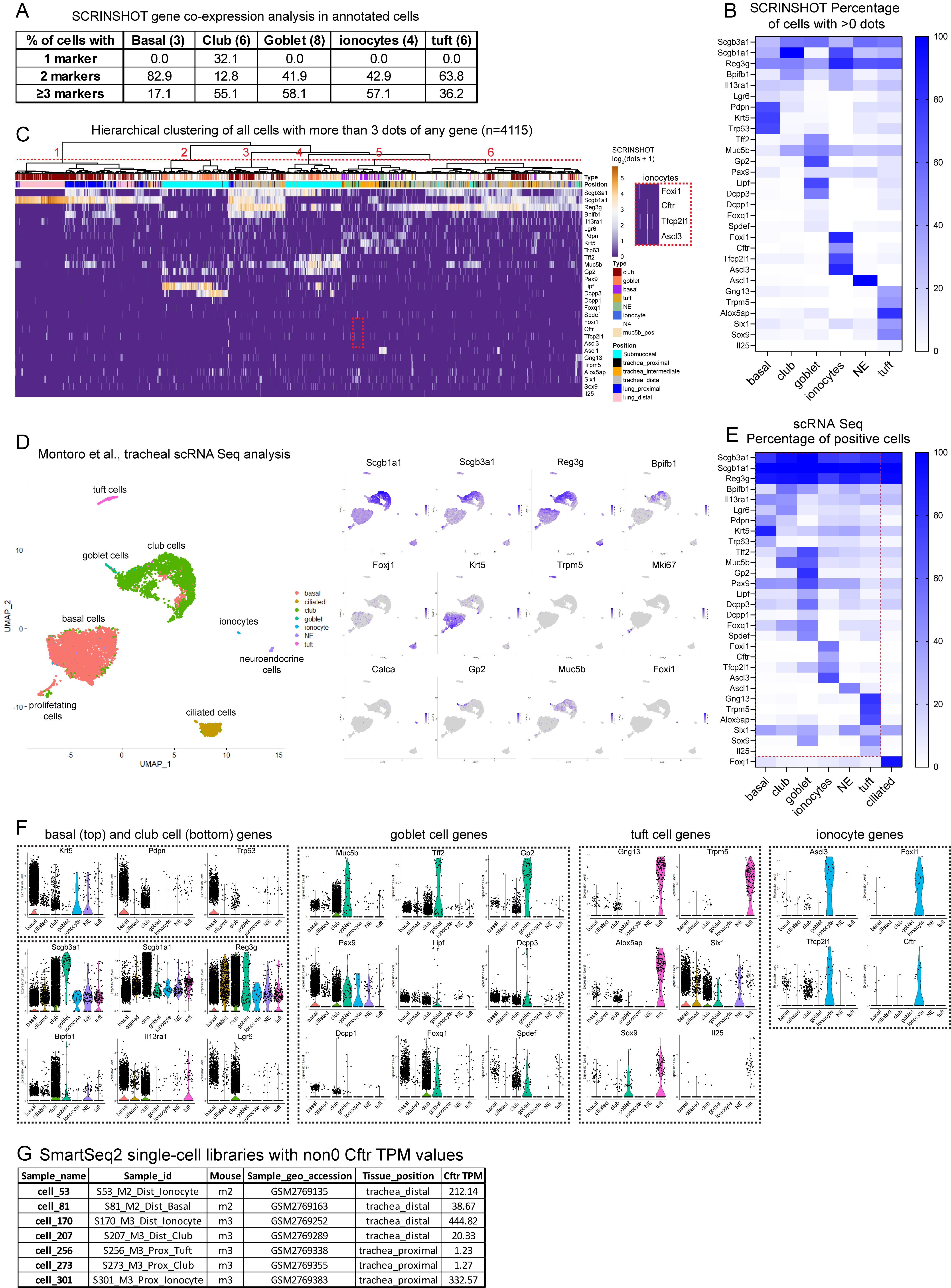

Supplement: S8 Fig — (A) Table showing the percentage of the cells of the indicated cell types, which express 1, 2, and 3 or more markers of the indicated cell type. Numbers in the parentheses show the number of used markers for each cell type. (B) Heatmap of the percentage of cells of the annotated cell types, that express one or more SCRINSHOT dots of the analyzed genes. (C) Hierarchical clustering of all analyzed cells with more than 3 dots of any gene and combination, without application of any cell size criteria (n = 4,115 cells). The heatmap shows the log2(dots+1) SCRINSHOT-detected dots for the corresponding genes. Red insert shows 4 clustered ionocytes. (D) Analysis of the droplet-based scRNA-Seq dataset, using Seurat v3.1 [59], with 2,000 most variable genes and the first 27 principle components. The original annotation of the dataset, from [52], was used for the gene expression analyses. Umap-plots show the gene expression pattern of cell-type representative markers. (E) Heatmap plot of the percentage of positive cells in each cell-type as indicated by Seurat (Raw counts > 0). (F) Violin plots of the scRNA-Seq droplet-based dataset, showing the gene expression levels of the used cell-type markers at the SCRINSHOT experiment. The dots indicate single cells. (G) Table of the Cftr TPM counts of SmartSeq2 scRNA-Seq dataset [52], that show low, but not zero, gene expression in basal, club, and tuft cells, in parallel to the high expression in the annotated ionocytes. The data underlying this figure can be found in 10.5281/zenodo.3978632. scRNA-Seq, single-cell RNA sequencing; TPM, transcripts per million. (TIF) [file pbio.3000675.s008.tif]

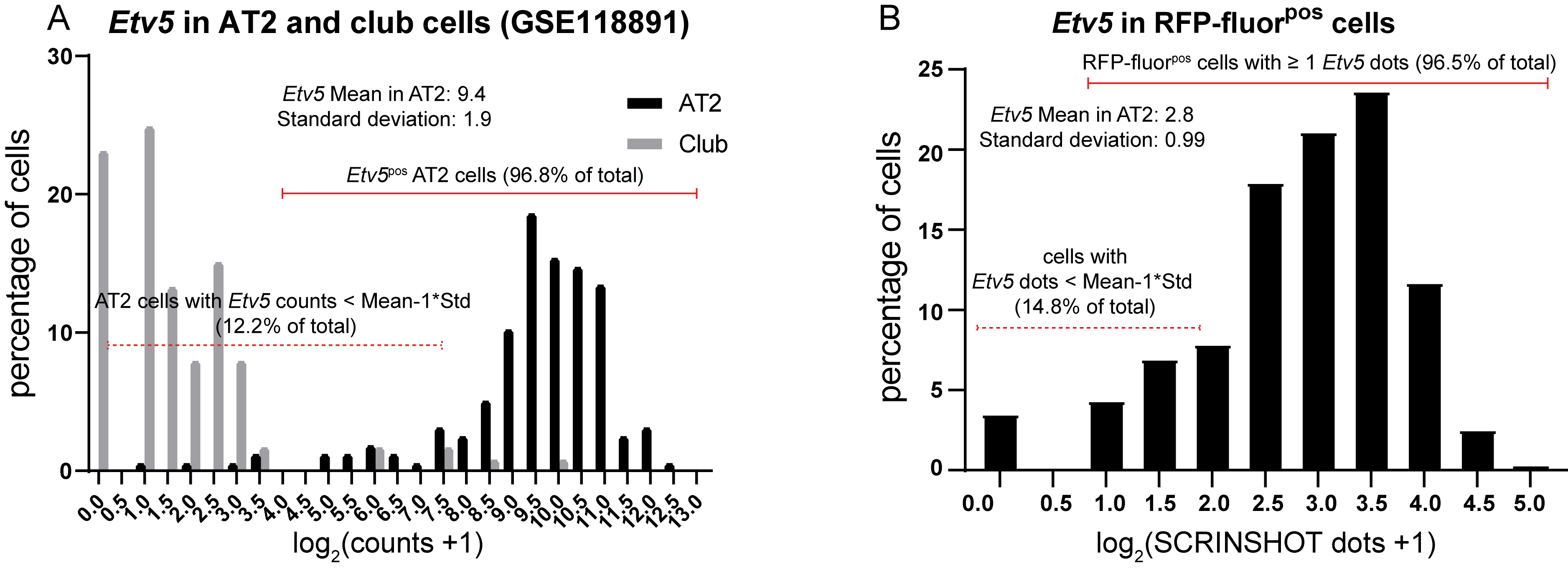

Supplement: S9 Fig — (A) Histogram of SmartSeq2 Etv5 log2(counts+1) values (x-axis), in the GSE118891 annotated AT2 (black) and club (gray) cells. y-axis indicates percentage of cells. Club cells were used to set the positivity threshold for Etv5, because 95% of them are found below value 4 (outside the continuous red line). Distribution of Etv5 counts in AT2 cells was used to estimate the low-expressing cells that are found below one standard deviation (1.9) from Etv5 count average (9.4) (dotted red line). (B) Same type of analysis, as in “A”, for Etv5 SCRINSHOT dots in RFP fluorescence positive cells of the Sftpc-CreERpos;Rosa-Ai14pos lung section, shows that 96.5% of RFP-fluorpos cells have ≥1 Etv5 dots, and 14.8% of them are detected with low number of dots (below 1 standard deviation from Etv5 SCRINSHOT dot average). The data underlying this figure can be found in 10.5281/zenodo.3978632. RFP, red fluorescent protein; scRNA-Seq, single-cell RNA sequencing. (TIF) [file pbio.3000675.s009.tif]

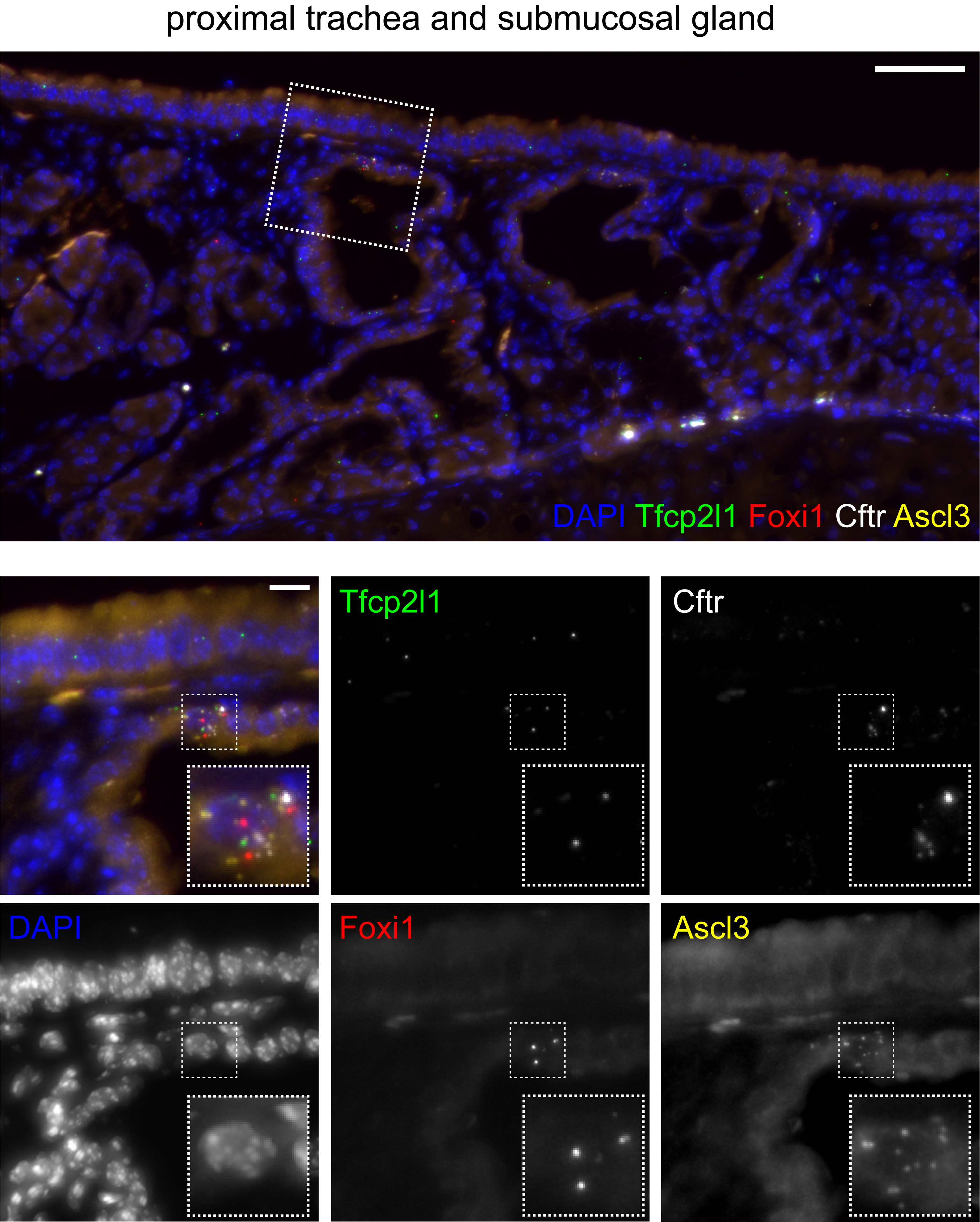

Supplement: S10 Fig — (Top) overview of the analyzed submucosal gland, showing SCRINSHOT signal for ionocyte markers, Tfcp2l1 (green), Cftr (gray), Foxi1 (red), Ascl3 (yellow), and DAPI (blue). Scale bar: 500 μm. (Bottom) Magnified area of the indicated square in overview image, showing a detected ionocyte in submucosal gland. Scale bar: 10 μm. The data underlying this Figure can be found in 10.5281/zenodo.3634561. (TIF) [file pbio.3000675.s010.tif]

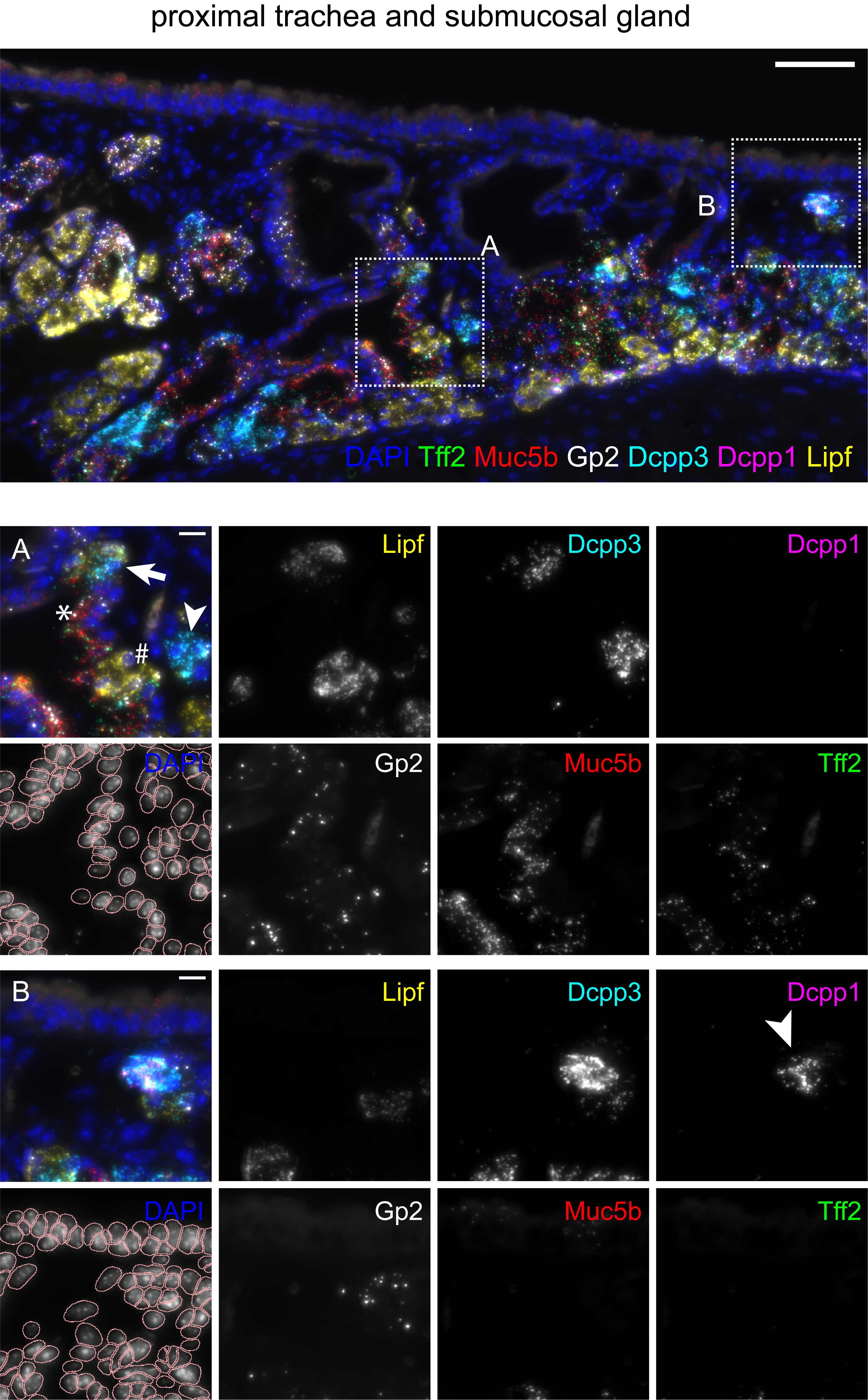

Supplement: S11 Fig — Overview of the analyzed submucosal gland for the expression of 6 goblet cell markers shows their expression in submucosal gland but not airway epithelium. Muc5b is detected along the airway epithelium of the proximal trachea, indicating that it is a general proximal epithelial cell marker. Tff2: green, Muc5b: red, Gp2: gray, Dcpp3: cyan, Dcpp1: magenta, Lipf: yellow, DAPI: blue, and cell ROIs: pink. Scale bar: 500 μm. (A) Insert showing the previously described Muc5bpos Tff2pos goblet subtype (arrow) and the Lipfpos Dcpp3pos (asterisk). Single Lipfpos (hash) and Dcpp3pos (arrowhead) are detected in the same region, being positive for the general goblet cell marker Gp2. (B) Insert showing regionally restricted expression of Dcpp1 in a subset of Dcpp3pos cells. Insert scale bar: 10 μm. The data underlying this figure can be found in 10.5281/zenodo.3634561. ROI, region of interest. (TIF) [file pbio.3000675.s011.tif]

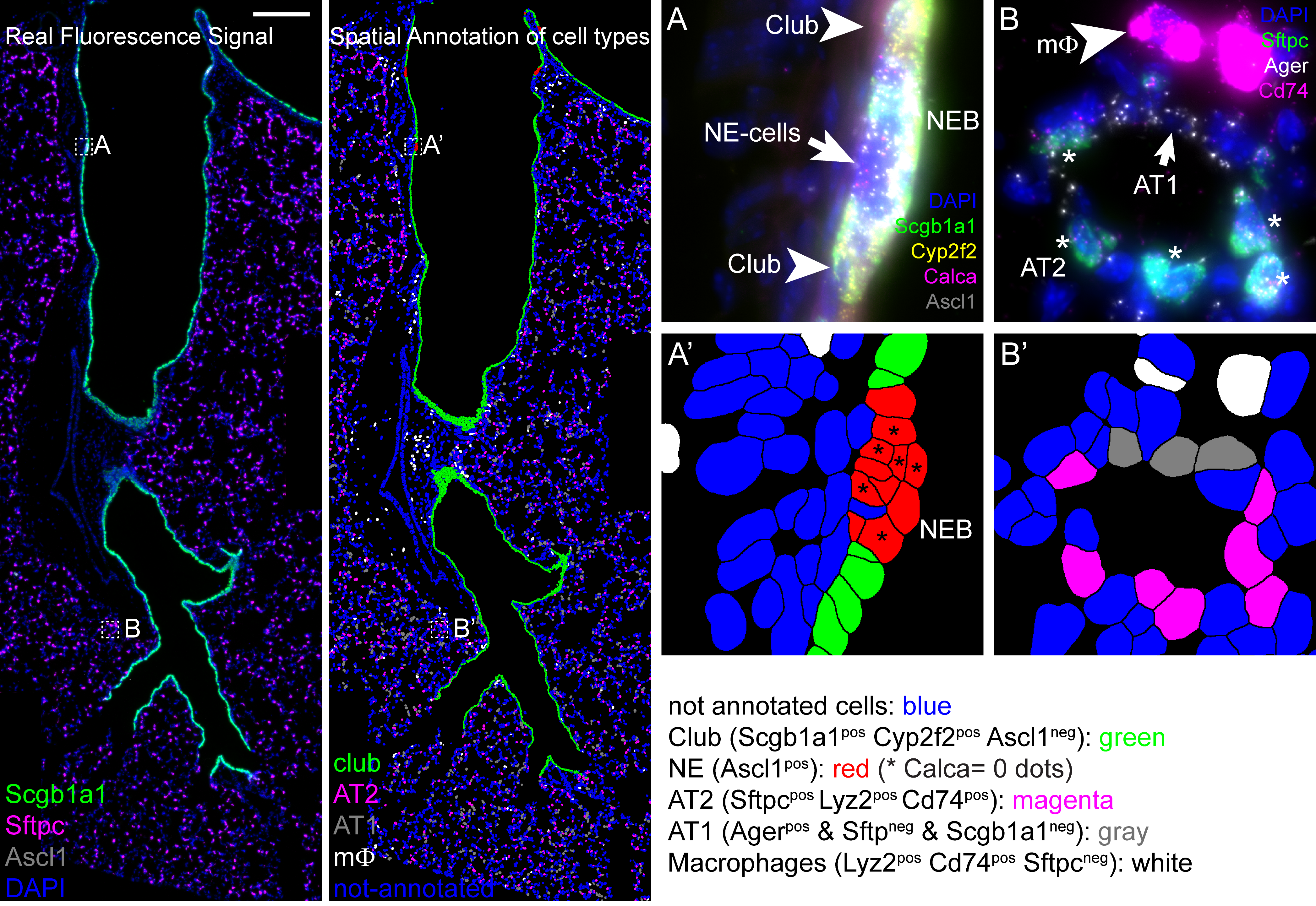

Supplement: S12 Fig — (1) Overview image of SCRINSHOT fluorescence signal dots for Scgb1a1 (green), Sftpc (magenta), and Ascl1 (gray) of a large area from P21 Sftpc-CreERpos;Rosa-Ai14pos lung section, after Tamoxifen induction on P1 (RFP was not shown at that image). The image contains 14,167 manual segmented nuclei, which were expanded for 2 μm and considered as cells. (2) Spatial map of annotated cell types according to the indicated criteria. (A) Same airway area as in Fig 6B, showing club (Scgb1a1: green and Cyp2f2: yellow) and NE-cell (Calca: magenta and Ascl1: gray) markers, in an airway position with a neuro-epithelial body (NEB). (A’) Cell-type digital annotation of the area corresponding to “A”. The “*” indicate Ascl1pos Calcaneg cells Club cells: green and NE-cells: red. (B) Same alveolar area as in Fig 6A showing Agerpos (gray) Sftpcneg (green) Cd74neg (magenta) AT1 cells (arrow), Agerlow Sftpcpos Cd74low AT2 cells (asterisks), and Agerneg Sftpcneg Lyz2pos Cd74high macrophages (mΦ, arrowhead). (B’) Cell-type digital annotation of the area corresponding to “B”. AT1 cells: gray, AT2 cells: magenta and macrophages: white. All not annotated cells are depicted with blue. Scale bar: 200 μm. The data underlying this figure can be found in 10.5281/zenodo.3634561. (TIF) [file pbio.3000675.s012.tif]
