# Supplementary material for: SCRINSHOT enables spatial mapping of cell states in tissue sections with single-cell resolution
Source: PLoS Biol. 2020 Nov 20;18(11):e3000675. doi: 10.1371/journal.pbio.3000675 (PMC7717588; doi:10.1371/journal.pbio.3000675)
Supplement: S2 Table — (PDF) [file pbio.3000675.s014.pdf]

Supporting Table 2

Detection Oligos

Name	mRNA recognized sequence (5' -->3')	sequence with Tm close to 56°C	Reverse Complement	GC content	Tm (^{o}C)	Detection Oligo
Axin2_m_pr1_SplR_Cy3	AGGCCTACGCAACAGCCACGAAATATTCTGAAGGAAAATGAAA	CGCAACAGCCACGAAATATTC	GAATATTTCGTGGCTGTTGCG	47.6	54.8	GAAUAUUUCGUGGCUGUUGCG[Cy3]
Axin2_m_pr2_SplR_Cy3	CCCAACACTTTGGCACAGCTAGAGGAAGCCTGCCGCAGGCT	GCACAGCTAGAGGAAGCC	GGCTTCCTCTAGCTGTGC	61.1	55.6	GGCUUCCUCUAGCUGUGC[Cy3]
Axin2_m_pr3_SplR_Cy3	ACGAAGCACGTTCACCACCACTACATCCACCACCACGCT	GTTCACCACCACTACATCCA	TGGATGTAGTGGTGGTGAAC	50	54.4	UGGAUGUAGUGGUGGUGAAC[Cy3]
Sftpc_m_pr7_SplR_Cy3	TGATACTGGTTCCGAGTCCGATTCTTCCGGGAGAGACCTG	CCGAGTCCGATTCTTCCG	CGGAAGAATCGGACTCGG	61.1	55.2	CGGAAGAAUCGGACUCGG[Cy3]
Sftpc_m_pr7_cDNA_Cy3	TGATACTGGTTCCGAGTCCGATTCTTCCGGGAGAGACCTG	CGGAAGAATCGGACTCGG	CCGAGTCCGATTCTTCCG	61.1	55.2	CCGAGUCCGAUUCUUCCG[Cy3]
Scgb1a1_pr7_SplR_FITC	AAGCAAGATTTAAGATTCTGAAGCTCACTGGATTTCAGAGATA	AAGATTCTGAAGCTCACTGGATTT	AAATCCAGTGAGCTTCAGAATCTT	37.5	54.5	AAAUCCAGUGAGCUUCAGAAUCUU[FITC]
Scgb1a1_pr7_cDNA_FITC	AAGCAAGATTTAAGATTCTGAAGCTCACTGGATTTCAGAGATA	AAATCCAGTGAGCTTCAGAATCTT	AAGATTCTGAAGCTCACTGGATTT	37.5	54.5	AAGAUUCUGAAGCUCACUGGAUUU[FITC]
Ascl1_m_pr1_SplR_Cy5	GAGGAACAAGAGCTGCTGGACTTTACCAACTGGTTCTGAG	AGCTGCTGGACTTTACCAAC	GTTGGTAAAGTCCAGCAGCT	50	55.2	GUUGGUAAAGUCCAGCAGCU[Cy5]
Ascl1_m_pr2_SplR_Cy5	CACACAGACAGACACTATATTAACTCCCAACCACTAACAG	ACAGACACTATATTAACTCCCAACCA	TGGTTGGGAGTTAATATAGTGTCTGT	38.5	55.4	UGGUUGGGAGUUAAUAUAGUGUCUGU[Cy5]
Ascl1_m_pr3_SplR_Cy5	CGATCTATGGACATTTGCCTCCATTGAAGCAACGTCAGTTC	GACATTTGCCTCCATTGAAGC	GCTTCAATGGAGGCAAATGTC	47.6	54.7	GCUUCAAUGGAGGCAAAUGUC[Cy5]
Cyp2f2_m_pr1_SplR_FITC	TCACCATCATCCACTTTATCAATGACAACTTCAAGATTATGAG	TCCACTTTATCAATGACAACTTCAAGAT	ATCTTGAAGTTGTCATTGATAAAGTGGA	32.1	54.9	AUCUUGAAGUUGUCAUUGAUAAAGUGGA[FITC]
Cyp2f2_m_pr2_SplR_FITC	CTCTGTGTCCAACATTATCTGCTCTGTCGTCTTCGGAAGTC	CAACATTATCTGCTCTGTCGTCT	AGACGACAGAGCAGATAATGTTG	43.5	54.7	AGACGACAGAGCAGAUAAUGUUG[FITC]
Cyp2f2_m_pr3_SplR_FITC	CACCACACTGCGTCACGCCTTCCTTATTCTTATGAAATAC	GCGTCACGCCTTCCTTAT	ATAAGGAAGGCGTGACGC	55.6	55.2	AUAAGGAAGGCGUGACGC[FITC]
Calca_m_pr1_SplR_FITC	CTTCTTGCTTTCTTCCTATAACTTGATGCATGTAGTTCCTC	GCTTTCTTCCTATAACTTGATGCATG	CATGCATCAAGTTATAGGAAGAAAGC	38.5	54.4	CAUGCAUCAAGUUAUAGGAAGAAAGC[Cy5]
Calca_m_pr2_SplR_FITC	CTGGGCACGTACACACAAGACCTCAACAAGTTTCACACCTTC	GTACACACAAGACCTCAACAAGT	ACTTGTTGAGGTCTTGTGTGTAC	43.5	55.0	ACUUGUUGAGGUCUUGUGUGUAC[Cy5]
Calca_m_pr3_SplR_FITC	CTATATGCAGATGAAAGCCAGGGAGCTGGAGCAGGAGGAAG	GCCAGGGAGCTGGAG	CTCCAGCTCCCTGGC	73.3	55.5	CUCCAGCUCCCUGGC[Cy5]
Etv5_m_pr5_SplR_Cy3	CTGGGAAGCAGATGTACATACTACATACATGTCACATACTAC	GGGAAGCAGATGTACATACTACATAC	GTATGTAGTATGTACATCTGCTTCCC	42.3	54.6	GUAUGUAGUAUGUACAUCUGCUUCCC[Cy3]
Etv5_m_pr6_SplR_Cy3	CCGAGTGTATGACAATCTGCGTTGATCATGGACTACTAAA	TGTATGACAATCTGCGTTGATCATG	CATGATCAACGCAGATTGTCATACA	40	55.5	CAUGAUCAACGCAGAUUGUUCAUACA[Cy3]
Etv5_m_pr7_SplR_Cy3	CCAAGAAACTACTCAATGCTGAAGCCCTTCAAAGTGATAG	ACTCAATGCTGAAGCCCTTC	GAAGGGCTTCAGCATTGAGT	50	55.3	GAAGGGCUUCAGCAUUGAGU[Cy3]
Fgfr2_m_pr1_SplR_Cy5	AATTCTGACTCTCACAACCAATGAGGAATACTTGGATCTC	ACTCTCACAACCAATGAGGAATACTTG	CAAGTATTCCTCATTGGTTGTGAGAGT	40.7	56.8	CAAGUAUUCCUCAUUGGUUGUGAGAGU[Cy5]
Fgfr2_m_pr2_SplR_Cy5	CTGGTATCAGAGATGGAGATGATGAAGATGATTGGGAAACATAAG	GAGATGGAGATGATGAAGATGATTGG	CCAATCATCTTCATCATCTCCATCTC	42.3	55.2	CCAAUCAUCUUCAUCAUCUCCAUCUC[Cy5]
Fgfr2_m_pr3_SplR_Cy5	CTTCAGTTTAGTTGAGGATACCACTTTAGAACCAGAAGGAG	TAGTTGAGGATACCACTTTAGAACCAG	CTGGTTCTAAAGTGGTATCCTCAACTA	40.7	55.8	CUGGUUCUAAAGUGGUAUCCUCAACUA[Cy5]
Spry2_m_pr1_SplR_Cy5	GAACAACAGAAGTTGGAGACCCACATTGCATAAGCTAAAG	AGTTGGAGACCCACATTGCATAA	TTATGCAATGTGGGTCTCCAACT	43.5	56.6	UUAUGCAAUGUGGGUCUCCAACU[Cy5]
Spry2_m_pr2_SplR_Cy5	GACGTTAAGCCACTGAGCAAGGATGATTTGGGTCTGCATG	GCCACTGAGCAAGGATGATTTG	CAAATCATCCTTGCTCAGTGGC	50	56.6	CAAAUCAUCCUUGCUCAGUGGC[Cy5]
Spry2_m_pr3_SplR_Cy5	GATTGCTTGGAAGTTGGACTGAAGCAGAGTTTGGAAAGAAG	GAAGTTGGACTGAAGCAGAGTTTG	CAAACTCTGCTTCAGTCCAACTTC	45.8	56.2	CAAACUCUGCUUCAGUCCAACUUC[Cy5]
RFP_Ai9_pr1_SplR_FITC	CTACTACTACGTGGACACCAAGCTGGACATCACCTCCCAC	GTGGACACCAAGCTGGAC	GTCCAGCTTGGTGTCCAC	61.1	56.1	GUCCAGCUUGGUGUCCAC[FITC]
RFP_Ai9_pr2_SplR_FITC	CTCCGAGGACAACAACATGGCCGTCATCAAAGAGTTCATG	CAACAACATGGCCGTCATCA	TGATGACGGCCATGTTGTTG	50	56.0	UGAUGACGGCCAUGUUGUUG [FITC]
Pecam1_m_pr1_SplR_Cy5	AAGTGACACACTTGGTCCAGGAGTTTACAGAAATTATCATC	ACTTGGTCCAGGAGTTTACAGA	TCTGTAAACTCCTGGACCAAGT	45.5	55.5	UCUGUAAACUCCUGGACCAAGU[Cy5]
Pecam1_m_pr2_SplR_Cy5	CATCACTTACCACCTTATGAAAGCAAAGAGTGACTTCCAG	ACCACCTTATGAAAGCAAAGAGTG	CACTCTTTGCTTTCATAAGGTGGT	41.7	55.5	CACUCUUUGCUUUCAUAAGGUGGU[Cy5]
Pecam1_m_pr4_SplR_Cy5	CATTACGGTTATGATGATGTTTCTGGAAATGATGCAGTAAA	GTTATGATGATGTTTCTGGAAATGATGCA	TGCATCATTTCCAGAAACATCATCATAAC	34.5	56.2	UGCAUCAUUUCCAGAAACAUCAUCAUAAC[Cy5]
Pecam1_m_pr1_SplR_FITC	AAGTGACACACTTGGTCCAGGAGTTTACAGAAATTATCATC	ACTTGGTCCAGGAGTTTACAGA	TCTGTAAACTCCTGGACCAAGT	45.5	55.5	UCUGUAAACUCCUGGACCAAGU[FITC]
Pecam1_m_pr2_SplR_FITC	CATCACTTACCACCTTATGAAAGCAAAGAGTGACTTCCAG	ACCACCTTATGAAAGCAAAGAGTG	CACTCTTTGCTTTCATAAGGTGGT	41.7	55.5	CACUCUUUGCUUUCAUAAGGUGGU[FITC]
Pecam1_m_pr4_SplR_FITC	CATTACGGTTATGATGATGTTTCTGGAAATGATGCAGTAAA	GTTATGATGATGTTTCTGGAAATGATGCA	TGCATCATTTCCAGAAACATCATCATAAC	34.5	56.2	UGCAUCAUUUCCAGAAACAUCAUCAUAAC[FITC]
Pecam1_m_pr1_cDNA_FITC	AAGTGACACACTTGGTCCAGGAGTTTACAGAAATTATCATC	TCTGTAAACTCCTGGACCAAGT	ACTTGGTCCAGGAGTTTACAGA	45.5	55.5	ACUUGGUCCAGGAGUUUACAGA[FITC]
Pecam1_m_pr2_cDNA_FITC	CATCACTTACCACCTTATGAAAGCAAAGAGTGACTTCCAG	CACTCTTTGCTTTCATAAGGTGGT	ACCACCTTATGAAAGCAAAGAGTG	41.7	55.5	ACCACCUUAUGAAAGCAAAGAGUG[FITC]
Pecam1_m_pr4_cDNA_FITC	CATTACGGTTATGATGATGTTTCTGGAAATGATGCAGTAAA	TGCATCATTTCCAGAAACATCATCATAAC	GTTATGATGATGTTTCTGGAAATGATGCA	34.5	56.2	GUUAUGAUGAUGUUUCUGGAAAUGAUGCA[FITC]
Ager_m_pr1_SplR_Cy5	TTCTGATTCCCGATGGCAAAGAAACACTCGTGAAGGAAGAGAC	CGATGGCAAAGAAACACTCG	CGAGTGTTTCTTTGCCATCG	50	54.4	CGAGUGUUUCUUUGCCAUCG[Cy5]
Ager_m_pr2_SplR_Cy5	CGAGTCTACCAGATTCCTGGGAAGCCAGAAATTGTGGATC	CAGATTCCTGGGAAGCCA	TGGCTTCCCAGGAATCTG	55.6	54.5	UGGCUUCCCAGGAAUCUG[Cy5]
Ager_m_pr3_SplR_Cy5	GAAGAGACCAGGAGACACCCTGAGACGGGACTCTTTACACTG	GACACCCTGAGACGGG	CCCGTCTCAGGGTGTC	68.8	54.9	CCCGUCUCAGGGUGUC[Cy5]
Cldn18_m_pr1_SplR_FITC	ATTATCTGAAATGGCATAAAGAATGAACAGACCAAACAATTTAAG	AATGGCATAAAGAATGAACAGACCAA	TTGGTCTGTTCATTCTTTATGCCATT	34.6	55.4	UUGGUCUGUUCAUUCUUUAUGCCAUU[FITC]
Cldn18_m_pr2_SplR_FITC	CTGAGTTATCGTTTATGAGTTAGAGGCCATAACACTCACTTTAG	GTTTATGAGTTAGAGGCCATAACACT	AGTGTTATGGCCTCTAACTCATAAAC	38.5	54.7	AGUGUUAUGGCCUCUAACUCAUAAAC[FITC]
Cldn18_m_pr3_SplR_FITC	CATCCCTTGACATTGTCTATAGCGTAAGATCCTATATAAATCCAA	TGACATTGTCTATAGCGTAAGATCCTAT	ATAGGATCTTACGCTATAGACAATGTCA	35.7	55.1	AUAGGAUCUUACGCUAUAGACAAUGUCA[FITC]
CD74_m_pr1_SplR_Cy3	CTCTTGTTTGAGATGAGCAAGAACTCCCTGGAGGAGAAGAAG	GATGAGCAAGAACTCCCTGG	CCAGGGAGTTCTTGCTCATC	55	55.4	CCAGGGAGUUCUUGCUCAUC[Cy3]
CD74_m_pr2_SplR_Cy3	CTTAACCTAGGCTGGACACATTTGCTGCCTTCTCCTCAAGGAAG	TGGACACATTTGCTGCCTT	AAGGCAGCAAATGTGTCCA	47.4	55.2	AAGGCAGCAAAUGUGUCCA[Cy3]
CD74_m_pr3_SplR_Cy3	CTCTCCTGTGCCTCAGCCCTTCTTATGTTCCCTGATGTCAC	GCCTCAGCCCTTCTTATGTT	AACATAAGAAGGGCTGAGGC	50	54.9	AACAUAAGAAGGGCUGAGGC[Cy3]
Lgi3_m_pr1_SplR_Cy5	CTCTAACAAGTTCACACTGATTGGAGACAATGCCTTCATAG	AGTTCACACTGATTGGAGACAATG	CATTGTCTCCAATCAGTGTGAACT	41.7	55.2	CAUUGUCUCCAAUCAGUGUGAACU[Cy5]
Lgi3_m_pr2_SplR_Cy5	GAACAATGACATCTGGGCTCTCTCCAAGTTTACCTTCAGAG	TCTGGGCTCTCTCCAAGT	ACTTGGAGAGAGCCCAGA	55.6	55.1	ACUUGGAGAGAGCCCAGA[Cy5]
Lgi3_m_pr3_SplR_Cy5	GAACCTTTCCTTTACTCTAGTGACCTCTACTTGGCTCTGG	CCTTTACTCTAGTGACCTCTACTTGG	CCAAGTAGAGGTCACTAGAGTAAAGG	46.2	55.8	CCAAGUAGAGGUCACUAGAGUAAAGG[Cy5]
Actb_m_pr1_SplR_Cy5	ATTGAACATGGCATTGTTACCAACTGGGACGACATGGAGAAGATC	CATTGTTACCAACTGGGACGA	TCGTCCCAGTTGGTAACAATG	47.6	55.0	UCGUCCCAGUUGGUAACAAUG[Cy5]
Actb_m_pr1_cDNA_Cy5	ATTGAACATGGCATTGTTACCAACTGGGACGACATGGAGAAGATC	TCGTCCCAGTTGGTAACAATG	CATTGTTACCAACTGGGACGA	47.6	55.0	CAUUGUUACCAACUGGGACGA[Cy5]
Napsa_m_pr1_SplR_Cy3	GACTATGTCATCAAGATTCTTCAGAGCGATGTTGGCCTCTG	AAGATTCTTCAGAGCGATGTTGG	CCAACATCGCTCTGAAGAATCTT	43.5	55.2	CCAACAUCGCUCUGAAGAAUCUU[Cy3]
Napsa_m_pr2_SplR_Cy3	CTTGGACACAGAATCTTAAACCCACTGAATGGATGGGAAC	ACAGAATCTTAAACCCACTGAATGG	CCATTCAGTGGGTTTAAGATTCTGT	0.4	55.2	CCAUUCAGUGGGUUUAAGAUCUGU[Cy3]
Napsa_m_pr3_SplR_Cy3	AGATGTCATTTCTTCAGTTTGGCATGCTGGTTTCACCATC	TTCAGTTTGGCATGCTGGT	ACCAGCATGCCAAACTGAA	47.4	55.2	ACCAGCAUGCCAAACUGAA[Cy3]
Lyz2_m_pr1_SplR_Cy5	GAGACCAAAGCACTGACTATGGGATATTTCAGATCAATAG	CAAAGCACTGACTATGGGATATTTCA	TGAAATATCCCATAGTCAGTGCTTTG	38.5	55.1	UGAAAUAUCCCAUAGUCAGUGCUUUG[Cy5]
Lyz2_m_pr2_SplR_Cy5	CAAGGTCTATGAACGTTGTGAGTTTGCCAGAACTCTGAAA	GAACGTTGTGAGTTTGCCAG	CTGGCAAACTCACAACGTTC	50	54.6	CUGGCAAACUCACAACGUUC[Cy5]
Lyz2_m_pr3_SplR_Cy5	GTGTGTTTAGCTCAGCACGAGAGCAATTATAACACACGAG	GCTCAGCACGAGAGCAATTATAA	TTATAATTGCTCTCGTGCTGAGC	43.5	55.2	UUAUAAUUGCUCUCGUGCUGAGC[Cy5]
padlock_backbone_A2_FITC	CAGCGTCACGCAAGATATAC	CAGCGTCACGCAAGATATAC	GTATATCTTGCGTGACGCTG	50	53.5	GUAUAUCUUGCGUGACGCUG[FITC]
Scgb3a1_m_pr1_SplR_FITC	CTAGCCTACCATTAAGCCAC TTGGCCATCCTGAGGTTCATC	ATTAAGCCAC TTGGCCATCC	GGATGGCCAAGTGGCTTAAT	50	55.5	GGAUGGCCAAGUGGCUUAAU[FITC]
Scgb3a1_m_pr2_SplR_FITC	GATCCTCTCATAGAGGGATCC AGGAAGTGTGTCACCGAGCTG	TAGAGGGATCC AGGAAGTGT	ACACTTCCTGGATCCCTCTA	50	54.5	ACACUUCCUGGAUCCCUCUA[FITC]
Scgb3a1_m_pr3_SplR_FITC	GAGACTCATTCTACCATGAA GCTTACCACCACCTTTCTA	CTACCATGAA GCTTACCACCA	TGGTGGTAAGCTTCATGGTAG	47.6	54.3	UGGUGGUAAGCUUCAUGGUAG[FITC]
Bpifb1_m_pr1_SplR_FITC	GAAGACAACCGGCTTATGCT CAACTTCAATAACGTCAGTATT	CCGGCTTATGCT CAACTTCAATA	TATTGAAGTTGAGCATAAGCCGG	43.5	55.3	UAUUGAAGUUGAGCAUAAGCCGG[FITC]
Bpifb1_m_pr2_SplR_FITC	CTGAGATACATCATATGGATG AAGGTCACCTCTGCTAACATC	TCATATGGATG AAGGTCACCTCT	AGAGGTGACCTTCATCCATATGA	43.5	55.1	AGAGGUGACCUUCAUCCAUAUGA[FITC]
Bpifb1_m_pr3_SplR_FITC	CAGTAATATCCTGTTGAACC TGAAGGCCAAGCTGCTGGAC	CTGTTGAACC TGAAGGCCAA	TTGGCCTTCAGGTTCAACAG	0.5	55.5	UUGGCCUUCAGGUUCAACAG[FITC]
Tfcp2l1_m_pr1_SplR_Cy5	GTGATAAGATACTCTGACAA AGGCAACTTGATAAAGAAAG	ATACTCTGACAA AGGCAACTTGAT	ATCAAGTTGCCTTTGTCAGAGTAT	37.5	54.5	AUCAAGUUGCCUUUGUCAGAGUAU[Cy5]
Tfcp2l1_m_pr2_SplR_Cy5	CAGTAGCTATGGTAGACTT AATTCATCTTTATTATGCCCA	AGCTATGGTAGACTT AATTCATCTTTATTATGC	GCATAATAAAGATGAATTAAGTCTACCATAGCT	50.3	55.3	GCAUAAUAAAGAUGAAUUAAGUCUACCAUAGCU[Cy5]
Tfcp2l1_m_pr3_SplR_Cy5	AACATGATTAAAGTTTAGTTG TTACAATGAGTTCTGCTC	TGATTAAAGTTTAGTTG TTACAATGAGTTCTG	CAGAACTCATTGTAACAACTAAACTTTAATCA	28.1	54.5	CAGAACUCAUUGUAACAACUAAACUUUAAUCA[Cy5]
Reg3g_m_pr1_SplR_FITC	CTATGGCTCCTATTGCTAT GCCTTGTTTAGTGTATCTAAA	TCCTATTGCTAT GCCTTGTTTAGTG	CACTAAACAAGGCATAGCAATAGGA	40	55.0	CACUAAACAAGGCAUAGCAAUAGGA[FITC]
Reg3g_m_pr2_SplR_FITC	CAGTGGTAACAGTGGCCAAT ATGTATGGATTGGGCTCCATG	CAGTGGCCAAT ATGTATGGATTGG	CCAATCCATACATATTGGCCACTG	45.8	55.9	CCAAUCCAUACAUAUUGGCCACUG[FITC]
Reg3g_m_pr3_SplR_FITC	AGGATTTCTGAAGTGGAGA GAGAATTATTGTAACTTAGAGTT	TCTGAAGTGGAGA GAGAATTATTGTAAC	GTTACAATAATTCTCTCTCCACTTCAGA	35.7	54.4	GUUACAAUAAUUCUCUCUCCACUUCAGA[FITC]
Muc5b_m_pr1_SplR_FITC	GCTTTAACTACAATATACGTGT GCTTTGCTGTGATGACTATA	CAATATACGTGT GCTTTGCTGTG	CACAGCAAAGCACACGTATATTG	43.5	54.7	CACAGCAAAGCACACGUAUAUUG[FITC]
Muc5b_m_pr2_SplR_FITC	CATGACATCTACCCTACCAG TAACTGTGACTAATGCTACTAC	CTACCCTACCAG TAACTGTGACT	AGTCACAGTTACTGGTAGGGTAG	47.8	55.3	AGUCACAGUUACUGGUAGGGUAG[FITC]
Muc5b_m_pr3_SplR_FITC	CATTCACTTCAAACAGCAGA CTCACTTCAACTTCTATGTC	CAAACAGCAGA CTCACTTCAAC	GTTGAAGTGAGTCTGCTGTTTG	45.5	54.3	GUUGAAGUGAGUCUGCUGUUUG[FITC]
Tff2_m_pr1_SplR_FITC	CTGATCTTTGAAGTGCCCTG GTGTTTCTTCCCACAGTCTG	AAGTGCCCTG GTGTTTCTTC	GAAGAAACACCAGGGCACTT	50	55.5	GAAGAAACACCAGGGCACUU[FITC]
Tff2_m_pr2_SplR_FITC	GTGAGCAGTGCTTTGATCTT GGATGCTGCTTTGACTCTAG	GCTTTGATCTT GGATGCTGCT	AGCAGCATCCAAGATCAAAGC	47.6	55.9	AGCAGCAUCCAAGAUCAAAGC[FITC]
Tff2_m_pr3_SplR_FITC	CAAGCCAGAAGAAAGTTTCAA CCAGACTTCATCAATCTCTG	GAAGAAAGTTTCAA CCAGACTTCATC	GATGAAGTCTGGTTGAAACTTTCTTC	38.5	54.4	GAUGAAGUCUGGUUGAAACUUUCUUC[FITC]
Pax9_m_pr1_SplR_Cy3	TATGTTAATGTTATGAACAGAG CTATAGGACATGCAATGTGT	GTTATGAACAGAG CTATAGGACATGC	GCATGTCCTATAGCTCTGTTCATAAC	42.3	55.2	GCAUGUCCUAUAGCUCUGUUCAUAAC[Cy3]
Pax9_m_pr2_SplR_Cy3	ACCACACCTGTATATTTATTC TAAATCAACCTGAACTTTCGA	CACCTGTATATTTATTC TAAATCAACCTGAAC	GTTCAGGTTGATTTAGAATAAATATACAGGTG	31.2	54.5	GUUCAGGUUGAUUUAGAAUAAAUAUACAGGUG[Cy3]
Pax9_m_pr3_SplR_Cy3	TTTCATTCTCGCTCCTCTC TCCAGGTCTCTAATTGATAAT	GCTCCTCTC TCCAGGTCT	AGACCTGGAGAGAGGAGC	61.1	55.5	AGACCUGGAGAGAGGAGC[Cy3]
Six1_m_pr1_SplR_Cy3	AAACAATAACTCCTCCTCCAA CAAGCAGAATCAACTCTCTC	CCTCCTCCAA CAAGCAGAAT	ATTCTGCTTGTTGGAGGAGG	50	54.8	AUUCUGCUUGUUGGAGGAGG[Cy3]
Six1_m_pr2_SplR_Cy3	ACAGAGAGAATAGTGGCTA GGTTAATGATATGACGAAAGAAA	GAGAATAGTGGCTA GGTTAATGATATGAC	GTCATATCATTAACCTAGCCACTATTCTC	37.9	54.8	GUCAUAUCAUUAACCUAGCCACUAUUCUC[Cy3]
Six1_m_pr3_SplR_Cy3	CATCCTCTCCCTCCTCTC CAACTCTTAATACCCTTGCATTTC	CTCCCTCCTCTC CAACTCTTAATA	TATTAAGAGTTGGAGAGGAGGGAG	45.8	55.0	UAUUAAGAGUUGGAGAGGAGGGAG[Cy3]
Lgr6_m_pr1_SplR_Cy5	AATACTTCTGACAGAGGCCT GGGAAATTTGCATAGGAGAAAG	ACAGAGGCCT GGGAAATTTG	CAAATTTCCCAGGCCTCTGT	50	55.4	CAAAUUCCCAGGCCUCUGU[Cy5]
Lgr6_m_pr2_SplR_Cy5	ATTAATCTTGACCTATGCTGTG CCAAAGTGCTTCCTGTTAAA	CTATGCTGTG CCAAAGTGCT	AGCACTTTGGCACAGCATAG	50	55.8	AGCACUUUGGCACAGCAUAG[Cy5]
Lgr6_m_pr3_SplR_Cy5	CTTTCTCAGATGTGGATCTTA TTCTGGAAGCTTCTGAGGC	GATGTGGATCTTA TTCTGGAAGCTTC	GAAGCTTCCAGAATAAGATCCACATC	42.3	55.5	GAAGCUCCAGAAUAAGAUCCACAUC[Cy5]
Il13ra1_m_pr1_SplR_Cy5	GTAATCTCCAGGCCTTAGGG ACTCATAAAGATATGAGTACATTG	AGGCCTTAGGGACTCATAAAGATATG	CATATCTTTATGAGTCCCTAAGGCCT	42.3	55.8	CAUAUCUUUAUGAGUCCCUAAGGCCU[Cy5]
Il13ra1_m_pr2_SplR_Cy5	GATATTGCAGTTTCTCCTGCT ATTCACTAGAAAGAAGGCTTG	CAGTTTCTCCTGCTATTCACTAGAAAG	CTTTCTAGTGAATAGCAGGAGAAACTG	40.7	55.2	CUUUCUAGUGAAUAGCAGGAGAAACUG[Cy5]
Il13ra1_m_pr3_SplR_Cy5	GGCTTAAGATCATTATATTTCC TCCAATTCCTGATCCTGG	AAGATCATTATATTTCCTCCAATTCCTGATC	GATCAGGAATTGGAGGAAATATAATGATCTT	32.3	55.1	GAUCAGGAAUUGGAGGAAAUAUAAUGAUCUU[Cy5]
Pdpn_m_pr1_SplR_Cy5	ATTCCAGCCCAGTCCTAAGCA TCCACTGTGCCTTCAGTTCCGGGA	CTAAGCATCCACTGTGCCTT	AAGGCACAGTGGATGCTTAG	50	55.1	AAGGCACAGUGGAUGCUUAG[Cy5]
Pdpn_m_pr2_SplR_Cy5	ATCCTCTTCCTGTGGAGGAAGA TGACCCATGGCGTGCCCACCCA	GGAGGAAGATGACCCATGG	CCATGGGTCATCTTCCTCC	57.9	54.9	CCAUGGGUCAUCUUCCUCC[Cy5]
Pdpn_m_pr3_SplR_Cy5	TGGCAAGGCACCTCTGGTACCA ACGCAGAGAGAGCGTGGGACGAA	TCTGGTACCAACGCAGAGA	TCTCTGCGTTGGTACCAGA	52.6	55.6	UCUCUGCGUUGGUACCAGA[Cy5]
Krt5_m_pr1_SplR_FITC	GAGAATGAGTTTGTGATGTTG AAGAAGGATGTGGATGCTG	AGTTTGTGATGTTGAAGAAGGATGTG	CACATCCTTCTTCAACATCACAAACT	38.5	55.8	CACAUCCUUCUUCAACAUCACAAACU[FITC]
Krt5_m_pr2_SplR_FITC	GATCCGTTGTTTGAACAGTAC ATTAACAACCTCCGTAGACAG	TTGTTTGAACAGTACATTAACAACCTCC	GGAGGTTGTTAATGTACTGTTCAAACAA	35.7	55.9	GGAGGUUGUUAAUGUACUGUUCAAACAA[FITC]
Krt5_m_pr3_SplR_FITC	CATTTGTGTTGCACGTCAG CACCTATACATGTTCTTTGGTG	GCACGTCAGCACCTATACATG	CATGTATAGGTGCTGACGTGC	52.4	55.8	CAUGUAUAGGUGCUGACGUGC[FITC]
Trp63_m_pr1_SplR_Cy3	CAGAATACACACGGAATCCA GATGACTTCCATCAAGAAAC	CACGGAATCCAGATGACTTCC	GGAAGTCATCTGGATTCCGTG	52.4	55.4	GGAAGUCAUCUGGAUUCCGUG[Cy3]
Trp63_m_pr2_SplR_Cy3	CACTGAATTCACAACAGTCCTGTA CAATTTCATGTGTAACAGCAG	ACAACAGTCCTGTACAATTTCATGTG	CACATGAAATTGTACAGGACTGTTGT	38.5	55.7	CACAUGAAAUUGUACAGGACUGUUGU[Cy3]
Trp63_m_pr3_SplR_Cy3	CCACCTGGACGTATTCCAC CGAACTGAAGAAGCTGTACTG	CGTATTCCACCGAACTGAAGAAG	CTTCTTCAGTTCGGTGGAATACG	47.8	55.5	CUUCUUCAGUUCGGUGGAAUACG[CY3]
Foxq1_m_pr1_SplR_Cy3	CTCATTCCTCGCTTGTCTTC CGCTCAAAGTCTCTAAACCTC	CTTGTCTTCCGCTCAAAGTCT	AGACTTTGAGCGGAAGACAAG	47.6	54.9	AGACUUUGAGCGGAAGACAAG[Cy3]
Foxq1_m_pr2_SplR_Cy3	CTTTCCTTTCCCTCCTATTTGCG TCACGCCTTCCACTGATTTAC	TCCTATTTGCGTCACGCC	GGCGTGACGCAAATAGGA	55.6	55.5	GGCGUGACGCAAAUAGGA[Cy3]
Foxq1_m_pr3_SplR_Cy3	CTGAATTGAAATGTTGCAGGC AACGGCTACAGCTTTATTAG	GTTGCAGGCAACGGC	GCCGTTGCCTGCAAC	66.7	55.3	GCCGUUGCCUGCAAC[Cy3]
Gp2_m_pr1_SplR_Cy3	GTGGAGATAATGAGATCAAGG TGAAGTTGGACAAGTGTTTG	TGAGATCAAGGTGAAGTTGGACA	TGTCCAACTTCACCTTGATCTCA	43.5	56	UGUCCAACUUCACCUUGAUCUCA[Cy3]
Gp2_m_pr2_SplR_Cy3	CCTGGAAATGCAATGTATATTG ATAACTCCTGGGTAGAGTTG	TGCAATGTATATTGATAACTCCTGGG	CCCAGGAGTTATCAATATACATTGCA	38.5	54.8	CCCAGGAGUUAUCAAUAUACAUUGCA[Cy3]
Gp2_m_pr3_SplR_Cy3	CAGTGGAGTCTATACTCTATG TGGGTGTCCTCTTAAATAGAG	TCTATACTCTATGTGGGTGTCCTCT	AGAGGACACCCACATAGAGTATAGA	44	55.9	AGAGGACACCCACAUAGAGUAUAGA[Cy3]
Spdef_m_pr1_SplR_Cy3	GTATTACAAGAAGGGCATCAT TCGTAAACCCGACATCTCTCAG	AAGGGCATCATTCGTAAACCC	GGGTTTACGAATGATGCCCTT	47.6	55.5	GGGUUUACGAAUGAUGCCCUU[Cy3]
Spdef_m_pr2_SplR_Cy3	CAGGAAGATCGAGGGAGCTA GGATTGTTCACAAACGGAAC	GAGGGAGCTAGGATTGTTCAC	GTGAACAATCCTAGCTCCCTC	52.4	54.9	GUGAACAAUCCUAGCUCCCUC[Cy3]
Spdef_m_pr3_SplR_Cy3	CTGAGCAGGGCCTGTCTG CTTTCTACCTCTCTTACTTTAAC	CCTGTCTGCTTTCTACCTCTCTT	AAGAGAGGTAGAAAGCAGACAGG	47.8	56	AAGAGAGGUAGAAAGCAGACAGG[Cy3]
Lipf_m_pr1_SplR_Cy3	CTAAATATGACCTTCCAGCC ACCATAGACTTCATTGTACAG	CTTCCAGCCACCATAGACTTC	GAAGTCTATGGTGGCTGGAAG	52.4	55.1	GAAGUCUAUGGUGGCUGGAAG[Cy3]
Lipf_m_pr2_SplR_Cy3	GAGGAATATGAAGTTGTTACT GAAGATGGCTACATTCTGG	ATGAAGTTGTTACTGAAGATGGCTAC	GTAGCCATCTTCAGTAACAACTTCAT	38.5	55	GUAGCCAUCUUCAGUAACAACUUCAU[Cy3]
Lipf_m_pr3_SplR_Cy3	CTTAAATGTGAGTCGCTTTGA TGTGTATCTAGGGCATAATCC	GAGTCGCTTTGATGTGTATCTAGG	CCTAGATACACATCAAAGCGACTC	45.8	55	CCUAGAUACACAUCAAAGCGACUC[Cy3]
Dcpp3_m_pr1_SplR_Cy5	GAATTCAGTTCAACTATGATGAC AAGGACGGTCAAGTGTATG	TCAACTATGATGACAAGGACGGT	ACCGTCCTTGTCATCATAGTTGA	43.5	55.8	ACCGUCCUUGUCAUCAUAGUUGA[Cy5]
Dcpp1_m_pr1_SplR_Cy5	CACATACACTCCTTCGGCTT TAACTCAGATAATCTTTACGAC	ACTCCTTCGGCTTTAACTCAGATAAT	ATTATCTGAGTTAAAGCCGAAGGAGT	38.5	55.8	AUUAUCUGAGUUAAAGCCGAAGGAGU[Cy5]
Dcpp1_m_pr2_SplR_Cy5	CCGGGTATTTCTTAAGGGT AGATTGATTGTTGGAATTCAG	GTATTTCTTAAGGGTAGATTGATTGTTGGAAT	ATTCCAACAATCAATCTACCCTTAAGAAATAC	31.2	55.6	AUUCCAACAAUCAAUCUACCCUUAAGAAAUAC[Cy5]
Dcpp1_m_pr3_SplR_Cy5	CGTTAGGGAAGCTAGAGTAC TCAGAATTGCCCACATAATAC	GCTAGAGTACTCAGAATTGCCC	GGGCAATTCTGAGTACTCTAGC	50	54.9	GGGCAAUUCUGAGUACUCUAGC[Cy5]
Foxi1_m_pr1_SplR_Cy3	GAAGCTTGGAAGAATCTGTG CTCTTTCTAAACAGCATTCAAG	GAATCTGTGCTCTTTCTAAACAGCAT	ATGCTGTTTAGAAAGAGCACAGATTC	38.5	55.6	AUGCUGUUUAGAAAGAGCACAGAUUC[Cy3]
Foxi1_m_pr2_SplR_Cy3	CTTCCGAGAGAAACAAGGT CAAATGAGCAGAATAAGCATG	GAGAAACAAGGTCAAATGAGCAGAATA	TATTCTGCTCATTTGACCTTGTTTCTC	37	55.5	UAUUCUGCUCAUUUGACCUUGUUUCUC[Cy3]
Foxi1_m_pr3_SplR_Cy3	CACTGAAGTCTAGAGATAGA ACAACTTCTGTTCCATAGAGC	TCTAGAGATAGAACAACTTCTGTTCCAT	ATGGAACAGAAGTTGTTCTATCTCTAGA	35.7	55.2	AUGGAACAGAAGUUGUUCUAUCUCUAGA[Cy3]
Cftr_m_pr1_SplR_Cy5	GAAAGTTGCAGATGAGGTTG GACTCAAGTCTGTAATAGAG	AGATGAGGTTGGACTCAAGTCT	AGACTTGAGTCCAACCTCATCT	45.5	55.3	AGACUUGAGUCCAACCUCAUCU[Cy5]
Cftr_m_pr2_SplR_Cy5	CTATAATAGTCGTCTCGGCAT TACAACCCTACATCTTCCTAG	CGTCTCGGCATTACAACCC	GGGTTGTAATGCCGAGACG	57.9	56.0	GGGUUGUAAUGCCGAGACG[Cy5]
Cftr_m_pr3_SplR_Cy5	ACATTCCAGTGATGAGAACA ATGTCAGTTTCAGTCATCTC	AGTGATGAGAACAATGTCAGTTTCAG	CTGAAACTGACATTGTTCTCATCACT	38.5	55.3	CUGAAACUGACAUUGUUCUCAUCACU[Cy5]
Ascl3_m_pr1_SplR_FITC	CTGCCATCAAATATATCAGCT ACCTGCAGTCTCTCTTGTAC	AAATATATCAGCTACCTGCAGTCTCT	AGAGACTGCAGGTAGCTGATATATTT	38.5	55.3	AGAGACUGCAGGUAGCUGAUAUAUUU[FITC]
Ascl3_m_pr2_SplR_FITC	CAGTACATTCTCATACACATG GTTTCGAGGACACAACAGTAC	TCATACACATGGTTTCGAGGACA	TGTCCTCGAAACCATGTGTATGA	43.5	56.0	UGUCCUCGAAACCAUGUGUAUGA[FITC]
Ascl3_m_pr3_SplR_FITC	CATGCCTTACACCAACTACAG GCGCTGTGACTACACCTAC	ACCAACTACAGGCGCTG	CAGCGCCTGTAGTTGGT	58.8	55.1	CAGCGCCUGUAGUUGGU[FITC]
Gng13_m_pr1_SplR_FITC	CCACACTCTCTGTAAGGTG TGACTTTATAAATAGACTTCTCC	TCTCTGTAAGGTGTGACTTTATAAATAGACT	AGTCTATTTATAAAGTCACACCTTACAGAGA	32.3	55.3	AGUCUAUUUAUAAAGUCACACCUUACAGAGA[FITC]

Gng13_m_pr2_SplR_FITC	CAGATGAAGAAGGAGGTAGA GAGCCTCAAGTACCAACTGG	AAGGAGGTAGAGAGCCTCAAG	CTTGAGGCTCTCTACCTCCTT	52.4	55.6	CTTGAGGCUCTCTACCTCCTU[FITC]
Gng13_m_pr3_SplR_FITC	CTTCCTGAACCCAGACCTGA TGAAGAACAACCCTTGGGTA	ACCCAGACCTGATGAAGAACA	TGTTCTTCATCAGGTCTGGGT	47.6	55.8	TGTTCTUCATCAGGTCTGGGU[FITC]
Trpm5_m_pr1_SplR_Cy3	CTACAGATCACTTCTTGGACA TCCCTTCCTAAGAGAATGAAACTC	TTCTTGGACATCCCTTCCTAAGA	TCTTAGGAAGGGATGTCCAAGAA	43.5	55.2	TCTTAGGAAGGGATGUCCAAGAA[Cy3]
Trpm5_m_pr2_SplR_Cy3	CTATTCACAAGCAGTTGGGT CCTAAGATCATCATTGTAGAG	GCAGTTGGGTCCTAAGATCATC	GATGATCTTAGGACCCAACTGC	50	55.2	GATGAUCTTAGGACCCAACUGC[Cy3]
Trpm5_m_pr3_SplR_Cy3	CTTTCTATAAAGCCTGTAGAC CACCTCAAATCAGCTAAAC	TAAAGCCTGTAGACCACCTCAAAT	ATTTGAGGTGGTCTACAGGCTTTA	41.7	55.9	ATTTGAGGUGGTCTACAGGCTTUA[Cy3]
Alox5ap_m_pr1_SplR_Cy5	GATACTCAACCATTACCTCATCT TCTTCTTCGGAAGCGACTTTG	CATTACCTCATCTTCTTCTTCGGAAG	CTTCCGAAGAAGAAGATGAGGTAATG	42.3	55.2	TGAGTAGAAAUGCCACAACAGC[Cy5]
Alox5ap_m_pr2_SplR_Cy5	CTTATCCTGCTTTCTGAAGAT GTCTTGTAATCAGACACGTG	TGCTTTCTGAAGATGTCTTGTAATCAG	CTGATTACAAGACATCTTCAGAAAGCA	37	55.3	CCTGUCCAACTCATAGCUCC[Cy5]
Alox5ap_m_pr3_SplR_Cy5	CAGAGAAGGACGCTCACCA GATCAATAGAGACGCATCATAAC	CGCTCACCAGATCAATAGAGAC	GTCTCTATTGATCTGGTGAGCG	50	54.9	TCTGGUTGTGGTAAAGUGGGA[Cy5]
Sox9_m_pr1_SplR_Cy5	ATGATAAAGCTCACCAATGCT CTATGTTATCTGTTTATTCCC	GCTCACCAATGCTCTATGTTATCTG	CAGATAACATAGAGCATTGGTGAGC	44	55.6	CAGAUAACAUAGAGCATTGGUGAGC[Cy5]
Sox9_m_pr2_SplR_Cy5	CATCTCCAACATTGAGACCT TCGACGTCAATGAGTTTGAC	ACATTGAGACCTTCGACGTCA	TGACGTCGAAGGTCTCAATGT	47.6	56	TGACGUCGAAGGUCTCAAUGT[Cy5]
Il25_m_pr1_SplR_Cy5	CTAGAGGATGCTGTTGTGGCA TTTCTACTCAGGAACGGACTC	GCTGTTGTGGCATTTCTACTCA	TGAGTAGAAATGCCACAACAGC	45.5	55.5	TGAGTAGAAAUGCCACAACAGC[Cy5]
Il25_m_pr2_SplR_Cy5	CATCTCTCCTTGGAGCTATG AGTTGGACAGGGACTTGAATC	GGAGCTATGAGTTGGACAGG	CCTGTCCAACTCATAGCTCC	55	54.8	CCTGUCCAACTCATAGCUCC[Cy5]
Il25_m_pr3_SplR_Cy5	CAACTCCGTCCCACTTTACC ACAACCAGACGGTCTTCTAC	TCCCACTTTACCACAACCAGA	TCTGGTTGTGGTAAAGTGGGA	47.6	56.0	TCTGGUTGTGGTAAAGUGGGA[Cy5]
SOX2_h_pr1_Cy3	GAAAGTAGTTTGCTGCCTCTTTAAGACTAGGACTGAGAGAAAG	TGCTGCCTCTTTAAGACTAGGAC	GTCCTAGTCTTAAAGAGGCAGCA	47.8	56.2	GTCCUAGTCUUAAAGAGGCAGCA[Cy3]
SOX2_h_pr2_Cy3	GAATGGACCTTGTATAGATCTGGAGGAAAGAAAGCTACGAAA	CTTGTATAGATCTGGAGGAAAGAAAGC	GCTTTCTTTCCTCCAGATCTATACAAG	40.7	55.1	GCTTTCUTTCCTCCAGAUCTAUACAAG[Cy3]
SOX2_h_pr3_Cy3	GTCTTTACCAATAATATTTAGAGCTAGTCTCCAAGCGACG	CCAATAATATTTAGAGCTAGTCTCCAAGC	GCTTGGAGACTAGCTCTAAATATTATTGG	37.9	55.2	GCTTGGAGACUAGCTCTAAAUATTATUGG [Cy3]
SOX9_h_pr1_FITC	GGATCAGCCCACTGACAGACCTTAATCTTAATTACTGCTG	GCCCACTGACAGACCTTAATC	GATTAAGGTCTGTCAGTGGGC	52.4	55.4	GATUAAGGTCUGTCAGUGGGC[FITC]
SOX9_h_pr2_FITC	CAAATTTCCAAGACACAAACATGACCTATCCAAGCGCATTAC	CAAGACACAAACATGACCTATCCAAG	CTTGGATAGGTCATGTTTGTGTCTTG	42.3	55.9	CTTGGAUAGGTCATGUTTGTGUCTTG[FITC]
SOX9_h_pr3_FITC	TTTAAACCCTCTTCAGAGCAAGCGTGGAGGATGATGGAGAATC	TTCAGAGCAAGCGTGGAG	CTCCACGCTTGCTCTGAA	56.6	55.1	CTCCACGCUTGCTCUGAA[FITC]
ASCL1_h_pr1_Cy5	TCAGCACCAATGTGTCTTACTTTATAGAAATGTTGTTAATGT	CCAATGTGTCTTACTTTATAGAAATGTTGTT	AACAACATTTCTATAAAGTAAGACACATTGG	29	54.5	AACAACATUTCTATAAAGUAAGACACATUGG[Cy5]
ASCL1_h_pr2_Cy5	ATAAGCTGTAGACATTGGTTTACAGTGAAACTATGCTATTCT	TGTAGACATTGGTTTACAGTGAAACTATG	CATAGTTTCACTGTAAACCAATGTCTACA	34.5	55.2	CATAGUTTCACTGUAAACCAATGTCUACA[Cy5]

Primers-Oligos

Name	Sequence	Information
RCA_primer1	TAAATAGACGCAGTCAGT*A*A	"*" indicates Thiophospate modified bond
RCA_primer2	CGCAAGATATACG*T*C	"*" indicates Thiophospate modified bond
Oligo-dT30 VN	*AAGCAGTGGTATCAACGCAGAGTACTTTTTTTTTTTTTTTTTTTTTTTTTTTTTTVN*	
Scgb1a1_competitor	AGCAGTAGAATATCTCTGAAATCCAGTGAGCTTCAGAATCTTAAATCTTGCTTACACAGAGGA	
Scambled_competitor	GAGTACACTAATATAAGACTCCTACGGCGCTCGATTCTATAACATAGATAGTAATGGTACATG	
