# Supplementary material for: SCRINSHOT enables spatial mapping of cell states in tissue sections with single-cell resolution
Source: PLoS Biol. 2020 Nov 20;18(11):e3000675. doi: 10.1371/journal.pbio.3000675 (PMC7717588; doi:10.1371/journal.pbio.3000675)
Supplement: S1 Text — (PDF) [file pbio.3000675.s016.pdf]

Step-by-step SCRINSHOT protocol

Table of contents

1. Probe preparation 3
1.1 Padlock probe design 3
1.1.1 Selecting specific sequences for the padlock probe gene-specific arms 4
1.1.2 Preparation of the Padlock Probes with the "Padlock Design Assistant" 5
1.1.3 Padlock probe documentation 5
1.1.4 Ordering of padlock probes 5
1.2 Detection-oligo design 6
2. Tissue preparation 7
2.1 Example of tissue collection and fixation: newborn and adult mouse lung 9
2.2 Tissue Freezing 9
2.3 Sectioning 9
3. SCRINSHOT hybridization protocol 10
3.1 Post-fixation of the slides 11
3.2 Permeabilization of the tissue 11
3.3 Mounting of hybridization chambers 11
3.4 Blocking 12
3.5 Hybridization of Padlock probes 12
3.6 Ligation of Padlock probes 13
3.7 Rolling circle amplification (RCA) 14
3.8 Hybridization of the first set of detection oligos 15
3.9 Removal of imaged detection oligos, prior to the next hybridization 15
4. Imaging 16
5. Image preparation for visualization and analysis 17
5.1 Orthogonal projection and stitching 17
5.2 Image-dataset alignment of the same tissue areas after multiple detection cycles 18
5.3 Combination of all image datasets for visualization 18
5.4 Image export 19
6. Image analysis 20

6.1 Threshold setting for all analyzed RNA transcripts 20
6.2 Definition of regions of interest (ROI) for single cell resolution 22
6.2.1 Nuclear segmentation 22
6.2.2 Creating cell-ROIs by expansion of nuclear-ROIs 23
6.3 Signal quantification 24
6.3.1 Image tiling to reduce file size (optional) 24
6.3.2 SCRINSHOT signal quantification with CellProfiler 25
6.3.3 Stitching of tiled images 26
6.4 Assigning of signal-dots to ROIs 26
7. Data processing and interpretation 27
7.1 Definition of cell-type criteria 28
7.2 Curation of the data (Consideration of false-positive cells) 29
7.3 Creation of cell-type maps 30
8. Literature 32

1. Probe preparation

The genes of interest are selected according to the experimental aims (including optionally a positive control or a house-keeping gene). The SCRINSHOT detection of mRNA is based on a number of steps, for which two types of probes are required: (1) padlock probes to bind specific mRNA sequences and further amplified *in situ* and (2) the fluorophore-labeled detection probe to visualize the amplified specific sequences.

1.1 Padlock probe design

Initially the mRNA sequence of the gene of interest is identified using the:

http://www.ncbi.nlm.nih.gov/gene

The padlock probes contain a constant backbone sequence of 53 nucleotides (nt) and the 5'- and 3'- arms, which are complementary to the corresponding mRNA sequence (Figure 1). The gene-specific arms of padlock probes are around 20nt long each (Tm 50-60ºC), thus the total length of the gene-specific sequence of each padlock is 40nt, which is similar to the length of the Taqman qPCR probes. It is possible to use a Taqman probe design tool such, as the one from Integrated DNA Technologies, Inc.

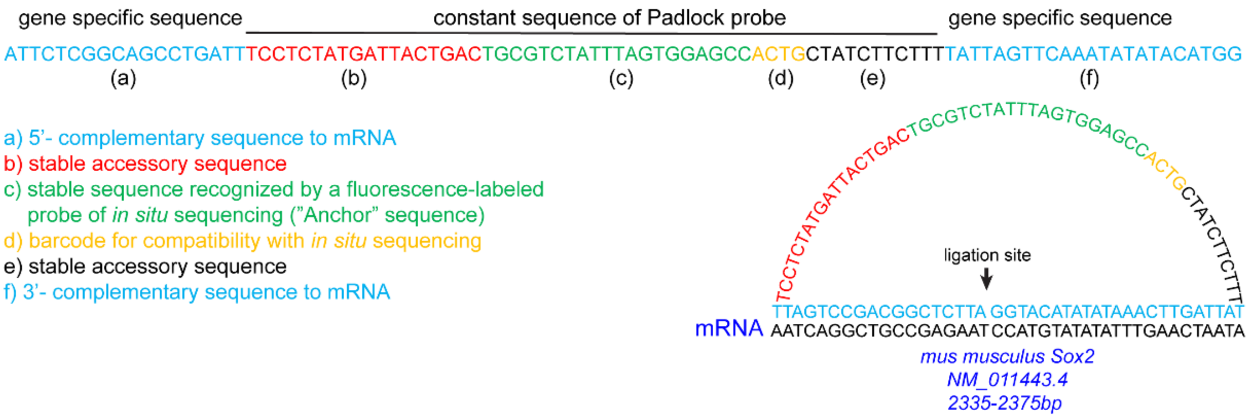

Figure 1. Description of the padlock probe structure. Padlock probes contain two variable gene-specific arms (a, f) and a stable sequence which is subdivided in four parts. Circularized padlock probe is hybridized to the complementary sequence of the corresponding mRNA (mouse Sox2). Padlock probes are designed to be compatible with *in situ* sequencing method [1].

1.1.1 Selecting specific sequences for the padlock probe gene-specific arms

The specificity of the padlock probes is exclusively determined by their arm-sequences. For that reason, it is important to ensure that these probes recognize only the targeted RNA. The following steps provide the necessary information for identifying candidate sequences and how these can be computationally validated.

i. Use the https://eu.idtdna.com/PrimerQuest/Home/Index?Display=AdvancedParams
ii. Choose "Download sequence(s)" using Genbank or Accession ID to import the sequence of the gene of interest. Add the name of the gene.
iii. Choose the qPCR (2 Primers + Probe). For padlock design use the sequence identified ***as probe***:

    a) Results to return: 20
    b) Primer Criteria: Primer Tm (°C) 50-64 (the temperature should be lower than the one of the probe. In general, the parameters should not be too strict to avoid exclusion of suitable Taqman probes). Optimum: 57°C
    c) Probe Criteria: Probe Tm (°C) 65-75 (Optimum: 70°C), Probe CG% 40-60% with optimum 50% and probe size: 40-45nt (Optimum: 45 nt)
    d) Amplicon Criteria: Amplicon Size: 76-500bp (Optimum: 200 bp)

iv. Press "Download Assays" and an *.xls file will be downloaded.
v. Open the file and choose the **probe** sequences (not the primer) for the next steps.
vi. Validate the sequences. This step is important to ensure that the probes are specific for the gene of interest and they **ONLY** bind the desired transcript or transcript variants.
vii. Go to Standard Nucleotide BLAST (Blastn) tool of NCBI (National Center for Biotechnology Information, U.S. National Library of Medicine) and at the "Choose Search Set" field choose the "Mouse genomic + transcript" for **mouse** genes and "Human genomic + transcript" for **human** genes. Also, select the "Somewhat similar sequences (blastn)" in the "Program selection" field: https://blast.ncbi.nlm.nih.gov/Blast.cgi?PROGRAM=blastn&PAGE_TYPE=BlastSearch&LINK_LOC=blasthome
viii. Paste the probe sequence into the field "Enter Query Sequence" and run the Blastn. If the probe is specific, the results with the 100% Query Cover should contain **only** the transcripts of interest. If the results are **plus/minus**, the Taqman probe sequence does not correspond to the mRNA but to its reverse-complement sequence and it should be transformed accordingly, as described in the next step. Ensure that no other RNA sequences, longer than 20 nt, are recognized (in plus/plus direction).

ix. As an additional control, the mRNA sequence, which has been used to create the probes, is aligned with the Taqman probe. In case of plus/minus result, the "Query" sequence is used for padlock probe design (NOT the "sbjct"). Use the function "align two or more sequences" of the Blastn for this step:
x. http://blast.ncbi.nlm.nih.gov/Blast.cgi?PROGRAM=blastn&PAGE_TYPE=BlastSearch&LINK_LOC=blasthome
xi. It is important to use the accession number of the analyzed mRNA as "Query Sequence" and the Taqman probe sequence, as "Subject sequence". Run the blast and then save the obtained "Query" sequence and the corresponding numbers of the first and the last nucleotides in order to know the domain of the transcript, which is recognized by the probe.

Note: 1) Any other freeware or commercial program for probe-primer design can be used (e.g. Beacon design).

2) The following webpage can be used to obtain the reverse and/or complementary sequences:

http://www.bioinformatics.org/sms/rev_comp.html)

1.1.2 Preparation of the Padlock Probes with the "Padlock Design Assistant"

The "Padlock Design Assistant" is a custom script that uses the selected Taqman probe sequences and integrates them, accordingly, to the padlock probe backbone sequence. It can be found at https://github.com/AlexSount/SCRINSHOT.

i. Open the Padlock Design Assistant *.xls file (choose 'Enable macros').
ii. At the "Target Sequence" field paste the validated sequence from the previous steps.
iii. Press the Padlock Design Assistant v1.4 button and adjust the length of the 5'-arm and 3'-arm in order to have Tm=50-60°C (make sure that the difference between the two arms is less than 2°C and Target Tm is approximately 70-80°C).
iv. To obtain the probe press "output" and then "OK".

1.1.3 Padlock probe documentation

Keep track of all the necessary information about the designed probes. Prepare an information report, which should include at least:

i. The name, the accession number and the NCBI-webpage link of the transcript, which has been used to create the probe.
ii. The name of the padlock probe and its complete sequence, as obtained using "Padlock Design Assistant".
iii. The recognized mRNA sequence and its position at the corresponding transcript. The ligation site is highlighted, since it is used for designing of the detection probes (see next section).
iv. The used 4 nt-long barcodes. The sequence can remain constant in all probes but the use of unique sequences for the gene-specific padlock probes makes them compatible with *in situ* sequencing protocol as well [1].

Note: A database software or Microsoft Excel can be used for the documentation.

1.1.4 Ordering of padlock probes

The padlock probes for SCRINSHOT are ordered as 5'-phosphorylated (to facilitate ligation), 4 nmole Ultramer® DNA Oligos, from Integrated DNA Technologies, Inc. Both tube and plate options work fine, but plates are more economical option. Probes are shipped lyophilized and

diluted in 400µl RNA Free H_2O, for 10µM final concentration and stored at -20°C. If you are planning to target more than 39 genes (117 probes, if three probes, per gene, are used), in the same experiment, dilute the padlock probes in higher concentration (20 or 30 µM).

1.2 Detection-oligo design

The detection oligos recognize the gene-specific sequence of the padlock probes. They contain the padlock ligation site in the middle of the sequence. The length of these probes should be adjusted so that their Tm is close to 56°C.

The https://eu.idtdna.com/calc/analyzer tool is used to shorten the mRNA sequence, which has been used for padlock probe design and to calculate its Tm.

i. Go to https://eu.idtdna.com/calc/analyzer tool and paste the mRNA sequence, which has been used for the padlock probe design and press "ANALYZE".
ii. Shorten the sequence by even nucleotide removal from both ends and press "ANALYZE" to see the result. Adjust the produced sequence until its Tm is close to 56°C. It is important to have the ligation position in the middle of the sequence in order to reduce the chances of binding to the unligated padlocks which could create unspecific signals.
iii. Use the reverse-complement sequence from the "COMPLEMENT" field of the OligoAnalyzer result and exchange 2-3 T-nucleotides along the detection probe sequence with U nucleotides. Keep in mind that each "T" to "U" transformation increases the cost of the probe.
iv. Add a fluorophore at the 3'-arm of the sequence and order the oligo (Figure 2). FITC, Cy3 and Cy5 are convenient options due to low cost and standard microscope filter setups. Two more fluorophores (with increased cost) can be simultaneously used together with FITC, Cy3 and Cy5, for example, Texas Red (or analogues) and Atto740 (or analogues). This setup requires suitable microscope filter configuration. The oligos for the current study were ordered from Eurofins Genomics (Figure 2).

The detection probe is the reverse-complement of above analyzed sequence

CAA ATA TAT ACA TGG ATT CTC GGC AGC

↓

Exchange of "T" with "U" to make the probe susceptible to UNG degradation

CAA A<u>U</u>A TAT ACA <u>U</u>GG ATT C<u>U</u>C GGC AGC

↓

Fluorochrome addition to the 3'-arm of the detection probe

CAA AUA TAT ACA UGG ATT CUC GGC AGC-Cy3

Figure 2. Example of detection probe design for the Sox2_m_pr1_SplR probe.

Design a different detection oligo for each padlock probe (each padlock probe for the same gene is binding to different sites on the same mRNA).

For sequential multiplex detection of padlock-probe RCA-products it is necessary to remove the already hybridized and imaged detection oligos. In order to facilitate the destabilization of the probes, exchanging of 2-3 Thymine nucleotides (T) along the detection probe sequence with Uracil nucleotides (U) is essential in oligo design. U nucleotides should be placed less than 10 nt away from each other. They are recognized by the Uracil DNA Glycosylase (UNG) enzyme, which cuts the probe at these positions, producing DNA fragments shorter than 10 nt. The degraded detection oligos are removed with thorough formamide washes.

2. Tissue preparation

Tissues are (1) harvested and fixed, (2) frozen in OCT and (3) sectioned and placed onto pre-coated glass slides. The following example describes the preparation and treatment of mouse lung. The same tissue treatment is used for both immunohistochemical assays and SCRINSHOT. All solutions, except the fixative, should be prepared using RNAse free reagents.

Reagents

PFA4% in PBS pH7.4

Add 60μl NaOH 1M in 45 ml mQ H_2O in a 50 ml FALCON tube.

Warm up the solution in the microwave oven for a few seconds in order to reach 60-70^oC (lid should be loose to avoid explosion).

Add 2 g of PFA powder into the solution and mix well. PFA should completely dissolve. If the particles are still visible, warm the tube in the microwave oven for a few more seconds. **Avoid boiling**.

Add 5 ml 10X PBS (for example, Ambion, AM9625) into the solution and mix by gentle inversion.

Place the solution on ice until it reaches room temperature (approximately 30 min).

Measure the pH using pH-strips and adjust, if needed, to 7.4-7.5.

PBS 1X pH7.4

Add 5 ml PBS 10X (Ambion, AM9625 or analogues) into 45 ml mQ H_2O (or other RNase free H_2O) and mix well.

Sucrose solution

Sucrose powder should be molecular biology grade. Use only disposable spoons to retain RNAse free condition. Prepare 30% sucrose in PBS 1X pH 7.4.

OCT

Cryomatrix Leica FSC22. Disposable syringes should be used for accurate volume measurements.

PFA-OCT mixture (2:1 v/v)

PFA 4% in PBS 1X pH 7.4 – 2 ml

OCT – 1 ml

Sucrose-OCT mixture (2:1 v/v)

30% sucrose in PBS 1X pH 7.4 – 20 ml

OCT (Cryomatrix Leica FSC22) – 10 ml

Isopentane

Sigma-Aldrich 277258-1L

Base Molds

Leica Surgipath Clear Base molds 3803025 or analogues. Different sizes can be used to accommodate tissue size.

2.1 Example of tissue collection and fixation: newborn and adult mouse lung

Euthanize the animal and open the thoracic cavity and perfuse the lung through the heart to remove red blood cells from the organ (left atrium is cut and 1-5 ml of ice-cold PBS 1X pH7.4 is injected through the right ventricle).

Expose the trachea and pass a piece of surgical silk between the trachea and the esophagus in order to create a loose knot. Make a small hole in the trachea using either scissors or a needle and inject a PFA-OCT mixture into the lung using an insulin syringe with 20-24 G plastic catheter (e.g. B Braun 4251130-01) until the tip of the accessory lobe gets inflated. Tighten the knot around the trachea under the position of catheter insertion.

Carefully remove the lung with trachea from thoracic cavity. Do not cut or press the tissue to avoid collapse of the lung and compromised histology. Immerse the tissue in PFA4%, pH7.4 for 4-8 hours (e.g. newborn mouse: 4 hours, young: 6 hours and adult: 8 hours) at 4°C with gentle rotation or shaking.

Extra steps to improve histology (applied to all the tissues)

Transfer the fixed organs into a new tube with a sucrose-OCT mixture and incubate at 4°C for 12-16 hours with gentle rotation or shaking.

2.2 Tissue Freezing

Proper freezing of the tissue is one of the most important steps of the SCRINSHOT because it retains the tissue integrity and prevents tissue section detaching from the tissue slides.

i. Place a small volume of isopentane into a beaker (500 ml) and add small pieces of dry ice into isopentane.
ii. Place the beaker into a container with dry ice at the bottom. The liquid needs 5 min to equilibrate (temperature). Then it is ready to use.
iii. Place the tissue into the plastic mold filled with OCT and place it into the beaker. Leave it until all the OCT becomes white.
iv. Store the tissue blocks at -80°C until sectioning.

2.3 Sectioning

Cut 10μm-thick tissue sections using a cryostat (Leica CM3050S or analogue) and collect them onto poly-lysine coated slides (VWR Cat No. 631-0107), leaving 1.5-2 cm gap between samples (it is optimal to place a single sample per slide in order to provide enough space for sealing the

chamber for *in situ* hybridization). Leave slides to dry in a container with silica gel and then store at -80°C until usage.

3. SCRINSHOT hybridization protocol

The following steps describe the procedure of *in situ* hybridization of padlock probes for all selected genes on the tissue samples, followed by amplification of their sequences and hybridization of fluorophore-labeled detection oligos. The latest step is divided into cycles based on microscope filter setup. After the first set of genes is imaged, the probes are removed and the next set of detection probes is hybridized onto the tissue.

Solutions

Before starting the hybridization, prepare the following solutions:

i. DEPC H_2O. 1 ml DEPC (Sigma-Aldrich, D5758-50ML) is added to 1 L milliQ H_2O and mixed for 12-16 hours using a magnetic stirrer at room temperature (RT). Solution is then autoclaved in order to deactivate DEPC.
ii. 10X PBS (Ambion, AM9625 or analogue).
iii. PBS-Tween 0.05% (500 ml): 450 ml DEPC H_2O + 50 ml PBS 10X + 250 µl Tween 20.
iv. PFA 4%. See "Tissue Preparation" section.
v. 0.1M HCl (30 ml). 30 ml DEPC H_2O and 250 µl 12N HCl.
vi. 70% ETOH (40 ml): 28 ml ETOH and 12 ml DEPC H_2O.
vii. 85% ETOH (40 ml): 34 ml ETOH and 6 ml DEPC H_2O.
viii. 99.5% ETOH
ix. RNAse Free H_2O (Sigma-Aldrich, W4502-1L).
x. 65% Formamide (1 ml): 650 µl deionized formamide (F9037-100ML) and 350 µl RNAse free H_2O. Avoid freeze-thawing formamide more than twice.
xi. 2X SSC (10 ml): 1 ml SSC Buffer 20× Concentrate (Sigma-Aldrich, S6639-1L) and 9 ml RNAse-free H_2O.
xii. Washing buffer (1 ml): 900 µl 2X SSC and 100 µl deionized formamide.
xiii. 6X SSC (20 ml): 6 ml SSC Buffer 20× Concentrate (Sigma-Aldrich, S6639-1L) and 14 ml RNAse-free H_2O.
xiv. Probes: ordered probes are usually lyophilized. Add RNAse-free H_2O and leave to dissolve for at least 4 hours at room temperature or overnight at 4°C. The concentration of padlock probe stock solutions is 10 µM (or higher) and the concentration of detection oligos is 100 µM. All of them are stored at -20°C.

Notes: Ethanol solutions should be freshly prepared, at least for the "Permeabilization and dehydration" step.

Deionized formamide is aliquoted and stored at -80°C. After thawing, it can be kept at -20°C and refrozen one more time.

All frozen reagents are thawed and mixed thoroughly before use. Master mix solutions are prepared in advance and kept at RT, but the enzymes are added last to the mixtures, just before application to the tissue.

Before chamber mounting, all the incubations are done in 50 ml RNAse-free tubes. For all master mix solutions, prepare 10% more (to ensure the sufficient volume for the reaction).

To avoid drying of the tissue, remove the last PBS-Tween 0.05% just before the addition of the next step reaction mix.

3.1 Post-fixation of the slides

i. Remove the slides from -80°C and place them in a small slide box (Sigma-Aldrich Z708313-25EA) for transfer, then immediately place at 45°C for 15 min to prevent moisture accumulation (the lid of the slide box should be open when slides are at 45°C). Place up to two slides per box.
ii. Incubate the slides in 4%PFA (freshly prepared) for 5 min. Use clean forceps to transfer the slides between solutions to avoid contamination.
iii. Wash the slides for 2 x 5 min in PBS-Tween 0.05%.

3.2 Permeabilization of the tissue

i. Incubate the slides in 0.1M HCl at RT for 3 min. Two slides can be placed back to back in a 50 ml FALCON tube. If tissue detaches, reduce the HCl incubation time or its concentration.
ii. Wash the slides for 2 x 2 min with PBS-Tween 0.05%.

3.3 Mounting of hybridization chambers

In order to perform reaction in sterile conditions and facilitate solution application, the hybridization chambers (Gracebio, SA20-0.5-SecureSeal) should be mounted on top of the sample. To ensure the uniform chamber attachment the slide should be dehydrated with series of ethanol. Following the chamber mounting, the sample is rehydrated.

i. Incubate the slides in 70% ETOH for 2 min
ii. Incubate the slides in 85% ETOH for 2 min
iii. Incubate the slides in 99.5% ETOH for 2 min
iv. Place the slides horizontally and leave to dry. Put a protective cover over them to reduce contamination.
v. Peel off the thin adhesive liners of the hybridization chambers and keep them in a box for reuse. Mount the hybridization chambers onto the slides in such way that the holes are along the longer side of the slide. This prevents air trapping when the slides are later immersed in 50 ml FALCON tubes with solution.
vi. wash the slides for 3 x 2 min PBS-Tween 0.05% for rehydration.

Note: To ensure the tissue coverage and to avoid bubbles, hold the slide at a 45° angle (approximately), add and remove the solutions with a pipette through the lower hole of the chamber.

3.4 Blocking

The blocking solution contains tRNA and an Oligo-dT sequence for blocking of probe unspecific binding on the tissue section.

**Blocking master mix**				
**Reagents**	**stock**	**final**	**1 x slide**	**x slides**
RNase-free H_2O			57.5 µl	
Ampligase Buffer	10x	1x	10 µl	
KCl	1 M	0.05 M	5 µl	
Formamide deionized	100%	20%	20 µl	
Oligo-dT	10 µM	0.1 µM	1 µl	
BSA	10 µg/ul	0.2 µg/µl	2 µl	
RiboLock (Thermo)	40 U/µl	1 U/µl	2.5 µl	
tRNA (Ambion AM7119)	10 µg/µl	0.2 µg/µl	2 µl	
**Total**			**100 µl**	

Oligo-dT:AAGCAGTGGTATCAACGCAGAGTACTTTTTTTTTTTTTTTTTTTTTTTTTTTTVN

i. Add the blocking solution into the chamber, mix by gentle pipetting (3-5 times) and incubate the slides at room temperature (RT) for 30 min.
ii. Wash the slides for 2 x 1 min with PBS-Tween 0.05%.

3.5 Hybridization of Padlock probes

Prepare a list of all genes to be detected on the particular tissue sample. The following master mix contains 0.05 µM of each padlock probe for each of the genes. Prepare a pool of all the padlocks targeting the same gene (usually 3) and then use the corresponding volume (1.5 µl of padlock mix for 3 padlocks, 1 µl of padlock mix for 2 etc). If a gene is expressed at high levels (like Scgb1a1 in the lung airways), a lower number and concentration of padlock probes should be used to avoid molecular and optical saturation of the signal (1 padlock and 0.01 µM). Transcript counts from single cell mRNA sequencing experiments provide a useful guideline for this step. It is important to keep in mind that reduction of padlock probe number and concentration might give false negative cells, especially if they express low levels of the detected gene.

Reagents	stock	final	1 x slide	x slides
RNase Free H2O				
Ampligase Buffer	10x	1x	10 µl	
KCl	1M	0.05M	5 µl	
Formamide deionized	100%	20%	20 µl	
Probes 0.5 µl/padlock	10 µM	0.05 µM	0.5 µl x	
BSA	10 µg/µl	0.2 µg/µl	2 µl	
RiboLock (Thermo)	40U/µl	1U/µl	2.5 µl	
tRNA (Ambion AM7119)	10 µg/µl	0.2 µg/µl	2 µl	
**Total**			**100 µl**	

i. Add the solution into the chamber, mix by gentle pipetting (3-5 times) and seal the two holes with PCR adhesive membrane strips (cut to the suitable size) to prevent evaporation.
ii. Incubate the slides at **55ºC for 15 min** for denaturation and at 45ºC for 120 min for hybridization of the probes onto the target mRNA. For this step we use a PCR machine or an Eppendorf Thermostat.
iii. Wash the slides for 3 x 10 min with washing buffer (10% formamide in 2X SSC) to remove unhybridized probes.
iv. Wash the slides for 3 x 1 min with PBS-Tween 0.05% to remove the remaining formamide, as it can deactivate the enzyme in the following step.

3.6 Ligation of Padlock probes

The ligation of the hybridized padlock probes is mediated by SplintR ligase (PBCV-1 DNA Ligase) which can function with DNA:RNA hybrid molecules.

Reagents	stock	final	1 x slide	x slides
RNase Free H_2O			82.5 µl	
T4 RNA Ligase Buffer	10x	1x	10 µl	
ATP	1 mM	10 µM	1 µl	
BSA	10 µg/µl	0.2 µg/µl	2 µl	
SplintR (NEB)	25 U/µl	0.5 U/µl	2 µl	
RiboLock (Thermo)	40 U/µl	1 U/µl	2.5 µl	
**Total**			**100 µl**	

i. Add the solution into the chamber, mix by gentle pipetting (3-5 times) and seal the two holes with PCR adhesive membrane strips to prevent evaporation.
ii. Incubate the slides at **25ºC** for 12-16 hours using a PCR machine or an Eppendorf Thermostat.

iii. Wash the slides for 2 x 1 min with PBS-Tween 0.05%.

3.7 Rolling circle amplification (RCA)

Φ29 polymerase is used to perform the rolling circle amplification (RCA). To avoid interference in the detection of low-abundant genes by the highly abundant ones, we used two distinct padlock probe backbones differing in their anchor sequence, one for low and one for high abundant genes. As a result, two RCA primers, which recognize the corresponding backbones, are used as initiators of RCA. If only one backbone is used, the other one should be omitted from the protocol. The thiophosphate modifications of RCA primers (indicated with "*") prevents the 3'-5' exonuclease activity of Φ29 polymerase, increasing the RCA efficiency [2].

**Reagents**	**stock**	**final**	**1 x slide**	**x slides**
RNase Free H_2O			68.5 µl	
Φ29 buffer (Lucigen)	10x	1x	10 µl	
Glycerol	50%	5%	10 µl	
dNTPs	10 mM	0.25 mM	2.5 µl	
BSA	10 µg/µl	0.2 µg/µl	2 µl	
RCA Primer1 (TAAATAGACGCAGTCAGT*A*A)	10 µM	0.1 µM	1 µl	
RCA Primer2 (CGCAAGATATACG*T*C)	10 µM	0.1 µm	1 µl	
Φ29 polymerase (Lucigen)	10 U/µl	0.5 U/µl	5 µl	
**Total**			**100 µl**	

i. Add the solution into the chamber, mix by gentle pipetting (3-5 times) and seal the two holes with PCR adhesive membrane strips to prevent evaporation.
ii. Incubate the slides at 30°C for 12-16 hours using a PCR machine or an Eppendorf Thermostat.
iii. Wash the slides for 2 x 1 min with PBS-Tween 0.05%.
iv. Add 4% PFA, mix by gentle pipetting (3-5 times) and incubate for 15 min at RT to fix the RCA products on the tissue.
v. Wash the slides for 3 x 1 min with PBS-Tween 0.05%.
vi. Wash the slides for 3 x 10 min with 65% formamide at 30°C on a PCR machine block or an Eppendorf Thermostat.
vii. Wash the slides for 2 x 1 min with PBS-Tween 0.05%

3.8 Hybridization of the first set of detection oligos

The following master mix contains the fluorophore-labeled oligos, which recognize the gene specific domain of the padlock probes. To prevent fluorophore bleaching avoid extensive light exposure after this step.

**Reagents**	**stock**	**final**	**1 x slide**	**x slides**
RNase Free H2O				
SSC	20X	2X	10 µl	
Formamide deionized	100%	20%	20 µl	
FITC-labeled probes	10 µM	0.04 µM	0.4 µl each	
Cy3-labeled probes	10 µM	0.02 µM	0.2 µl each	
Cy5-labeled probes	10 µM	0.02 µM	0.2 µl each	
DAPI	50 µg/ml	0.5 µg/ml	1 µl	
BSA	10 µg/µl	0.2 µg/µl	2 µl	
**Total**			**100 µl**	

i. Add the solution into the chamber, mix by gentle pipetting (3-5 times) and incubate the slides in the dark for 45-60 min at RT.
ii. Wash the slides for 3 x 5 min with washing buffer (see solutions).
iii. Wash the slides for 3 x 1 min with 6X SSC.

Dehydration and mounting

i. Incubate the slides in 70% ETOH for 2 min.
ii. Carefully remove the chambers and incubate the slides in 70% ETOH for 1 min.
iii. Incubate the slides in 85% ETOH for 2 min.
iv. Incubate the slides in 99.5% ETOH for 2 min.
v. Place the slides horizontally and leave to dry in the **dark**.
vi. Apply the SlowFade™ Gold Antifade mounting medium and put the cover-slip on the slides
vii. Store the slides in the dark at 4°C or RT, until imaging.
viii. Image the slides following instructions in part 4.

Note: When the chambers dry, their adhesive liners can be placed back and the chamber can be stored for future reuse.

3.9 Removal of imaged detection oligos, prior to the next hybridization

Immerse the slides in 50 ml FALCON tubes (one slide per tube) with ETOH 70% and place them horizontally in a 45°C oven, with the coverslip facing the bottom, until the coverslip is detached.

i. Incubate the slides in 85% ETOH for 2 min.
ii. Incubate the slides in 99.5% ETOH for 2 min.
iii. Place the slides horizontally on the bench and leave to dry.
iv. Mount the chambers (the chambers can be reused, if their adhesive film is intact).
v. Wash the slides for 3 x 2 min with PBS-Tween 0.05 %.

UNG treatment

The detection probes contain 2-3 U-nucleotides, which can be recognized and removed by Uracil DNA Glycosylase (UNG) enzyme. As a result, the detection probes are destabilized and washed away with the formamide.

**Reagents**	**stock**	**final**	**1 x slide**	**x slides**
RNase Free H2O			86 µl	
UNG buffer	10X	1X	10 µl	
BSA	10 µg/µl	0.2 µg/µl	2 µl	
UNG (Fermentas)	1U/µl	0.02U/µl	2 µl	
**Total**			**100 µl**	

i. Add the solution into the chamber, mix by gentle pipetting (3-5 times) and seal the two holes with PCR adhesive membrane strips to prevent evaporation.
ii. Incubate the slides at **37°C** for 60 min using a PCR machine or an Eppendorf Thermostat. *For removal of the detection probes of highly abundant genes, it is beneficial to do the UNG treatment step twice.*
iii. Wash the slides for 3 x 10 min with 65% formamide at 30°C using a PCR machine or an Eppendorf Thermostat.
iv. Wash the slides for 2 x 1 min with PBS-Tween 0.05%.
v. Add the master mix of the second set of probes and repeat the steps of the hybridization of detection oligos (go to step 3.8).

4. Imaging

In order to detect the probe signal in the tissue, the imaging can be done with a widefield fluorescent microscope at 40x magnification acquiring a full Z-stack of a sample (depending on sample thickness). An exemplar setup is described below.

Zeiss AxioImager Z2 microscope, equipped with a Zeiss AxioCam 506 Mono digital camera and an automated stage. Zeiss LED Colibri2 and external HXP120 light sources are used with the following Chroma filters: DAPI (49000), FITC (49002), Cy3 (49304), Cy5 (49307), Texas Red (49310) and Atto740 (49007). To have an overview of the whole tissue section, a 10x lens, (Zeiss

Plan Apochromat" 10x / 0.45 M27, 420640-9900-000) is used for initial imaging. Higher or lower magnification can be used for this step, depending on tissue size. Then, specific areas of interest are selected and imaged with 40x lens (Zeiss EC "Plan-Neofluar" 40x/1.30 Oil Ph3, 440451-9903-000), using tiling function and Z-stack acquisition. The datasets are saved as *.czi files, using ZEN 2.5 Blue edition software. The coordinates and the acquisition settings (e.g. LED intensity and camera exposure time) of the acquired areas can be recalled from saved *.czi files, allowing the acquisition of the same positions in all detection cycles (there may be a slight shift of sample/stage between cycles, which depends on the stage accuracy and should be less than 500 pixels in both X and Y axes). For the consistency in Z-axis it is useful to use the auto focus function of Zen software and re-adjust for every acquisition.

5. Image preparation for visualization and analysis

Acquisition of the same areas of sample using Zen produces either a set of images in a form of a Z-stack (in case of small sample), or a large number of images (tiles) in a Z-stack (in case of large sample) that should be combined and saved in a suitable format in order to detect the signal co-localization in the same cells. After acquisition the steps of preparation for image analysis are the following:

1. Creation of maximal-orthogonal projections of the *.czi files.
2. Stitching of the tiled images in case of large samples.
3. Alignment of images from all detection cycles using DAPI channels.
4. Combination of all aligned images into one multichannel image to visualize the signal.
5. Export of single-channel aligned images for further signal detection.

5.1 Orthogonal projection and stitching

The orthogonal projection and the stitching, for all detection cycles of the same area are accomplished using Zen blue 2.5 free version, however this setup also works for Zen blue 2.3. It is necessary to activate “Panorama module” in Modules Manager to perform the stitching step. All the stitched projected datasets are saved as *.czi files, which will be used for all the following steps of the analysis (the original files can be stored as a backup).

i. Go to Processing and select the “Orthogonal Projection” method. Set the parameters:

   a) Projection plane: Frontal(xy)
   b) Method: maximum, Start position: 0
   c) Thickness: maximum.

ii. Use the output of "Orthogonal Projection" to do Stitching (only if tiled images have been acquired). Set the parameters in "new output" field:

a) Fuse tiles: Active
b) All by reference (DAPI)
c) Edge detector: no
d) Comparer: Best
e) All other parameters: default

5.2 Image-dataset alignment of the same tissue areas after multiple detection cycles

To be able to measure the number of the detected RNA transcripts with cellular resolution, the images of all detection cycles must be properly aligned. The usage of automated microscope stages, which can recall the coordinates of the acquired areas to reuse them, facilitates this. However, slight shifts, which depend mainly on the stage accuracy, remain in most cases. Nuclear staining (DAPI) serves as a reference. It is used to measure the shift between different acquisitions and to correct it.

i. Export the DAPI channel images of all detection cycles as 8-bit *.tiff files, using "Image Export" method in Zen blue 2.5.
ii. Open files with DAPI images in Fiji [3]. The DAPI image of one detection cycle is used as a reference. The crop should be smaller than the initial image by 1000-2000 pixels in both dimensions. It should not start from position 0 (x=0, y=0), because alignment will be impossible if the stage shift in the other detection cycles has occurred to the left of the reference image. To do the selection, in Fiji: Edit => Selection => Specify.
iii. Crop the DAPI channel image of another detection cycle with the same size as the reference image, but starting from position (x=0, y=0).
iv. To align, prepare a new merged image with the reference DAPI image in one color (for example: red) and the target DAPI image in another color (for example: green). Identify the same nucleus in both images and measure the shift of the same nucleus between the two images using the "Rectangle" tool in Fiji. Save the values of the shift (measure the dimensions of rectangle selection by Edit => Selection => Specify, in Fiji), because they will be used in the next steps of alignment.
v. To validate that the measurement is accurate, prepare a new merged image with the cropped reference DAPI channel, as prepared above and a new cropped target DAPI image, starting from a position with coordinates equal to the shift. Ensure that same nuclei in the two images are superimposed.
vi. Repeat the same procedure for the DAPI channel images of all detection cycles, using the same reference.

5.3 Combination of all image datasets for visualization

Prepare a *.czi file with all detection cycle images as channels. This allows convenient visualization, automated processing and quantification of the signal. For example, if 3 detection

cycles with 3 genes each have been done, a 10-channel *.czi file will be prepared, containing one channel with DAPI and 9 channels with SCRINSHOT signals.

i. Crop and save all the *.czi files according to the identified shift values, using “Create Image Subset” function of Zen blue 2.5. With that function, the DAPI channels are also removed, except for the DAPI channel of the **reference** *.czi file. As a result, the outcome of the merging will contain only one channel with nuclear staining.
ii. In “Info” tab of the *.czi files, modify the “Channel Name” fields, adding the corresponding gene names.
iii. Finally, the modified *.czi files are sequentially merged to one *.czi file using the “Add Channels” function, in Zen blue 2.5. The first channel of the file must be the reference DAPI channel.

5.4 Image export

The measurement of signal-dots is based on the analysis of the images from all channels of the multi-channel *.czi file prepared in the previous step. From this multi-channel *.czi file all the channels are exported as original, 16-bit *.tiff format images, using Zen blue 2.5. For convenience in the next steps of analysis, use the same prefix of the exported file (in the example, it is “hyb1”). Change the folder name to “input” and remove the “zeros” from the names of the first 9 images (e.g. hyb1_c01_ORG.tif => hyb1_c1_ORG.tif).

Prepare an excel file with the information about the analyzed genes, their corresponding channel and gene number (Figure 3). It will be used later for analysis in RStudio.

Preparation of Channel order list

Hyb	Channel Order	Channel	Gene Order	Gene
3	1	DAPI	nuclei	dapi
3	2	FITC	gene1	Cldn18
3	3	Cy3	gene2	Cd74
3	4	Cy5	gene3	Empty-Cy5
1	5	Cy3	gene4	Empty-Cy3
1	6	Cy5	gene5	Ascl1
2	7	FITC	gene6	Empty-FITC
2	8	Cy3	gene7	Etv5
2	9	Cy5	gene8	Lgi3
4	10	Cy5	gene9	Fgfr2
5	11	FITC	gene10	Rfp
5	12	Cy3	gene11	Ager
5	13	Cy5	gene12	Spry2
6	14	FITC	gene13	Scgb1a1
6	15	Cy3	gene14	Sftpc
6	16	Cy5	gene15	Lyz2
7	17	FITC	gene16	Cyp2f2
7	18	Cy3	gene17	Axin2
8	19	FITC	gene18	Calca
8	20	Cy3	gene19	Napsa

Figure 3. Example of a list with all analyzed genes.

6. Image analysis

The single channel images are further analyzed for the presence of signal-dots in specific regions of the sample. In order to do that, the regions of interest (ROI) and the signal thresholds need to be defined, followed by quantification of signal per defined ROI. Following steps are performed:

1. Definition of signal-dots (threshold setting based on shape and intensity of the signal)
2. Definition of ROI (nuclear segmentation and expansion)
3. Signal quantification
4. Assigning signal-dots to ROI

The analysis of the images requires Zen blue 2.5, MATLAB 2017b (with Image Processing Toolbox), CellProfiler 3.1.5 (version 3.1.9 is also suitable), Fiji and R with RStudio.

6.1 Threshold setting for all analyzed RNA transcripts

The analysis of SCRINSHOT signal-dots is based on the “fixed_probe_analysis_pipeline_V5_1-19genes_CP315.cpproj” custom CellProfiler script, which identifies and measures fluorescence signals with specific intensities and sizes. To determine a suitable threshold for each gene, a CellProfiler [4] custom pipeline was created (“threshold_setting_v1.cpproj”) (https://github.com/AlexSount/SCRINSHOT). It tests how many signal-dots are recognized using different thresholds. The analysis is first done in a small representative area, which includes positive cells for all the analyzed markers. If not possible, multiple areas can be used to provide a more accurate result. The most convenient way to prepare the images for the threshold analysis is to do a crop of the *.czi file, using the “Create Image Subset” function of Zen blue 2.5 and export the images as described above (section 5.3).

To run the “threshold_setting_v1.cpproj” script, drag and drop the image of each gene for analysis at the “Images” section of the pipeline in CellProfiler. Then, set an output directory where the results are saved (“View Output Settings” tab). Name the output folders according to the corresponding analyzed gene.

The script produces a number of *.csv files in the “output” directory, containing the coordinates of the identified signal-dots, but the summary is included in the “MyExpt_Image.csv” file. The “Count_IdentifiedBlobs_thrs” values correspond to the number of identified dots for the tested thresholds. The “threshold_test” folder in the “output” directory contains merged images of the raw-signals (red) and the identified signal-dots (green) for every threshold. In the example in Figure 4, different thresholds (0.002-0.038) were tested to identify a suitable one for every gene.

As expected, low threshold values detect background fluorescence levels and high threshold values are correlated with loss of positive signal.

In conclusion, this step provides a method to estimate proper threshold values for signal-dot identification and measurement for the analyzed genes. Images and quantitative results in *.csv files provide complementary information and it is necessary to **interpret both** for proper threshold choice.

Optionally, the same area can be acquired without any detection oligo for the three used fluorophores (FITC, Cy3 and Cy5), allowing more accurate distinction of signal from tissue autofluorescence (Figure 4 B).

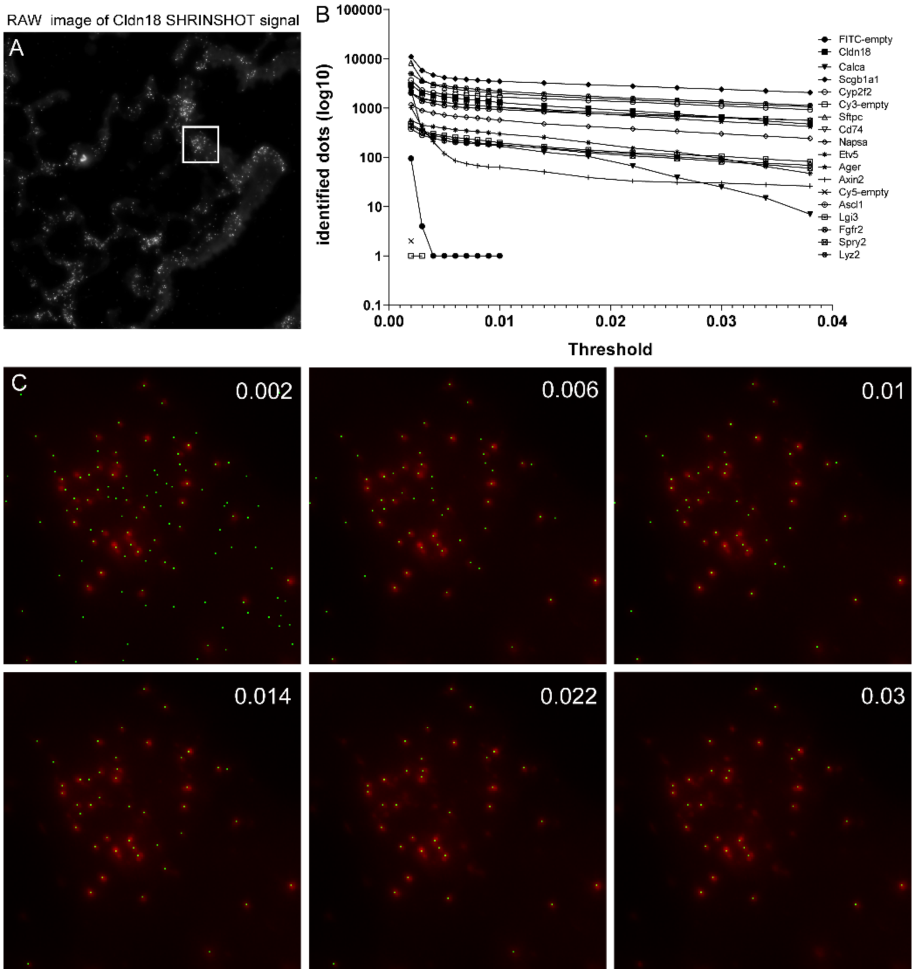

Figure 4. Example of the analysis for threshold estimation, with “threshold_setting_v1.cpproj” CellProfiler script. (A) Cldn18 RAW image of SCRINSHOT signal, which is used as example for the threshold analysis. (B) Histogram of the signal-dot identification, as threshold values increase. The y-axis is log10 transformed for better visualization. (C) Six representative images of the analysis, showing the fluorescence signal (red) and the identified signal-dots (green). The corresponding thresholds are written in the images.

6.2 Definition of regions of interest (ROI) for single cell resolution

To add single-cell resolution in SCRINSHOT analysis, it is necessary to set specific regions of interest (ROIs), which are accounted as cells and register the identified signal-dots of the detected RNA transcripts to them. Cell segmentation on tissue sections is one of the most challenging tasks for spatial gene-expression methods, because of the difficulty to identify the real cell borders (for example the alveolar region of adult mouse lung contains overlapping and elongated cells, making the real cell border definition almost impossible even with 3D confocal imaging strategies). To circumvent this problem, ROIs are based on nuclear outlines, which are (i) segmented manually and (ii) expanded by 2 μm in all dimensions (avoiding the overlap of cell regions). ROIs represent a region around the nucleus (including the nucleus itself) that contains most SCRINSHOT signals, further referred to as cell-ROIs. To create quantitative data, we registered the detected signal-dots of all genes to these cell-ROIs, producing count matrices.

6.2.1 Nuclear segmentation

Segmentation of all nuclei in the analyzed dataset is done with ROI Manager in Fiji, using the reference DAPI channel (hyb1_c1_ORG.tif). The procedure is time consuming but digital pen devices can help. The result is saved as a *.zip file, which contains *.roi files, corresponding to all drawn nuclear ROIs. The *.roi files can be extracted and used partially, but in order to be used by ROI Manager in Fiji, they have to be in a *.zip file format. It is important to avoid overlapping nuclear ROIs because their intersection will be recognized as a separate object.

After completion of nuclear segmentation, Fiji is used to create a binary image with nuclear outlines (Figure 5), which will be used in the following steps. To create the binary image:

i. Create a white RGB image with the same dimensions as the analyzed image.
ii. Change the color options in Fiji (edit=>options=>colors: Foreground:black, Background:white, Selection:white).
iii. Import in Fiji the saved .zip with the *.roi files.
iv. In ROI manager=> properties: stoke color:white, width:0, fill color: black, show outlines.
v. Flatten the image and in ROI manager=> properties: stoke color:white, width:2, fill color:none. This step introduces some distance between the neighboring ROIs.
vi. Flatten the image.
vii. Process=> Binary => Make Binary (in options of this tab, ensure that “Black background: is activated and the colors are not inverted)
viii. Save the image as “hyb1_c1_ORG.tif” and use it to replace the DAPI channel in the “input” directory. The original DAPI channel is saved for future reference.

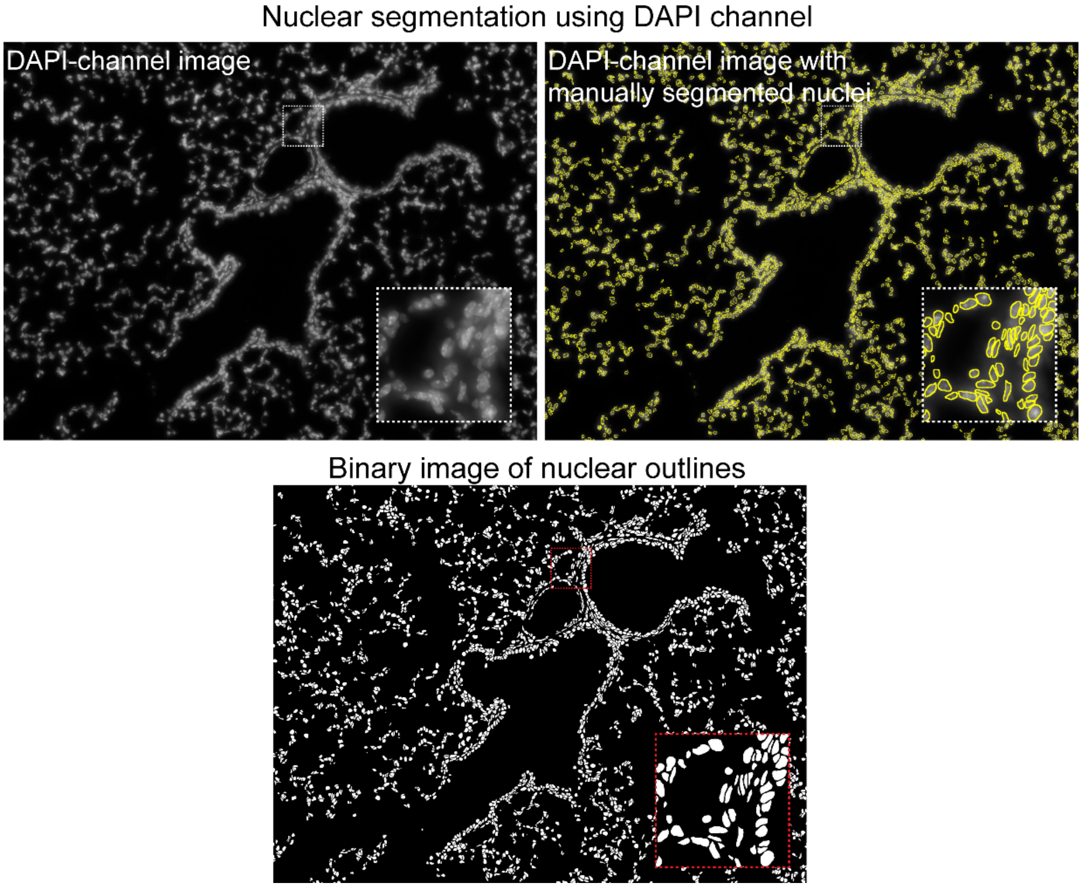

Figure 5. Manual segmentation of all nuclei in acquired tissue areas and creation of a binary image with the nuclear outlines.

6.2.2 Creating cell-ROIs by expansion of nuclear-ROIs

In order to partially capture cytoplasmic region around the nucleus, which contains SCRINSHOT signals, the "expand_nuclei_V2.cpproj" CellProfiler script (https://github.com/AlexSount/SCRINSHOT) uses the binary image with nuclear outlines from the previous step to expand the ROIs by 2 μm without tiling (Figure 6).

i. Open the "expand_nuclei_V2.cpproj" script in CellProfiler.
ii. Drag and drop the binary nuclear-ROI image (not tiled) into the "Images" field.
iii. Set the output folder at the "View output settings" tab.

The produced "Cell_outlines1.tiff" image is used in Fiji to create a new cell-ROI mask with the "Analyze Particles" function. The results are introduced to ROI Manager and saved as *.zip, similarly to the nuclear ROIs in previous step (Figure 6).

i. Analyze => Analyze Particles: Size (pixel^2):100-infinity, Circularity:0-1, Show: nothing, Display Results: yes, Add to Manager: yes
ii. ROI Manager: more=> save as "507_s7_all_cell_rois.zip" (for our example)
iii. ROI Manager: more=> list => save "Ovelay Elements" as "all_cell_roi_list.csv"

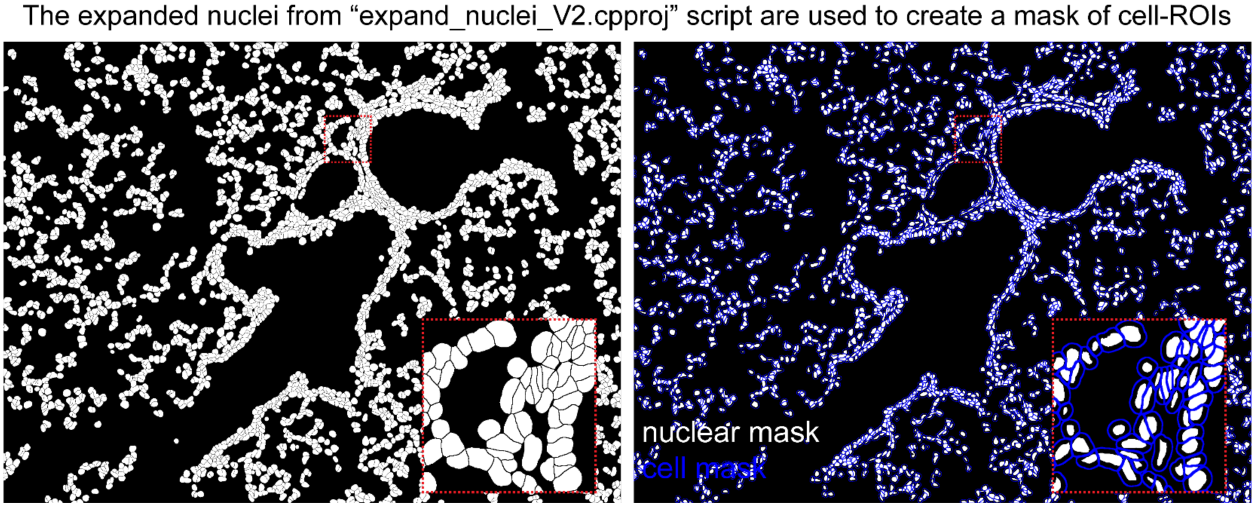

Figure 6. Characteristic example of expanded nuclear-ROIs with "expand_nuclei_V2.cpproj" and ROI-Manager in Fiji. In the right panel, nuclear mask: white, cell mask: blue.

6.3 Signal quantification

The SCRINSHOT signal quantification requires the recognition of the fluorescent dots and their registration to the identified cell-ROIs.

6.3.1 Image tiling to reduce file size (optional)

Large images might require sufficient RAM in the computer to be processed by CellProfiler in further steps. In that case, it is necessary to reduce the file/image size, dividing it into smaller regions, which facilitates processing. **Use unexpanded nuclei ROIs for these steps.** The "tiling_nuclei_genes.m" MATLAB script (https://github.com/AlexSount/SCRINSHOT) crops and saves smaller sequential images for each channel. It also produces a *.csv file with the tile coordinates in original image, which is used by CellProfiler in the next step of the analysis.

*Note: * MATLAB requires a provided function (Tiling_Sequencing_2.m) in insitu.zip file. This file should be extracted into the MATLAB folder, which is created by default in "Documents" during MATLAB installation. The script is part of the in situ sequencing pipeline analysis [1, 5].*

The "tiling_nuclei_genes.m" opens in MATLAB and the working directory is set to the folder with the Tiling_Sequencing_2.m function, using the "Browse for Folder" option. In the given example the path is "C:\Users\alex\Documents\MATLAB\insitu".

i. The directory with the input images is set. It is preferential to use the full pathway. In the given example, it is: "C:\Users\alex\Desktop\SCRINSHOT_paper\analysis_procedure\507_s7\input".
ii. The total number of input images should be set in the "channel_max" field. In the example, it is 16 (15 genes and the ROI channel). Also the tile size should be determined at that step. In the example, it is 2500x2500 pixels. This size does not require a powerful computer for the next steps of the analysis with CellProfiler.
iii. The name of the *.csv file with tile coordinates is set in addition to the channel names, which should be "Nuclei" for the first DAPI channel and then gene1, gene2, …geneN (N=the number of the detected genes).

6.3.2 SCRINSHOT signal quantification with CellProfiler

The custom CellProfiler script, "fixed_probe_analysis_pipeline_V5_1-19genes_CP315.cpproj" is used to automatically count the dots of all genes in the analyzed dataset. The provided *.cpproj file has been written and tested with CellProfiler 3.1.5 and performs the analysis of 19 genes as a default (https://github.com/AlexSount/SCRINSHOT). **Use unexpanded nuclei ROIs for these steps.** For more genes, more modules can be added by copy/paste in the script and for fewer genes, modules can be deleted or deactivated. To run the analysis:

i. Set the input (folder with the tiled images) and the output (folder for saving the results) directories from "View Output Settings" tab.
ii. In "Name of the file" field of "LoadData" module use the *.csv file, which has been produced by MATLAB (for example, 507_s7.csv).
iii. Set the "Manual threshold" value in the "IdentifyPrimaryObjects" module for each gene, as it has been determined in the corresponding section. The threshold for each given gene should be the same for all analyzed datasets of the same experiment.

Note: Download and install Java SE Development Kit 8 (tested version 8u231). Then install CellProfiler and in File => Preferences, increase the "Maximum number of workers" and the "Maximum memory for Java(MB)" to approximately 80% of the number of Logical Processors and RAM amount of your system, respectively. This will significantly reduce the waiting time for analysis completion.

After completion of the analysis, in the output directory, the "Cell_outlines" folder contains the tiles of the binary image with 2 µm-expanded nuclei, which are counted as cell-ROIs. There are also

two types of images with the identified signals, the "1pixel" and the "vis". The '1pixel' depicts the identified signals as 1pixel dots and will be used for the next analysis steps. The 'vis' depicts identified signals as bigger dots for visualization purposes (the size and the shape can be adjusted in the "DilateImage" modules of the script). The "output" directory contains also *.csv files with the coordinates of all identified signals and their correlation with the cell-ROIs.

6.3.3 Stitching of tiled images

To identify which and how many signal-dots are localized inside each cell-ROI, the "automated_stitching_dot_counting_v1_19genes.ijm" custom Fiji script has been prepared (https://github.com/AlexSount/SCRINSHOT). It automatically stitches the tiles [6] of the 1pixel image results, using the "Grid/Collection Stitching" Plugin and measures the Mean Fluorescence Intensity (MFI) of the 1pixel signal dots in the cell-ROIs of the previous step.

i. Open the "automated_stitching_dot_counting_v1_19genes.ijm" script in Fiji.
ii. Create an output directory and set its full path in line-1 (in the example, it is "C:/Users/alex/Desktop/SCRINSHOT_paper/analysis_procedure/507_s7/s7_1pixel_images/".
iii. Set the path to *.zip file with the cell-ROIs from the previous step in line 3.
iv. Set the path of the folder with the 1pixel images of gene 1 in line 5.
v. In line 9, set the grid_size_x and grid_size_y values. In the example, the original image is 6700x5000 and the tile size is 2500x2500, so the grid_size_x=3 and the grid_size_y=2. Set also the path of the "directory=" to the path with the 1pixel images.
vi. In line 10, set the width and height values according to the dimension of the original image (in the example, it is 6700x5000).

6.4 Assigning of signal-dots to ROIs

The following step contains the transformation of MFI values to signal-dots and the creation of count matrices for all genes in all pre-defined cell-ROIs. The previous step produces one image and one *.csv file for every gene. The image has the same dimensions as the original dataset but shows the identified signal as 1pixel dots. The *.csv file contains seven columns: "no-title": ascending measurement number, "Area": the cell-ROI surface area in pixels^2, "Mean": mean fluorescence intensity in each cell-ROI, "Min": minimum fluorescence intensity in each cell-ROI (8-bit), "Max": maximum fluorescence intensity in each cell-ROI (8-bit), "X" and "Y": the coordinates of the cell-ROI center. From these values, the "Area" and the "Mean" will be used for the following steps.

The most convenient way to proceed is using R (tested with 64-bit v3.5.2) and RStudio (tested with v1.1.463). The script "SCRINSHOT_script_v2.R"

(https://github.com/AlexSount/SCRINSHOT) uses the results of the previous step to prepare a count matrix with the SCRINSHOT dots for all the genes in all cells. It provides a detailed description of all steps, which allows easy modifications, if necessary.

i. Set the working directory. Rstudio will load and save files at this folder for the whole analysis. If a file is located at another directory, the full path of the file is required (e.g. line 31).
ii. Install the indicated packages, following the steps in lines 10-15. This step is done only once.
iii. Load the packages, which are required for the analysis (lines 18-21).
iv. Export a list with all the cell-ROIs through ROI manager as a *.csv file and name it: "all_cell_roi_list.csv". To do that load the *.zip file of cell-ROIs (NOT the nuclear-ROIs) into Fiji and in ROI-Manager => More => List => save as *.csv.
v. Load the created cell-ROI list (line 25). Keep the column with the ROI-names and name it as "ROI" (lines 27-28).
vi. Import and create another column with cell-ROI surfaces in pixels^2. Name it "Area" and merge it with "ROI" (lines 31-34).
vii. Merge the *.csv files with the MFI values of the identified dots for all genes (lines 45-51).
viii. Import the list with the channel-gene correlation (line 37), which has been created before and use it to modify the gene names (lines 55-73).
ix. Transform the MFI-values to dots for each gene (lines 76-80). The transformation is based on the fact that the 1pixel images have 8-bit color-depth and that each dot has intensity 255. The mean fluorescence intensity of the cell-ROIs stems from the division of the number of the 1pixel dots (with intensity 255) by the surface of the corresponding cell-ROI in pixels^2. As a result, the reverse transformation gives the number of the measured dots.
x. A matrix of the MFI for each gene, in each cell-ROI is also produced (lines 94-96).
xi. The script contains an example of count-matrix preparation for the AT2 subset of the cell-ROIs. It is recommended to import a list of the specific cell-ROIs, which has been prepared using ROI-Manager in Fiji, as described above (lines 100-111).

Note: Microsoft Excel can be used instead of R, but if the analysis includes large number of genes and cell-ROIs, R is more efficient in both error controls and RAM usage.

7. Data processing and interpretation

Obtained results can further be processed and interpreted according to the experimental aims. The following example describes the separation of the defined cell-ROIs into cell types and further determination of cell states based on the positivity for certain selected genes, as well as creation of cell-type maps.

7.1 Definition of cell-type criteria

SCRINSHOT allows the detection of multiple genes on the same tissue section. The creation of the nuclear ROIs and their 2 μm-expanded version, cell-ROIs, allows the measurement of the detected genes in them in a quantitative manner.

Spatial annotation of distinct cell-types can be done with the detection of selective markers for them. The procedure is based on the application of specific criteria to the analyzed cell-ROIs in Microsoft Excel or R. For example, the *Sftpc*^{pos} *Lyz2*^{pos} *Scgb1a1*^{neg} cell-ROIs can be annotated as AT2 cells [7].

To consider a cell-ROI as positive or negative for a distinct gene, a threshold strategy based on the abundance of the dots of this gene, is suggested. Considering that SCRINSHOT dots do not follow a canonical distribution, similarly to the zero-inflated data of single-cell RNA sequencing, the 10% of the maximum number of detected dots per cell-ROI for a specific gene, is used as threshold for that gene (upper limit was set to 3 dots per cell). In particular:

i. Max: 0-10 threshold=0 (the cell should have 1 and more dots of a gene to consider it as positive).
ii. Max: 11-20 threshold=1 (the cell should have 2 and more dots of a gene to consider it as positive).
iii. Max: 21-30 threshold=2 (the cell should have 3 and more dots of a gene to consider it as positive).
iv. Max: 31 and more threshold=3 (the cell should have 4 and more dots of a gene to consider it as positive).

If all the selected genes (*Sftpc*, *Lyz2* and *Scgb1a1*) are highly expressed, a cell is considered to be positive if it contains four or more dots. Application of (*Sftpc*^{pos} & *Lyz2*^{pos} & Scgb1a1^{neg}) criterion in Microsoft Excel is done using the COUNTIF function. To do that, write in the "all_cell_dots.xlsx":

For *Sftpc* in cell V2: =countif(H2; ">3")

For *Lyz2* in cell W2: =countif(I2; ">3")

For *Scgb1a1* in cell X2: = countif(G2; "<4")

For sum of all 3 in Y2: =sum(V2:X2)

For example, the cell-ROI "0001-0009.roi" will be considered **AT2** only if the **Y2 value is 3**.

The application of the above criteria to all cell-ROIs returned 783 positive hits. Selection of the positive *.roi files from the "507_s7_all_cell_rois.zip" allows the visualization of these cell-ROIs

on an image with the expression of one or many genes (DAPI can be omitted but it is helpful). To do that:

i. Extract the “507_s7_all_cell_rois.zip” file and create a folder, containing the *.roi files, with a path
ii. “C:\Users\alex\Desktop\SCRINSHOT_paper\analysis_procedure\507_s7\507_s7_all_cell_rois”.
iii. Create a new folder with a path “C:\Users\alex\Desktop\SCRINSHOT_paper\analysis_procedure\507_s7\at2_rois”.
iv. Open a new, empty Microsoft excel file and paste the names of all the cell-ROIs (783, in this case) at the second column, starting from B2. In A1 write “cd” *plus the full path of the folder from step (i)*. In the other cells of column A write “copy”. In column C, starting form C2, write “.roi” *plus the full path of the folder from step* (ii).
v. Copy-all in a text editor, like gedit and transform the strings to be able to function as commands in terminal. In Microsoft Excel the columns are tab-delimited, tabs have to be removed in gedit. This can be easily done with “Find and Replace” function. The “copy\n” is changed to “copy “ and the “\n.roi” with “.roi”.
vi. Select and copy-all to clipboard and paste by right-click in Microsoft Windows Command Prompt. Preferentially, use the internal disk with the Microsoft Windows installation.
vii. When the *.roi files have been transferred to the “at2_rois” folder, select and copy-all of them and paste in a new Compressed (zipped) folder, in this case named “507_s7_at2_rois.zip”. Avoid subfolders because the Fiji ROI Manager will not recognize the *.roi files.

7.2 Curation of the data (Consideration of false-positive cells)

The cell-ROI definition as an expansion of the nucleus partially recapitulates the real cell shape. The problem is more profound in tissue sections for a number of reasons, such as overlapping cells with different size and morphology. As shown in Figure 7, there are some false-positive cells due to the dots from adjacent true-positive cells being erroneously assigned. If the signal misrepresentation is obvious, the problematic cell-ROIs are manually removed (not corrected), since these false-positive cells may be accounted as technical noise. The alternative application of stricter criteria may cause the loss of cell-ROIs with low expression levels for the interrogated genes. To test the data for false-positive cells:

i. Create a merged image, showing the raw SCRINSHOT signals of the criteria genes.
ii. Open the merged image and the *.zip file (in the example, it is the “507_s7_at2_rois.zip”) with cell-ROIs in Fiji ROI Manager and delete the problematic cell-ROIs. Note that curation is best done with highly abundant genes, because their signal distribution indicates the cell shape.
iii. ROI Manager: more=> save as “507_s7_at2_curated_rois.zip”.
iv. ROI Manager: more=> list => save “Overlay Elements” as “507_s7_at2_curated_roi_list.csv”

Follow the instructions of "SCRINSHOT_script" in Rstudio to create new matrices with the dots of the edited AT2 cells, as described in the section 6.4.

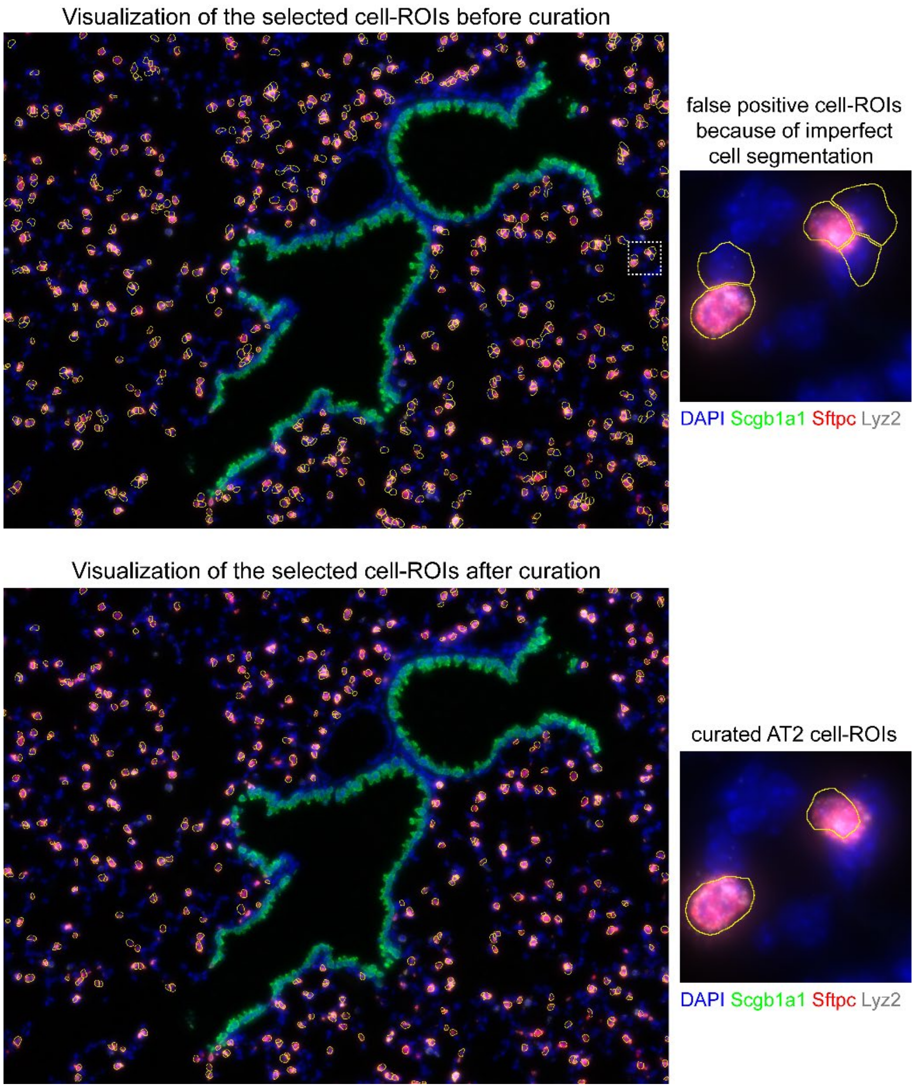

Figure 7. Characteristic example of preferential selection of *Sftpc*^{high} cells for further separation according to *Lyz2* and *Scgb1a1* expression. AT2 cell-ROIs are shown before and after manual curation. The problematic cell-ROIs were removed from the analysis.

7.3 Creation of cell-type maps

The ability of SCRINSHOT to detect the expression of many genes allows the creation of cell-type spatial maps, based on cell type markers. As described above, distinct criteria can be used to identify cell types, for example, AT2 cells. The creation of *.zip files with the *.roi files of interest facilitates visualization of them with specific colors using Fiji ROI-Manager (Figure 8). To do that:

i. Create a black RGB image with the dimensions of the analyzed dataset
ii. Open the *.zip file with the original cell-ROIs
iii. In ROI Manager => properties: stroke color=blue; fill color=blue =>flatten
iv. Open the *.zip file for AT2 cells using this flattened image and follow the same procedure using another color
v. Do the same for all cell categories and save the image.

Fiji provides up to 12 distinct colors for staining of the different cell types, using ROI Manager. For more colors, Zen Blue 2.5 allows unlimited number. To add colors in Zen, prepare separate images for each cell type (8-bit, binary) and import them into Zen. Merge them using the “Add Channels” module and color them accordingly.

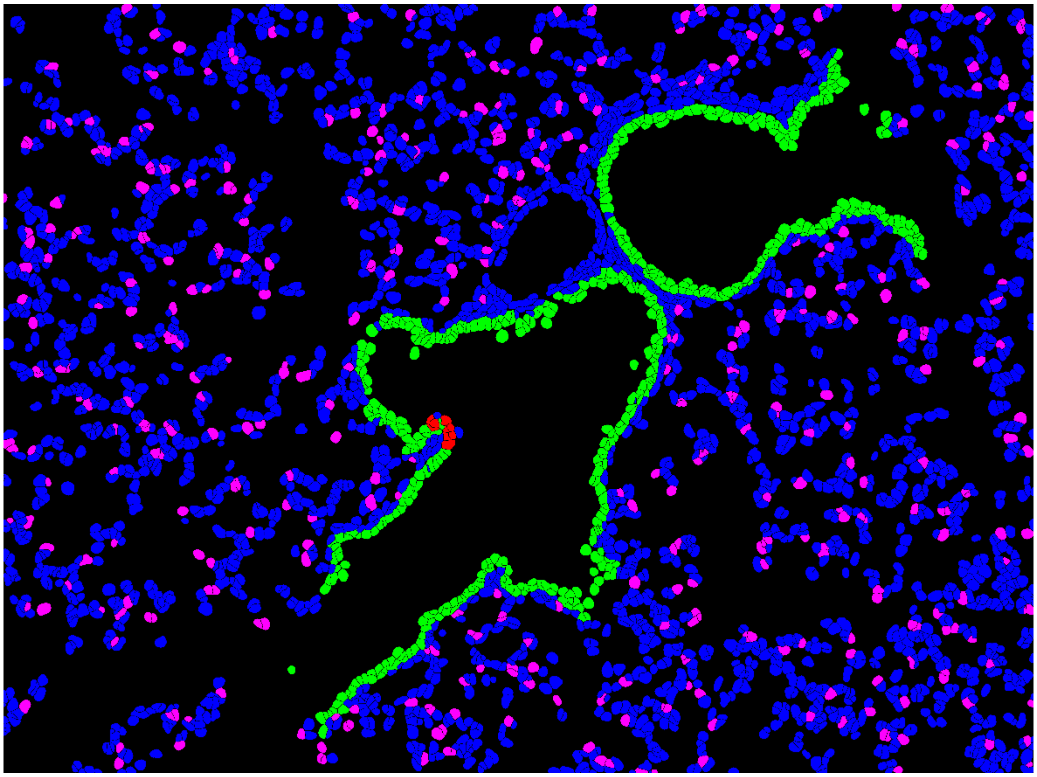

Figure 8. Example of cell type map. Blue: not-annotated cell-ROIs, green: *Scgb1a1*^{pos} club cells, red: *Ascl1*^{pos} neuroendocrine cells and magenta: *Sftpc*^{pos} & *Lyz2*^{pos} & *Scgb1a1*^{neg} AT2 cells.

8. Literature

1. Ke R, Mignardi M, Pacureanu A, Svedlund J, Botling J, Wahlby C, et al. In situ sequencing for RNA analysis in preserved tissue and cells. Nat Methods. 2013;10(9):857-60. Epub 2013/07/16. doi: 10.1038/nmeth.2563. PubMed PMID: 23852452.
2. Dean FB, Nelson JR, Giesler TL, Lasken RS. Rapid amplification of plasmid and phage DNA using Phi 29 DNA polymerase and multiply-primed rolling circle amplification. Genome Res. 2001;11(6):1095-9. Epub 2001/05/31. doi: 10.1101/gr.180501. PubMed PMID: 11381035; PubMed Central PMCID: PMCPMC311129.
3. Schneider CA, Rasband WS, Eliceiri KW. NIH Image to ImageJ: 25 years of image analysis. Nat Methods. 2012;9(7):671-5. Epub 2012/08/30. PubMed PMID: 22930834; PubMed Central PMCID: PMCPMC5554542.
4. McQuin C, Goodman A, Chernyshev V, Kamentsky L, Cimini BA, Karhohs KW, et al. CellProfiler 3.0: Next-generation image processing for biology. PLoS Biol. 2018;16(7):e2005970. Epub 2018/07/04. doi: 10.1371/journal.pbio.2005970. PubMed PMID: 29969450; PubMed Central PMCID: PMCPMC6029841.
5. Svedlund J, Strell C, Qian X, Zilkens KJC, Tobin NP, Bergh J, et al. Generation of in situ sequencing based OncoMaps to spatially resolve gene expression profiles of diagnostic and prognostic markers in breast cancer. EBioMedicine. 2019. Epub 2019/09/19. doi: 10.1016/j.ebiom.2019.09.009. PubMed PMID: 31526717.
6. Preibisch S, Saalfeld S, Tomancak P. Globally optimal stitching of tiled 3D microscopic image acquisitions. Bioinformatics. 2009;25(11):1463-5. Epub 2009/04/07. doi: 10.1093/bioinformatics/btp184. PubMed PMID: 19346324; PubMed Central PMCID: PMCPMC2682522.
7. Treutlein B, Brownfield DG, Wu AR, Neff NF, Mantalas GL, Espinoza FH, et al. Reconstructing lineage hierarchies of the distal lung epithelium using single-cell RNA-seq. Nature. 2014;509(7500):371-5. Epub 2014/04/18. doi: 10.1038/nature13173. PubMed PMID: 24739965; PubMed Central PMCID: PMCPMC4145853.
