# Supplementary material for: SCRINSHOT enables spatial mapping of cell states in tissue sections with single-cell resolution
Source: PLoS Biol. 2020 Nov 20;18(11):e3000675. doi: 10.1371/journal.pbio.3000675 (PMC7717588; doi:10.1371/journal.pbio.3000675)
Supplement: Attachment — Submitted filename: Response to Reviewers.docx [file pbio.3000675.s017.docx]

Dear Dr. Roberts and Reviewers,

Thank you very much for providing us the opportunity to submit a revised version of our manuscript for publication to PLoS Biology. We have now revised the manuscript following your constructive criticism and helpful suggestions.

Below, we include a point by point reply to each of the reviewers’ comments, where we explain the new experiments and analyses, we added to the revised version.

In summary (following the text order):

1) In response to Reviewer#2, we introduced in the legend of Figure 2 the p-values of a multiple comparison test regarding the *Actb* detected dots per cell of the analyzed conditions.
2) In response to Reviewer#3, we more thoroughly analyzed the SCRINSHOT performance, regarding the mismatches at the padlock probe-ligation sites by introducing mutations at the ligation sites of Cyp2f2 and Etv5 padlock probes.
3) In response to Reviewer#2, we provided additional information about signal saturation of extremely expressed genes and how it can be avoided or reduced.
4) In response to Reviewer#1, we examined SCRINSHOT sensitivity for detection of endothelial cell markers, including *Cldn5 and Cdh5,* except for the previously analyzed *Pecam1.* The smooth muscle marker *Acta2* has been also used to stain the vascular smooth muscle cells, in addition on negative control genes, that are not expressed in the analyzed tissue.
5) In response to Reviewers #1 and #3, we did Sftpc immunostaining and RFP fluorescence analysis of lung sections from *Sftpc-CreER*^pos^;*Rosa-Ai14*^pos^ mice to quantitatively estimate the recombination efficiency in alveolar type 2 cells (AT2) in the specific tissue and used the RFP protein fluorescence as “ground truth” to evaluate SCRINSHOT specificity and sensitivity.
6) In response to Reviewer#1, we analyzed the same NEB of initial Suppl. Figure 4 with high resolution confocal microscopy to examine the signal overlap between club-cell *Scgb1a1* and neuroendocrine-cell (NE) *Calca* and *Ascl1*. We independently analyzed NEBs with immunofluorescence for the club cell marker Scgb1a1 (CC10), the neuroendocrine cell marker Calca (CGRP) and the epithelial cell border marker E-cadherin to examine the marker signal overlap at protein level, also.
7) In response to Reviewer#3, we did pairwise correlations of the AT2 selective markers in all analyzed cells (without any cell selection) and compared them with the club cell markers *Scgb1a1* and *Cyp2f2* to show positive and negative correlations.
8) In response to Reviewer#3, we used SCRINSHOT signal of the moderately expressed *Etv5* for definition of AT2 cells and compared the assay performance with published scRNA-Seq data.
9) In response to Reviewer#3, we estimated the percentage of tracheal cells, being annotated to the analyzed cell-types that express their SCRINSHOT-targeted markers and the markers of other cell-types. We also re-analyzed the published tracheal scRNA-Seq dataset of Montoro et. al., using their cell annotation to obtain the same information as for SCRINSHOT and directly compared the results.
10) In response to Reviewers #1 and #3, we used hierarchical clustering as an unsupervised method for cell annotation in addition to the originally utilized “annotation criteria” supervised method to show that this alternative can also successfully be used.
11) In response to Reviewer#2, we provided a detailed estimation of SCRINSHOT cost and compared it with commercially available solutions.

We are looking forward to your responses and the decision on the acceptance of our manuscript for publication.

Sincerely,

Alexandros Sountoulidis and Christos Samakovlis

Reviewer #1:

In their manuscript, Sountoulidis and colleagues report a technology called Scrinshot, a spatial method for mapping single cell states in tissue. The main merit of this in situ hybridization technology is low cost and multiplexing capacity. However, the sensitivity is not well characterized, the specificity appears poor, and the overall performance of generating single cell profiles is very low. The dataset of mapped cells does not appear close to the purported sensitivity of cell identification based on representative images provided of different cell types, and the profiling approach cannot even be applied to tissues or regions where cells are not of uniformly cuboidal morphology, like lung alveoli. Finally, there is considerable manual manipulation with processing of the primary fluorescent data with a puncta thresholding approach that is not validated, and adjudication of autofluorescence is required, introducing the possibility of bias.

Major criticisms

1) The sensitivity is benchmarked against surfactant C which is perhaps the most highly expressed gene in the lung. Despite the massive expression levels of this transcript, no signal was detected in 1.1% of the genetically marked alveolar type II cells.

*Sftpc* is abundantly expressed in the epithelial AT2 cells of the lung alveoli. It is considered the typical AT2 marker [1], and we therefore used the *Sftpc-CreER*-*Rosa-Ai14* knock-in reporter to evaluate SCRINSHOT specificity and NOT sensitivity in the method as the reviewer writes. To address the reviewer´s concern that 1.1% of the RFP labelled cells were not labelled by SCRINSHOT we used sections of the same lung labelled in the SCRINSHOT experiment for immunostaining to detect Sftpc protein expression in the RFP positive and negative cells. Similar to the SCRINSHOT result 3.06% of the 1111 Sftpc positive cells were RFP fluorescence negative (false negative for RFP fluorescence) and 1.1% of the 1088 RFP fluorescence positive cells were negative for Sftpc staining (false positive for RFP fluorescence) (Figure 4A). This analysis indicates that SCRINSHOT and immunofluorescence experiments detect the same percentage of Sftpc negative RFP fluorescence positive cells. The 1% of RFP positive cells that remain unlabeled by SCRISHOT or Sftpc immunofluorescence could be lineage-labelled AT2 cells that differentiated to AT1 cells and turned off the expression of the *Sftpc* gene during the 3-week period between the labelling and lung harvesting [2, 3].

The sensitivity of the method is addressed by two tests. First, the Spearman correlation of SCRINSHOT signals in RFP fluorescence positive cells with scRNA-Seq counts of AT2 cells [4], including low, medium and high abundance transcripts. The 0.9511 ρ-value indicates that SCRINSHOT quantitatively detects low (Spry2 and Axin2), intermediate (e.g. Lgi3, Fgfr2, Ager, Etv5) and highly expressed genes (Sftpc and Lyz2). The numbers of SCRINSHOT dots are proportional to the transcript counts generated by Smart-Seq2 scRNA sequencing at both low and high levels of gene expression.

Second, we showed that SCRINSHOT is sensitive enough to reproducibly detect low-expressed genes encoding transcription factors, like *Etv5, Ascl1, Tp63, Sox2, Sox9* etc (Figure 5, 6 Suppl. Figure 5, 6, 7,10, 11).

While sensitivity was not assessed for moderate or lowly expressed genes, the images provided show very few puncta (on the order of 2 or 3) for many transcripts. For instance, in Supplemental Figure 2B, there is a large heart blood vessel which is only sparsely decorated with puncta for PECAM1.

We answer the reviewers’ general statement on sensitivity above. Coming to the performance of SCRINSHOT in *Pecam1* detection in the heart, we addressed this concern by a new experiment where we used several endothelial cell markers, *Pecam1, Cldn5, Cdh5*, the smooth muscle marker *Acta2*, and the epithelial cell markers *Scgb1a1* and *Sftpc*, as negative controls. (Revised Suppl. Figure 4). As expected, we detected the endothelial markers at the internal surface of the vessels, where endothelial cells are localized. *Acta2* was detected in the vascular smooth muscle cells surrounding the endothelium. Also, we detected scattered endothelial cells in the myocardium, as previously described [5] (arrow in Revised Suppl. Figure 4A). In contrast to smooth muscle cells, endothelial cells in cross-sections are very thin and therefore the signal for endothelial markers is sparser than the *Acta2* signal. We did not detect any expression of the abundant lung markers *Scgb1a* and *Sftpc* in the heart tissue. These results show that SCRINSHOT can detect endothelial markers at their expected position, supporting its specificity and sensitivity.

2) The authors provide some experiments showing that there is no signal when either the padlock cannot be ligated or the SplintR ligase is omitted. While these experiments demonstrate that a circle must be formed in order for signal to be produced, they do not substitute for control experiments showing that there is no significant off-target hybridization.

In all RCA-based methods like SCRINSHOT, IS [6], StarMAP [7] and PLISH [8], the signal can be only detected after padlock probe ligation and RCA. Unlike direct single molecule RNA FISH methods, off target hybridization of the padlock does not produce signal, unless ligation and amplification of the padlock occurs. The detection probes recognize the sequence of the padlock probes, and not the sequence of the targeted mRNA. The thousand-fold amplification of the padlock by the RCA reaction diminishes the probabilities of spurious detection of irrelevant mRNA sequences by off target hybridization.

The authors do perform a blocking oligonucleotide experiment for Secretoglobin 1a1 which has a nice result. However, when they actually quantify the specificity using surfactant C, this reveals a 7.4% false positive rate, which in my opinion is far too high to be acceptable for any staining technique.

In addition to *Scgb1a*, which is expressed in airways, we used the tamoxifen induced recombination to Sftpc-CreER;Rosa-Ai14 mice as a standard to assess SCRINSHOT in another cell type. As we explained in the text, single tamoxifen injections commonly label less than 100% of the cells expressing Sftpc-CreER [3, 9]. Thus, the estimated 7.4% false positives do not reflect the specificity of SCRINSHOT but also the inefficiency of cell labelling. In a new experiment, where we employed an anti-SftpC antibody to evaluate the recombination efficiency in sections of the same lungs used for SCRINSHOT analysis, the recombination efficiency was 97%. 3.06% of the positive cells for Sftpc protein were negative for RFP fluorescence (Figure 4A). Taken together this suggests that the rate of false positives in this test is less than 5%.

Supplemental Figure 4A-B encapsulate how poor the specificity of Scrinshot is, where most cells in a cluster that express the neuroendocrine marker Ascl1 (in panel A) co-express the Club cell marker Secretoglobin 1a1 (in panel B). Clearly, one of the markers is spuriously labeling the cells, since they should be complementary, rather than co-expressed.

The revised Suppl. Figures 6 A and B show *Scgb1a1*, *Ascl1* and *Cgrp1* expression in a neuroepithelial body (NEB). The *Ascl1* and *Cgrp1* signal is confined in the NEB and does not extend in the surrounding secretory cells. This further argues for the high specificity of SCRINSHOT. We assume that the reviewer is concerned with the presence of *Scgb1a1* signal in the area of cells expressing neuroendocrine markers. The NEBs are surrounded by secretory cells and also contain variant club cells expressing lower levels of the *Scgb1a1* ([10] and Figure 1 in [11]). Additionally, neuroendocrine cells express low levels of club cell markers, including *Scgb1a1* and *Scgb3a2*, as indicated by the scRNA-Seq dataset of Montoro et al., [12] (Suppl. Figure 9 D, E, F) and the data by Ouadah et al., [13].

To illustrate the cellular complexity of NEBs, we first used antibodies against Scgb1a1, CGRP and E-cadherin markers in conjunction with confocal microscopy. E-cadherin and DAPI stainings showed that the cells in the NEBs are tightly packed and interdigitated. Optical sections through the NEB show that 3 cells defined by the membrane marker E-cadherin in this NEB, co-express CGRP and Scgb1a. Thus, at the protein level a subset of NEB cells seems to co-express neuroendocrine and secretory markers (Suppl. Figure 7B).

We further analyzed the same NEB shown in Supplementary Figure 4 in the first version by confocal microscopy. In the projection image we detect some co-localization of *Scgb1a1* and *Ascl1* transcripts. Analysis of the individual optical sections however, shows that the *Scgb1a1* dots mark different cells than the *Ascl1* ones. In a few cells we detected a weak overlap especially at the apical part of the *Ascl1^pos^* neuroendocrine cells (Suppl. Figure 7 A). We interpret that the signal overlap detected in both the antibody and in the SCRINSHOT staining is due to the tight packing and interdigitation of the cells in the NEBs. Alternatively, the signal overlap could be due to some weak expression of *Scgb1a* in neuroendocrine cells as also suggested by the single cell sequencing analysis of Montoro et al., [12] and Ouadah et al., [13].

Another striking example is the type I marker AGER in Figure 6A for which puncta are shown in all epithelial cells, including most of the surfactant C positive cells which are putative type II cells.

Revised Figure 5D shows that *Ager* transcript is moderately expressed in AT2 cells of the analyzed scRNA-Seq dataset [14]. Also, the inducible knock-in Ager reporter (Ager-CreER^T2^: <https://www.jax.org/strain/032771>) labels not only AT1 but also AT2 cells, after induction of recombination in adult mice [15]. As discussed for NEB cells above, epifluorescence microscopy cannot exclude that the moderate Ager signal assigned to AT2 cells derives from adjacent AT1 cells.

The authors seem to be aware of the extent of this specificity problem, because in their definitions of each cell type, they permit detection of one incompatible marker as long as there are two appropriate markers. This is highly contrived and betrays the high level of false positive signal produced by Scrinshot.

Numerous experiments in the previous and in this revised version show that SCRINSHOT does not produce false positive signals unless tissue morphology prevents the distinction of cell borders. The application of combinatorial annotation criteria was used to show that we can apply previous information from scRNA-Seq to spatially validate the already identified cell-types, in space. The strategy for cell type identification, using both positive and negative markers, is common for cell type identification, for example in FACS experiments.

scRNA-Seq analysis generates lists of hundreds of selective combinatorial markers to define cell types. SCRINSHOT typically relies on panels of 2-3 markers per cell type, and we show that our analysis confirms the scRNA-Seq analysis (Suppl. Figure 9 B and E). In our experiments, the use of positive and negative annotation criteria provides a strict method for grouping the analyzed cells.

3) The heat map in Figure 7C includes a very large number of airway cells, but close examination of the profiles reveals very poor performance of Scrinshot, presumably due to the addition of poor segmentation on top of the low specificity. The populations are mostly distinguished by very high expression of a single canonical marker, and it does not appear the other transcripts influence the clustering, since looking at the expression of most markers across the cell types shows very poor correlation with the cell type in which it is known to be expressed. For instance, multiple club cell markers are inappropriately detected in basal and NE cells (e.g. Scgb1a1, 3a1, Reg3g). Also, apparently no ionocytes emerged (they are supposed to be coded blue in "Type" category) and CFTR expression is found scattered amongst cells belonging to most types.

The characteristic markers for the analyzed cell types are selective (higher expression levels and expressed in a higher number of positive cells in a cell cluster) but not unique for the corresponding cell-types. To illustrate this, we used the original cell annotation of the dataset from Montoro et al., [12] and show the percentage of positive cells and the expression values of each selected marker in the corresponding cell-type after a Seurat v3.1 analysis. Among the four selected ionocyte markers, only *Foxi1* is expressed more selectively in ionocytes. *Cftr* is expressed in 50% of the annotated ionocytes and in other cell types including club and basal cells. *Tfcp2l1* and *Ascl3* are expressed by many other cells, including club cells.

The good correlation of marker expression between SCRINSHOT and scRNA-Seq (Supplementary Figure 9 Band E) indicates that the detected signal is real and captures the gene expression heterogeneity of the analyzed cells *in situ*. The hierarchical clustering of the Figure 6C, is based on 29 analyzed genes to capture 6 cell types defined by scRNA-Seq analysis, which is based on hundreds to thousands of genes. The majority of the cells belonging to the identified cell types are clustered together (eg. goblet, club and tuft cells) in Figure 6C and they are identified at the expected positions (Figure 6 B and C). The low expression of the selected ionocyte markers, in addition to their expression in other cell types (e.g. *Cftr*, see Suppl. Figure 9 F and G) obstructed the clustering of these few cells together in our dataset.

Interestingly, the hierarchical clustering of all cells that express more than 3 dots of any combination of the analyzed genes, clustered 4 of the identified ionocytes together (insert in Suppl. Figure 9 C). These results indicate that targeted analysis of ionocytes, including more positively and negatively selective genes would allow better clustering of these cells but that was not the purpose of that study.

Regarding segmentation, we are aware and show that nuclear-based cell segmentation can introduce errors because some dots are wrongly registered to adjacent cells. Nevertheless, the detected RNA molecules of a given cell will be more numerous and closer to its nucleus than to other neighboring nuclei.

4) The authors cite many of the multiplexed in situ and spatial mapping technologies that have been published over the last few years, but rather than comparing the performance of Scrinshot against any of these, they present data benchmarking Scrinshot against cDNA-based in situ, an early technology that was abandoned because of its poor performance. All contemporary approaches to multiplexed in situ hybridization rely on RNA detection, for instance branched-chain (RNAscope) and the two most closely related to Scrinshot (StarMAP and PLISH) that also employ rolling circle amplification. So, if the authors want to demonstrate that Scrinshot is an advance for the field, they must compare its performance to a current standard in the field, not an early-generation technology that has been far surpassed.

The need for spatial mapping of identified cell types and states by single-cell RNA Sequencing has driven to the development of many technologies for multiplex *in situ* hybridizations [16]. Prof. Nilsson is one of the pioneers of the field with *in situ* sequencing [6]. His laboratory continuously improves the technology that allows high multiplexity [4] and detection of mutations, including SNPs. Considering that we have developed SCRINSHOT based on *in situ* sequencing to increase sensitivity but sacrifice high multiplexity and SNP detection, we decided to compare only these two methods. The suggestion of Reviewer #1 to compare SCRISNHOT with other methods, not established in our laboratory, is beyond the purpose of our present work. SCRINSHOT is more straightforward than STARMAP and PLISH and provides higher multiplexity and is much cheaper than RNAscope. We provide a detailed tutorial for all the steps of the protocol, to facilitate its successful reproduction by anyone with molecular biology background, if it is desired.

Reviewer #2:

The authors present a spatial-transcriptomic technique named SCRINSHOT that aimed to address current limitations of spatial-transcriptomics. Long (40 nt) gene-specific padlock probes are hybridized to PFA-fixed sections using a stringent protocol, followed by SplintR ligation of bound probes and phi-29 rolling circle amplification (RCA) to improve the signal-to-noise ratio. RCA products were detected using fluorescent-labelled oligos that bound the gene part of the padlock probe. Multiple rounds of hybridization allowed for the detection of ~ 30 genes (potentially 50 genes) in several thousand cells. The biological correlations used to validate SCRINSHOT are convincing and the level of detail in the protocol, from probe design to data analysis, is exemplary. Other techniques can detect more genes, some in 3D and at single molecule resolution, with examples of each referenced in the manuscripts introduction so this technique does not significantly outperform current state-of-the-art techniques in the field. However, the ease of SCRINSHOT implementation and lower equipment requirements will be of broad interest to labs that want to perform spatial-transcriptomics techniques and justifies publication in PlosBiology as a methods paper. However, I still have questions that need to be addressed before supporting acceptance of the manuscript.

Comments
● In my opinion the main novelty of this technique is ease of implementation. I suspect SCRINSHOT is cheaper compared to commercially available kits such as 10X Visium or RNAScope, providing a cost-effective way for labs to perform spatial transcriptomic. The authors echo this point in line 378; However, it is not sufficient to say SCRINSHOT is low cost without a detailed cost comparison vs commercially available spatial transcriptomics kits. The authors must include a detailed cost comparison so readers can decide if the reduced cost of SCRINSHOT justifies not using a commercial kit.

The current cost of SCRINSHOT for 30 genes (3 probes/gene) is 350 or 650 SEK (32-59 Euros), depending on the size of the tissue sections (Table 1 and Additional file 4 inserted into the supplemental protocol).

| **Table 1** | | |
| --- | --- | --- |
|  | **100μl Reaction** | **200μl Reaction** |
| **SecureSeal chamber** | 46.6 | 40.8 |
| **Blocking reaction** | 18.7 | 37.3 |
| **Padlock probe reaction (90 probes)** | 55.1 | 110.2 |
| **SplintR ligation reaction** | 64.6 | 129.2 |
| **RCA reaction** | 30.4 | 60.7 |
| **Detection oligo reactions (30 genes)** | 134.8 | 269.6 |
| **Total (SEK)** | **350.2** | **647.9** |

The commercially available methods are not directly comparable to SCRINSHOT because RNAScope can detect up to 12 genes and the resolution of the Visium 10X Genomics is not sufficient to recognize single cells (55 μm spot diameter and 100 μm distance between spot centers). Nevertheless, we estimate the cost of Visium at 11000 SEK (Price for Visium Spatial Gene Expression Slide & Reagent Kit and indexes per reaction. The sequencing cost is excluded) and 7850 SEK for RNAScope (Price for the HiPlex 12 reagent kit and probes for 12 genes.) To read more genes additional reactions and probes would be needed in sequential tissue sections.

In conclusion, SCRINSHOT is at least 10-fold more economical than the two commercially available solutions, and provides single cell resolution and higher multiplexity than the commercial RNAScope alternative. The additional advantage of SCRINSHOT compared to RNAScope is that the RCA signal amplification facilitates no signal loss over multiplex detection cycles (revised Suppl. Figure 6).

● The spatial mapping of AT2 and AT1 cells in the distal lung, shown in Supplementary Figure 7, differs from work published by Tushar Desai's lab showing AT1 cells adjacent to AT2 cells using IF and lineage tracing (DOI: 10.1126/science.aam6603). The simplest explanation is SCRINSHOT is not highly efficient resulting in false negatives. A detailed explanation about this discrepancy is required.

Nabhan et al., [17] have described the niche of an AT2 stem cell in the adult mouse alveolus. They found that a subpopulation on AT2 cells is in close proximity to a fibroblast cell type, that provides Wnt5a to the epithelial cells and retain their stemness. The AT2 receiving Wnt5a upregulate Axin2 as expected. In our experiment, in the Supplementary Figure 12, we included probes distinguishing AT1 and AT2 cells but not fibroblasts. In the cross section of the alveolus shown in the figure we detected distinct AT1 and AT2 cells. The cells not expressing markers could be fibroblasts or endothelial. We addressed the issue of false negatives and false positives in the Figures 4 and 5.

● No reference for Sftpc and Napsa as AT2 markers (e.g. <https://www.ncbi.nlm.nih.gov/pmc/articles/PMC5135277/pdf/jciinsight-1-90558.pdf>)

We are sorry for omitting this reference. We have added it to the revised text.

● Crowding of spots (proxy for a single RNA molecule) was described as an issue, yet in Figure 4 the number of dots was quantitated to perform correlation analysis with lineage-reporter expression with no explanation how crowding was overcome to allow such quantitation. Is crowding a gene specific issue (e.g. Scgb1a1)?

We noticed the crowding problem in the experiment shown in Figure 2, where we detected the expression of *Scgb1a* and *Actb* in the airways. Crowding is evident in cells expressing very high levels of any targeted gene. To address the problem of *Scgb1a* signal crowding we reduced the number of the padlocks and diluted the padlock probes. To establish that crowding is not probe specific but is directly dependent on the levels of gene expression we included a panel showing the detection of *Scgb1a* in the proximal and in the distal airways (Suppl. Figure 2A). Crowding is detected in the distal cells, which are known to express higher levels of the Scgb1a protein [18].

In Figure 4, the *Sftpc* signal dots were distinct and allowed quantification. The comparison of the SCRINSHOT number of dots to the scRNA-Seq counts shows that the detected gene expression levels by both methods are proportional. To provide an estimation of the saturation levels of our method, we used two genes expressed on intermediate levels, *Etv5* and *Cyp2f2* to calculate the average size of SCRINSHOT dots. The average *Etv5* dot size was 0.55µm^2^ and for *Cyp2f2* was 0.57µm^2^. Considering that the average cell-ROI size is 44.5 µm^2^, the expected maximum number of dots that can fit in this area without overlap is approximately 80. The maximum number of detected dots for *Sftpc* is very close to 80 but the average number of dots per cell is 35.44, indicating that saturation levels have not been reached for that highly abundant marker (Suppl. Figure 2B).

We conclude that SCRINSHOT gives quantitative measurements for highly expressed genes like *Sftpc* but when expression is extremely high like *Scgb1a* in distal airway cells, its performance is compromised because of signal crowding. Reduction of the number of padlock probes per gene, from the default three to one, and/or their concentration can be used to compensate the crowding problem, similarly to our approach for *Scgb1a1*, that was detected with one probe and 0.01µM concentration (the default is 0.05µM). As a rule of thumb, scRNA-Seq data can be used to estimate transcript abundance in different cell types and guide the choice of padlock probe number.

● Line 158: Similar Actb expression in all slides was mentioned in the text but not shown.

In the legend of Figure 2, we introduced the p-values of a multiple comparison test indicating no statistically significant difference between the groups, regarding the *Actb* detected dots per cell.

Reviewer #3:

In this manuscript, the authors report a novel approach to multiplexed in situ mRNA detection, based on a combination of hybridization using padlock probes, SplintR mediated ligation, RCA amplification and detection using fluorochrome-conjugated oligonucleotides. This approach allows detection of up to 30 probes with a good dynamic range for gene expression levels of the detected transcripts on PFA-fixed tissue sections of human and mouse lung. The paper is generally wel written, and the method is a major advance over existing methodologies such as MERfish and In Situ Sequencing, and will be of importance to generate spatial maps of cell types and states when combined with scRNA-Seq or snRNA-Seq data, which is an urgent need for atlassing studies of healthy or diseased tissues or in model organisms.

However, I also have some concerns and questions regarding some of the data, the analysis and the conclusions reached by the authors. In figure 2, the authors show data on the promiscuous ligation of the SplintR ligase, which might render aspecific signals. A single mismatch was introduced and shown to give strongly reduced signal, but not absence of signals. Given this effect, it would seem of relevance to explore this a bit further, and look at other mismatched probes (single mismatches in a slightly different sequence context, and involving other nucleotides), to allow a somewhat more systematic evaluation of the specificity of the technique.

The tolerance of SplintR to single mismatches at the ligation site has been previously reported [19-21]. To address Reviewer´s #3 concern, we introduced point mutations at the ligation site of two additional padlock probes and analyzed the obtained signal. A C>T exchange at the 3’-arm of the *Cyp2f2* padlock probe caused 50% reduction of the obtained dots (Suppl. Figure 3 A-D). Importantly, all the detected *Cyp2f2* dots were localized in airway cells, in contrast to the signal from *Actb*, which was also detected in alveolar cells, as expected. Thus, *Cyp2f2* signal distribution provides additional evidence that the specificity of the SCRINSHOT is predominantly based on the hybridization of the padlock probe arm sequences and not by the 2 nucleotides at the ligation site. Similarly, the introduction of a T>C mutation at the 5’-arm of *Etv5* padlock probe reduced the signal 10 times, as indicated by the average of *Etv5* dots in more than 800 alveolar cells of the same area in two sequential sections. The *Actb* internal control gave no statistically significant differences (Suppl. Figure 3 E-H).

In summary, we confirmed the previously reported tolerance of SplintR ligase for single nucleotide mismatches at the ligation site [19-21]. The new and previous results show that the specificity of SCRINSHOT largely depends on the stringent hybridization of the 20 nucleotides long padlock probe arms (40 nucleotides in total) and less on the specificity of the used ligase.

In figure 4 and 5 the authors evaluate the quantitative detection power and the cell-type specificity of the SCRINSHOT methodology, using the RosaAi14 reporter mouse backcrossed to a Sftpc-CreER strain to induce recombination of the reporter in type-2 cells after tamoxifen application. However, these two figures use semi-overlapping methodology or even data and are quite confusing as presented currently. In Figure 4, RFP is used to identidy AT2 cells, whereas in Figure 5 this is performed by Sctpc detection using SCRINSHOT. In figure 4, the correlation between SCRNSHOT RFP mRNA detection and RFP fluorescence (protein levels) is shown to be quite good, although in the CreER negative mice, some background in this channel was caused by red blood cell autofluorescence. It remains unclear, however, whether all cells were analyzed, or only the cells at a specific cut-off for the RFP fluorescence signal. Also, for clarity the authors should not refer to the RFP (transgene) fluorescence as 'endogenous'.

We apologize for the confusing writing of this part. Overall, we rewrote the section of the manuscript describing the experiments in revised Figures 4 and 5 to improve the clarity. Regarding the specific question on Figure 4, its main purpose was to correlate the RFP transgene-fluorescence with the *RFP* detected mRNA. No, cutoff was applied, all the cells of the analyzed areas were used (“n” in the plots indicate the numbers of the analyzed cells).

In Figure 5, the authors go on to analyze AT2 cells by using Sftpc expression using SCRINSHOT detection as a means of AT2 cell identification (5A,B). This is initially confusing, so it could be better explained in the text on page 10. Just stating the relevant research question at the beginning of the paragraphs discussing the results presented in fig 4 and fig 5 would already be very helpful.

Thank you, we tried to improve the text according to the helpful suggestion.

In figure 5C, the authors use the same dataset but now define AT2 cells by RFP protein expression, and evaluate Sftpc expression by SCRINSHOT. Here, RFP+ Scftpc- cells are considered false negatives (for Sftpc expression) and RFP- Sftpc+ cells are considered false positives (for Sftpc expression). In Fig5D, his is reversed again (RFP false-positive and false-negative for Sftpc+ cells). This seems - as also mentioned by the authors - not the best way to identify AT2 cells, as lineage tracing using genetic markers has a considerable false-positive and false negative rate itself.

Rather, using another AT2 marker as presented in fig 6 might be a good way to estimate false-positive and false-negative rates (see also comments at that figure). All in all this figure and its data description is complex, confusing and in my opinion not the best analysis that could be done - some more evaluation of concordance between SCRINSHOT probes for AT2 cells might be helpful here, circumventing the use of the lineage trace. The conclusion of the authors should be about the performance of their technique, not of the lineage trace of the AT2 cells (which is now a significant confounding factor).

This is also a good point and we tried to improve our evaluation by first investigating false positives and false negatives in the *Sftpc-CreER*;*Rosa-Ai14* lineage tracing model. In the revised version, we used sections from the same lungs (*Sftpc-CreER*^neg^;*Rosa-Ai14*^pos^ and *Sftpc-CreER*^pos^;*Rosa-Ai14*^pos^) used for SCRINSHOT analysis and analyzed them for anti-SftpC immunostaining and RFP fluorescence. 3.06% of the 1111 Sftpc positive cells were RFP fluorescence negative (false negative for RFP fluorescence) and 1.1% of the 1088 RFP fluorescence positive cells were negative for Sftpc staining (false positive for RFP fluorescence) (Figure 4A). RFP false negatives can be attributed to suboptimal efficiency of Cre induction and RFP false positives to leaky activation of Cre and *Rosa* locus (details in <https://www.jax.org/strain/007914>) or differentiation of AT2 to AT1 cells during the 3 week period between Tamoxifen induction and analysis. These results, indicate that the RFP reporter in this experiment labels true Sftpc positive cells, with 97% fidelity.

In the same lung, we first examined the ability SCRINSHOT to detect the *RFP* mRNA in RFP fluorescent cells. We analyzed 14167 cells in a large area including airways and alveoli. The RFP fluorescence intensity (Raw Integrated Density) per cell correlated (R^2^=0.7233) with the number of SCRINSHOT-detected *RFP* mRNA molecules, (Figure 4 C). By contrast, there was no correlation between the *RFP* SCRINSHOT signal and the RFP fluorescence (R^2^=0.0671) in 3355 cells of the *Sftpc-CreER*^neg^-*Rosa-Ai14*^pos^ control lung. As expected, in the 1008 analyzed *wild type* cells, the correlation of RFP fluorescence and SCRINSHOT dots was very low. Upon closer inspection most of the signal in this lung was due to high auto-fluorescence of red blood cells (Figure 4 B, C). We conclude that the RFP SCRINSHOT signal directly correlates with the activation of the fluorescent reporter.

To provide another quantitative estimation about the specificity and sensitivity of our method, we used the protein RFP fluorescence as “ground truth” and separated the alveolar cells in RFP fluorescence positive and negative cells. Considering that Cre-mediated recombination is under the control of the endogenous *Sftpc* promoter, we measured the detected *Sftpc* and *RFP* mRNA transcripts in both groups. In 1425 RFP fluorescence positive cells, 1.1% contained 0-3 *Sftpc* dots (Figure 4D) and 1.05% 0-3 *RFP* dots (Figure 4E), which were considered as false negative.

Among the 6848 RFP fluorescence negative cells, 5.53 % were positive for *Sftpc* and 4.95% for *RFP* SCRINSHOT dots and were considered as false positives (Figure 4F, G). As a result, the analysis of the inducible knock-in reporter shows that the SCRINSHOT *RFP* and *Sftpc* signals highly correlate with RFP fluorescence intensity, indicating high sensitivity and specificity. The high specificity is additionally supported by the absence of *RFP* SCRINSHOT signal in the analyzed wild-type lung (Figures 4B) in the same experiment.

We followed Reviewer’s #3 suggestion to use another AT2 marker for definition of the AT2 cells. Thus, we also based our evaluation on moderately expressed genes like *Etv5*, encoding a transcription factor, that is specifically expressed in AT2 cells [22]. We classified the analyzed cells, according to their *Etv5* positivity. 1431 cells were scored as *Etv5*^pos^ (>3 dots) while the remaining 12736 were *Etv5*^neg^. *Sftpc* mRNA transcripts were detected in 99% of the *Etv5*^pos^ cells. Similarly, 94.8% of the *Etv5*^pos^ cells were *RFP* positive. This analysis, indicates that SCRINSHOT’s specificity is high (95%), even for moderately expressed genes (e.g. *Etv5),* allowing efficient cell-type identification.

In a complementary test, we analyzed a published SmartSeq2 scRNA-Seq dataset of AT2 cells (GSE118891) [14]. There, 96.8% of the AT2 cells have more *Etv5* scRNA-Seq counts than the club cells in the same dataset and that 12% of them express low levels of the gene (less than 1 standard deviation from average number of counts). This low expressing AT2 cell subset may reflect technical variation or real, biological variability [14]. On the protein level, only 50% of the adult mouse AT2 cells are positive for Etv5 [22]. Thus, if SCRINSHOT were as sensitive as single cell sequencing by SmartSeq2, we would expect that 3-12% of the RFP fluorescence positive cells, to be detected without *Etv5* dots. Our counting, did not detect *Etv5* signal in 3.5% of the 1425 RFP fluorescence positive cells. Additionally, 14.8% of the RFP fluorescence positive cells had 0-2 *Etv5* dots, which is less than the standard deviation of the average *Etv5* dots (Suppl. Figure 8). These results provide additional evidence that SCRINSHOT is in precise agreement with scRNA-Seq methods and that its sensitivity is also high for moderately expressed genes.

In figure 6, a number of AT2 probes are used, which are tested for correlation to a published AT2 single-cell dataset. Expression was detected in the RFP+ cells. The selected scRNA-seq dataset seems arbritrary and small (a few hundred cells sequenced) - why was this specific dataset used?

We used the specific dataset because it was available at the time of the experiments and because (Smart Seq2) is considered more sensitive than drop-seq [23].

In addition to correlation to scRNA-seq data it would be very helpful to see correlation of AT2 markers to each other when detected by SCRINSHOT in the same tissue section. If serial hybridizations were done to generate the data for figure 6 (so it seems looking at the figures), his could be analyzed by the authors, and these data would be highly relevant to evaluate the performance of SCRINSHOT.

We followed the reviewer´s suggestion. We used *Sftpc* [1], *Lyz2* [24], *Etv5* [22] and *Napsa* [25], as a panel of AT2 selective markers and performed pairwise correlations of each marker with the rest and with the RFP SCRINSHOT counts and protein fluorescence intensity. We also included the club cell markers *Scgb1a1* and *Cyp2f2* [26], as negative controls. The Spearman correlation coefficients (ρ) of the comparisons between *Sftpc*, *Napsa*, *Etv5* and *RFP* spanned between 0.81 and 0.9 (maximum 1), indicating high correlation. *Lyz2* correlates very well with the above AT2 markers but there are *Lyz2* single positive cells, that likely correspond to alveolar macrophages [27]. The highest correlation of the analysis (ρ: 0.93) was between *Scgb1a1* and *Cyp2f2* because of their exclusive expression in club airway cells. As expected, they anti-correlated (negative ρ values) with the AT2 markers and the RFP fluorescence (Figure 5 B). This indicates that SCRINSHOT can detect multiple selective markers in the same cell, in a quantitative manner.

In figure 7 the authors present a true spatial map using multiplexed SCRINSHOT. These data are very interesting and will likely represent a highly relevant technological advance to the field. However, some technical data are lacking that are relevant for the performance of the methods. For instance, what is the overlap between the markers for the same cell type? How many cells per cell type show 1 2 or 3 markers for that cell type?

We used the combinatorial expression of top selective markers from published scRNA-Seq experiments to annotate the cells, as it is common in single cell analyses [12]. The general scheme was 1) selection of markers from publication. 2) Assignment of cells for marker positivity based on SCRINSHOT counts above a threshold for each marker. The threshold was set at 10% of the maximum value of the marker gene counts. But for highly abundant genes (>30 dots) we used 3 dots as threshold, based on the low false positive rate of the method 3) A cell was considered to belong to a category if it was positive for at least 2 of the selective markers and only expressed up to one marker of the other categories. For example, to consider a cell as basal, it should score positive for, at least two, of the *Krt5*, *Pdpn* and *Trp63*. Among the annotated basal cells, 82.9% had ≥1 dots for two of the markers and 17.1% for all 3. For ionocytes we used *Ascl3*, *Foxi1*, *Cftr* and *Tfcp2l1* as markers. 42.9% of the annotated ionocytes showed ≥1 dots for two of the markers and 57.1% were positive for three or more markers. For tuft cells, we used the *Gng13*, *Trpm5*, *Alox5ap*, *Six1*, *Sox9* and *Il25* as markers. 63.8% of the annotated tuft cells had ≥1 dots of two of the corresponding genes and 36.2% for three or more. For goblet cells we used *Tff2*, *Gp2*, *Pax9*, *Lipf*, *Dcpp3*, *Dcpp1*, *Foxq1* and *Spdef* as markers. 41.9% of the annotated goblet cells had ≥1 dots of the *Tff2*, *Gp2*, *Pax9*, *Lipf*, *Dcpp3*, *Dcpp1*, *Foxq1* and *Spdef* and 58.1% showed ≥1 dots for three or more of these genes. We considered a cell as club, if it was positive (>3 dots) for Scgb1a1 only. 32.1% of the annotated club cells were positive for only that marker and they are mainly localized at the distal lung airway epithelium (Figure 6 A and C). In the trachea and proximal airways, 12.8% of the club cells also had ≥1 dots of one of the *Scgb3a1*, *Reg3g*, *Bpifb*, *Il13ra1* and *Lrg6* and 55.1% were positive for two or more markers (Suppl. Figure 9 A).

How many cells are stained by markers for different cell types? How were these dealt with in the analysis? How do the fractions of cells compare to available data from classical histology or singe-cell RNAseq studies?

In Figure 6 we counted 4436 cells. Of these, 4115 were expressing any of the selected 29 markers. 2181 (49%) were classified in the originally published cell types. The remaining 51% are likely ciliated cells, it was published that 50% of the tracheal cells are belong to this cell type [28]. To answer how many cells of a particular cell type also express markers for another cell type we analyzed all the annotated cells and counted all signal dots for each of the analyzed genes. We then plotted the percentage of cells in each cell type expressing one or more counts of all genes (Suppl. Figure 9 B). As expected, there is some co-expression of markers, which is more prominent for the secretory, club-cell markers Scgb1a, Scgb3a1, Reg3g, Bpfb1and Muc5b. This could be explained in two ways, either this marker combination and the assay is not specific or that this marker signature is selectively expressed at higher levels in club cells and at lower levels in the remaining cell types. To address this, we used the published dataset of Montoro et al., [12] and analyzed the cells with Seurat v3.1 [29] to first retrieve and plot the percentages of positive cells per cell-type, for all analyzed genes with SCRINSHOT (Suppl. Figure 9 E). In this analysis, we used the original annotation of the GSE103354 dataset. The heatmap of overlapping marker gene expression in different cell types was strikingly similar to the heatmap from SCRINSHOT excluding the possibility that the detected co-expression of club genes in other cell types are a SCRINSHOT-associated artefact. (Suppl. Figure 9 B and E). The Club cell markers *Scgb1a1*, *Scgb3a1* and *Reg3g* are also expressed by all cell types but their expression is higher in annotated club cells, with both methods. SCRINSHOT did not detect *Scgb1a1* in goblet cells possibly due to the reduced padlock concentration to prevent signal saturation in the lung club cells and because the goblet cells, being positive for club-cell markers were scored as “NA”, according to the strict annotation criteria.

To exclude the possibility that the low expression levels of the analyzed genes, in droplet-based dataset are background, we examined the FACS-based dataset of the same paper, using *Cftr* as an example. Similarly, low levels of *Cftr* expression were detected in club, basal and tuft cells, in addition to the higher expression levels of ionocytes (Suppl. Figure 9 G).

The new analysis indicates that none of the selected marker genes is uniquely expressed by one cell-type in both scRNA-Seq dataset and SCRINSHOT but they can function combinatorically, as markers of distinct cell types, based on their expression levels. SCRINSHOT can deal with that, since it can function quantitatively and discriminate cells with zero, low- and high-expression levels. Unsupervised (e.g. hierarchical clustering) and/or supervised characterization approaches (e.g. our cell-type annotation rule) can take into account the combination of the presence and absence of the analyzed genes to annotate the cells in different types and states, retaining the spatial information.

These sort of analyses would add greatly to the significance of the paper, especially since 2555 cells fal into the category of unknown/ciliated. What exactly does this mean? These cells are for instance very frequently observed in submucosal glands - what cell type would these be?

The majority of the introduced cell-type markers are selective for the corresponding cell type, but not unique, as explained above. The introduced annotation criteria, assumed that a given cell should be enriched for the genes of the corresponding cell-type and do not express or express at lower levels, markers of other cell types. For that reason, the “positivity” for a gene is determined by its maximum number of dots.

In Figure 6, we tried to apply a combinatorial marker expression rule to annotate the cells according to already known scRNA-Seq data but we did not include markers for all the cell types, that have been anatomically detected in submucosal glands and airway epithelium. As explained in the manuscript, ciliated cells are considered the main source of “NA” cells in the airway epithelium. They are also found in the submucosal glands, in addition to myoepithelial cells and neurons [30, 31] for which we had no markers. In addition to the lack of markers for all submucosal-gland cell type, we consider as the main reason for “NA” cells, the expression of club-cell markers by goblet cells (Suppl. Figure 9 C). But this became evident only after the bioinformatics analysis of the scRNA-Seq dataset of Montoro et al., [12]. As a result, the strict annotation criteria allowed the partial characterization of cells as goblet.

To provide a more holistic icon of the SCRINSHOT performance for spatial mapping of the analyzed cells, we included all the cells that express more than 3 dots of any combination of the analyzed genes for unsupervised cell grouping, using hierarchical clustering. Many of the previously excluded cells, as “NA” are now clustered together with the annotated cells, indicating the strictness of the utilized criteria for cell type annotation, at the original analysis (Suppl. Figure 9 C). Cluster 1 contains the majority of club cells of proximal and distal intra-lobar lung airways and cluster 3 contains the club cells of the trachea. Cluster 2 and 4 are the submucosal-gland goblet cells. Cluster 5 is the most heterogeneous, since it contained the annotated ionocytes, basal and NE cells. Finally, cluster 6 contains the majority of the “NA” cells, that are localized in intermediate and distal trachea. Its left branch presumably corresponds to ciliated cells because of the absence of *Scgb1a1*. The presence of *Reg3g* and low *Scgb3a1* agrees with the scRNA-Seq data (Suppl. Figure 9 D and F). The right branch contains the rest of the tracheal club cells, that are negative for the *Bpifb1*, similarly to its expression in scRNA-Seq data (Suppl. Figure 9 D and F).

The proper choice of the panel of cell-type markers is essential for detection of the cells of interest, as in any targeted method. The fact that no unique but only highly selective markers were identified and used for the spatial mapping of the cell types [12], reveals the high heterogeneity of respiratory epithelium and points out the importance of combinatorial detection of as many markers as possible to spatially track the cells in the tissue.

The detection of specific cell types is a great asset in this technique, but if the majority of the cells is not assigned to a known cell type, he application of the technique will of course be limited. the authors need to discuss these cells, and the limitations of their approach. If the main problem is cell segmentation during the analysis, this should not be specific for this technique and authors would need to clarify this or compare to similar methods also hampered by cell segmentation accuracy during analysis.

SCRINSHOT can detect 30-50 genes in the same tissue section. This limitation, can drive to complete omission of markers for distinct cell-types, if there is no intend to study them, such as ciliated cells in our tracheal analysis. As explained, the corresponding cells will reasonably get scored as not-annotated but we do not consider that as a problem, because their percentage is similar to the expected percentage of that cell type (~50%), in the corresponding areas [28].

Regarding cell segmentation, we based our analyses on nuclei border drawing, because we did not have any indication of the real cell borders. This is far from perfect but we expect that the detected RNA molecules of a given epithelial cell will be closer to its nucleus than to other neighboring nuclei. The utilized 10 µm thick sections allow partial overlapping of nuclei and cells, resulting to false registration of dots to neighbors of the real expressing cells, but thinner sections will impact the quality of histology and sensitivity. Our approach of manual segmentation is the most time-consuming part of the analysis and we believe that automatic segmentation will provide a future solution. At present, our attempts with open access programs like Ilastik [32] or Cell Profiler [33] only scored a 50% success rate in segmentation of lung sections, presumably because of the elongated and overlapping cell shapes and the large cavities of the lung alveolar compartment. Recent advances in bioimage analysis field indicate that artificial intelligence (AI) approaches can be utilized as an automated, reproducible methodology to solve the problem of cell and nuclear segmentation, but still there is no applicable user-friendly algorithm, as described in [34], a summary of the results from a segmentation competition of 3,891 teams worldwide.

The majority of spatial transcriptomic methods use cultured cells, which allows easier identification of nuclear and cellular borders, than tissue sections [35-37]. When tissue sections are analyzed, then the expression is measured in areas and not cells [38, 39]. Only a few methods, like seqFISH [40] and osmFISH tried to use signal-assisted segmentation of the cells but that requires the use of highly expressed genes for all cells and assumes that positive cells for the same marker, will be clearly separated. As we show, confocal microscopy and higher magnifications may improve resolution but cost will also increase. Cell membrane staining, will be another complementary solution but the tissue treatment with organic solvents, during hybridizations significantly compromise lipid- or antibody-mediated labeling.

**Literature**

1.	Kalina, M., R.J. Mason, and J.M. Shannon, *Surfactant protein C is expressed in alveolar type II cells but not in Clara cells of rat lung.* Am J Respir Cell Mol Biol, 1992. **6**(6): p. 594-600.

2.	Barkauskas, C.E., et al., *Type 2 alveolar cells are stem cells in adult lung.* J Clin Invest, 2013. **123**(7): p. 3025-36.

3.	Rock, J.R., et al., *Multiple stromal populations contribute to pulmonary fibrosis without evidence for epithelial to mesenchymal transition.* Proc Natl Acad Sci U S A, 2011. **108**(52): p. E1475-83.

4.	Qian, X., et al., *Probabilistic cell typing enables fine mapping of closely related cell types in situ.* Nat Methods, 2020. **17**(1): p. 101-106.

5.	He, L., et al., *Preexisting endothelial cells mediate cardiac neovascularization after injury.* J Clin Invest, 2017. **127**(8): p. 2968-2981.

6.	Ke, R., et al., *In situ sequencing for RNA analysis in preserved tissue and cells.* Nat Methods, 2013. **10**(9): p. 857-60.

7.	Wang, X., et al., *Three-dimensional intact-tissue sequencing of single-cell transcriptional states.* Science, 2018. **361**(6400).

8.	Nagendran, M., et al., *Automated cell-type classification in intact tissues by single-cell molecular profiling.* Elife, 2018. **7**.

9.	Chapman, H.A., et al., *Integrin alpha6beta4 identifies an adult distal lung epithelial population with regenerative potential in mice.* J Clin Invest, 2011. **121**(7): p. 2855-62.

10.	Reynolds, S.D., et al., *Neuroepithelial bodies of pulmonary airways serve as a reservoir of progenitor cells capable of epithelial regeneration.* Am J Pathol, 2000. **156**(1): p. 269-78.

11.	Guha, A., et al., *Uroplakin 3a(+) Cells Are a Distinctive Population of Epithelial Progenitors that Contribute to Airway Maintenance and Post-injury Repair.* Cell Rep, 2017. **19**(2): p. 246-254.

12.	Montoro, D.T., et al., *A revised airway epithelial hierarchy includes CFTR-expressing ionocytes.* Nature, 2018. **560**(7718): p. 319-324.

13.	Ouadah, Y., et al., *Rare Pulmonary Neuroendocrine Cells Are Stem Cells Regulated by Rb, p53, and Notch.* Cell, 2019. **179**(2): p. 403-416 e23.

14.	Liu, Q., et al., *Lung regeneration by multipotent stem cells residing at the bronchioalveolar-duct junction.* Nat Genet, 2019. **51**(4): p. 728-738.

15.	Chung, M.I. and B.L.M. Hogan, *Ager-CreER(T2): A New Genetic Tool for Studying Lung Alveolar Development, Homeostasis, and Repair.* Am J Respir Cell Mol Biol, 2018. **59**(6): p. 706-712.

16.	Strell, C., et al., *Placing RNA in context and space - methods for spatially resolved transcriptomics.* FEBS J, 2019. **286**(8): p. 1468-1481.

17.	Nabhan, A.N., et al., *Single-cell Wnt signaling niches maintain stemness of alveolar type 2 cells.* Science, 2018. **359**(6380): p. 1118-1123.

18.	Dodge, D.E., et al., *Quantitative comparison of intracellular concentration and volume of Clara cell 10 KD protein in rat bronchi and bronchioles based on laser scanning confocal microscopy.* J Histochem Cytochem, 1993. **41**(8): p. 1171-83.

19.	Krzywkowski, T., M. Kuhnemund, and M. Nilsson, *Chimeric padlock and iLock probes for increased efficiency of targeted RNA detection.* RNA, 2019. **25**(1): p. 82-89.

20.	Krzywkowski, T. and M. Nilsson, *Fidelity of RNA templated end-joining by chlorella virus DNA ligase and a novel iLock assay with improved direct RNA detection accuracy.* Nucleic Acids Res, 2017. **45**(18): p. e161.

21.	Ho, C.K., J.L. Van Etten, and S. Shuman, *Characterization of an ATP-dependent DNA ligase encoded by Chlorella virus PBCV-1.* J Virol, 1997. **71**(3): p. 1931-7.

22.	Zhang, Z., et al., *Transcription factor Etv5 is essential for the maintenance of alveolar type II cells.* Proc Natl Acad Sci U S A, 2017. **114**(15): p. 3903-3908.

23.	Ziegenhain, C., et al., *Comparative Analysis of Single-Cell RNA Sequencing Methods.* Mol Cell, 2017. **65**(4): p. 631-643 e4.

24.	Desai, T.J., D.G. Brownfield, and M.A. Krasnow, *Alveolar progenitor and stem cells in lung development, renewal and cancer.* Nature, 2014. **507**(7491): p. 190-4.

25.	Xu, Y., et al., *Single-cell RNA sequencing identifies diverse roles of epithelial cells in idiopathic pulmonary fibrosis.* JCI Insight, 2016. **1**(20): p. e90558.

26.	Fanucchi, M.V., et al., *Pulmonary cytochrome P450 monooxygenase and Clara cell differentiation in mice.* Am J Respir Cell Mol Biol, 1997. **17**(3): p. 302-14.

27.	Abram, C.L., et al., *Comparative analysis of the efficiency and specificity of myeloid-Cre deleting strains using ROSA-EYFP reporter mice.* J Immunol Methods, 2014. **408**: p. 89-100.

28.	Toskala, E., et al., *Temporal and spatial distribution of ciliogenesis in the tracheobronchial airways of mice.* Am J Physiol Lung Cell Mol Physiol, 2005. **289**(3): p. L454-9.

29.	Stuart, T., et al., *Comprehensive Integration of Single-Cell Data.* Cell, 2019. **177**(7): p. 1888-1902 e21.

30.	Tata, A., et al., *Myoepithelial Cells of Submucosal Glands Can Function as Reserve Stem Cells to Regenerate Airways after Injury.* Cell Stem Cell, 2018. **22**(5): p. 668-683 e6.

31.	Lynch, T.J. and J.F. Engelhardt, *Progenitor cells in proximal airway epithelial development and regeneration.* J Cell Biochem, 2014. **115**(10): p. 1637-45.

32.	Berg, S., et al., *ilastik: interactive machine learning for (bio)image analysis.* Nat Methods, 2019.

33.	McQuin, C., et al., *CellProfiler 3.0: Next-generation image processing for biology.* PLoS Biol, 2018. **16**(7): p. e2005970.

34.	Caicedo, J.C., et al., *Nucleus segmentation across imaging experiments: the 2018 Data Science Bowl.* Nature Methods, 2019. **16**(12): p. 1247-1253.

35.	Rouhanifard, S.H., et al., *ClampFISH detects individual nucleic acid molecules using click chemistry-based amplification.* Nat Biotechnol, 2018.

36.	Raj, A., et al., *Imaging individual mRNA molecules using multiple singly labeled probes.* Nat Methods, 2008. **5**(10): p. 877-9.

37.	Chen, K.H., et al., *RNA imaging. Spatially resolved, highly multiplexed RNA profiling in single cells.* Science, 2015. **348**(6233): p. aaa6090.

38.	Svedlund, J., et al., *Generation of in situ sequencing based OncoMaps to spatially resolve gene expression profiles of diagnostic and prognostic markers in breast cancer.* EBioMedicine, 2019.

39.	Wang, F., et al., *RNAscope: a novel in situ RNA analysis platform for formalin-fixed, paraffin-embedded tissues.* J Mol Diagn, 2012. **14**(1): p. 22-9.

40.	Shah, S., et al., *In Situ Transcription Profiling of Single Cells Reveals Spatial Organization of Cells in the Mouse Hippocampus.* Neuron, 2016. **92**(2): p. 342-357.
